# Dynamic Nucleophilic Aromatic Substitution of Tetrazines

**DOI:** 10.1002/anie.202106230

**Published:** 2021-07-12

**Authors:** Tanausú Santos, David S. Rivero, Yaiza Pérez‐Pérez, Endika Martín‐Encinas, Jorge Pasán, Antonio Hernández Daranas, Romen Carrillo

**Affiliations:** ^1^ Functional Molecular Systems Group Instituto de Productos Naturales y Agrobiología (IPNA-CSIC) Avda. Astrofísico Fco. Sánchez 3 38206 La Laguna Spain; ^2^ Laboratorio de Materiales para Análisis Químicos (MAT4LL) Departamento de Física Universidad de La Laguna (ULL) 38206 La Laguna Tenerife Spain

**Keywords:** cage compounds, Diels–Alder reaction, dynamic covalent chemistry, photolysis, tetrazine

## Abstract

A dynamic nucleophilic aromatic substitution of tetrazines (S_N_Tz) is presented herein. It combines all the advantages of dynamic covalent chemistry with the versatility of the tetrazine moiety. Indeed, libraries of compounds or sophisticated molecular structures can be easily obtained, which are susceptible to post‐functionalization by inverse electron demand Diels–Alder (IEDDA) reaction, which also locks the exchange. Additionally, the structures obtained can be disassembled upon the application of the right stimulus, either UV irradiation or a suitable chemical reagent. Moreover, S_N_Tz is compatible with the imine chemistry of anilines. The high potential of this methodology has been proved by building two responsive supramolecular systems: A macrocycle that displays a light‐induced release of acetylcholine; and a truncated [4+6] tetrahedral shape‐persistent fluorescent cage, which is disassembled by thiols unless it is post‐stabilized by IEDDA.

## Graphical abstract

All the advantages of dynamic covalent chemistry and the versatility of the tetrazine moiety have been combined in a novel exchange reaction: The nucleophilic aromatic substitution of tetrazines (S_N_Tz) with phenols and alkyl thiols. Not only sophisticated structures could be easily synthesized, but more importantly, post‐functionalization by IEDDA or photolysis of the compounds show that S_N_Tz unleashes a huge potential of applications.

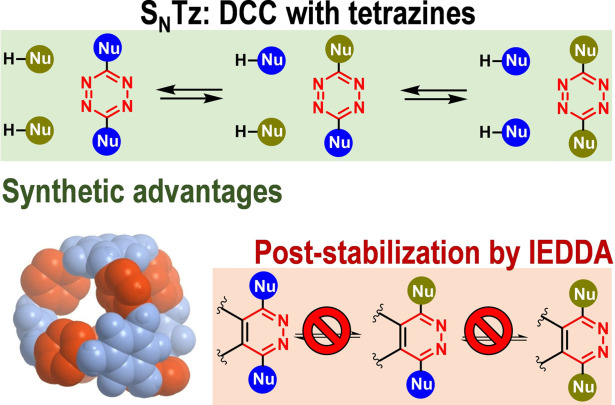

## Introduction

Dynamic covalent chemistry (DCC) has become a powerful tool in supramolecular chemistry and materials science. This is probably due to the ability of DCC to “error‐checking” and “proof‐reading”, as it is based on reversible covalent reactions under thermodynamic control. This allows to correct synthetic dead‐ends as it happens in non‐covalent chemistry, although with much more robust molecular systems.[^1^, ^2^, ^3^, ^4^] The relevance of DCC on current chemistry is clearly reflected by the number of reported application in the synthesis of sophisticated molecular architectures,[^5^, ^6^, ^7^, ^8^, ^9^] in polymer chemistry,[^10^, ^11^] in finding novel porous materials,^[12]^ or bioactive compounds,[^13^, ^14^, ^15^, ^16^, ^17^] and in systems chemistry.[^18^, ^19^, ^20^]

Even when there are well‐established reversible reactions such as imine chemistry,[^21^, ^22^] disulfide exchange,[^23^, ^24^] boronic linkages,[^25^, ^26^] olefin metathesis,[^27^, ^28^] and some other interesting examples,[^29^, ^30^, ^31^, ^32^, ^33^, ^34^, ^35^] there is still a limited set of reactions amenable to dynamic covalent chemistry. Therefore, scope and functionality of the molecular structures synthesized could be improved by new additions to the toolkit of DCC. Particularly interesting would be to envision a novel dynamic process which inherently displays relevant physico‐chemical properties and which allows for further functionalization.

In this regard, we thought that a good choice for a robust and versatile DCC reaction could be the nucleophilic aromatic substitutions (S_N_Ar) of tetrazines. In fact, S_N_Ar is known to be reversible in some cases,^[36]^ yet its dynamic nature has been overlooked almost completely.[^37^, ^38^] Obviously, S_N_Ar requires an electron‐poor aromatic ring to be involved. But this requirement offers the chance to explore tetrazines as a potentially optimal moiety for an extremely versatile dynamic process. Indeed, tetrazines are not only electron‐poor aromatic rings amenable to S_N_Ar, but they are also a very useful moiety.^[39]^ Thus, they have been profusely used as a way to attach different compounds and moieties by inverse electron demand Diels–Alder (IEDDA) reaction,^[40]^ which has found many applications in biology,[^41^, ^42^, ^43^, ^44^, ^45^, ^46^] and materials science.[^47^, ^48^, ^49^, ^50^, ^51^] Tetrazine is a valuable scaffold to build stimulating architectures such as corona[*n*]arenes.[^52^, ^53^, ^54^, ^55^, ^56^, ^57^] They also show an interesting redox performance,[^58^, ^59^, ^60^] and some of them display fluorescence.[^61^, ^62^, ^63^, ^64^] Even when it is known that some tetrazines, such as 3,6‐dichloro‐1,2,4,5‐tetrazine (**Cl‐Tz‐Cl**) can undergo S_N_Ar,[^65^, ^66^] the reversibility of the substitution is completely unexplored. In this regard, phenols or thiols may act both as good nucleophiles and as efficient leaving groups, and therefore it seems reasonable to focus on them as optimal candidates as substituents of tetrazine to achieve a reversible S_N_Ar.

**Figure 1:**
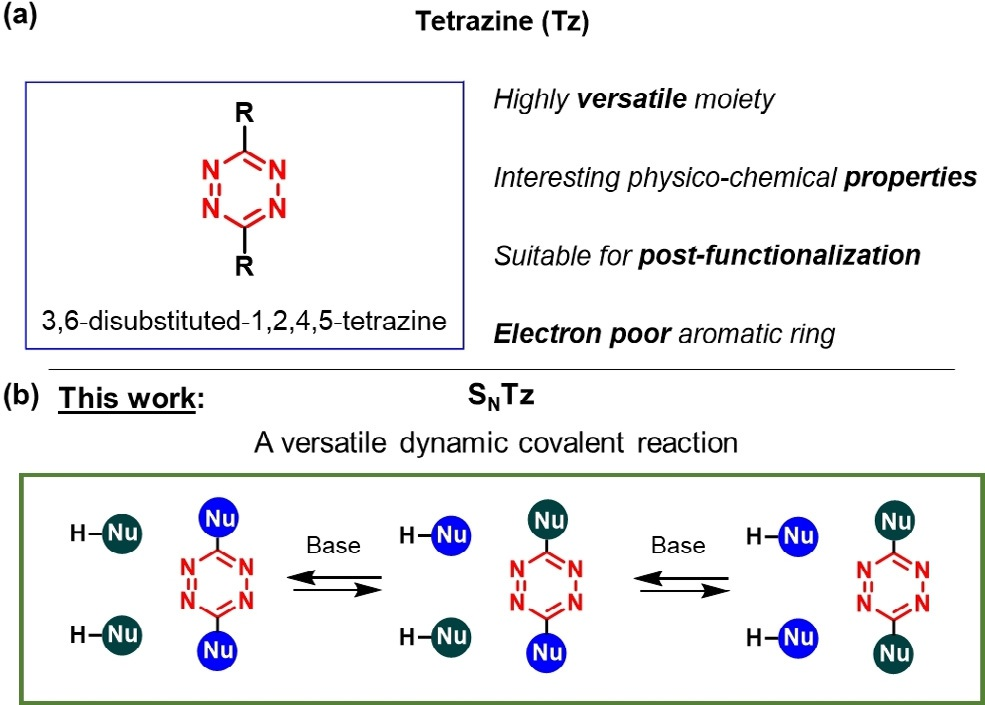
a) Tetrazine is a very versatile moiety with interesting properties. b) This work combines the intrinsic advantages of dynamic covalent reactions with the chemical versatility of the tetrazine ring.

Having all that in mind, a novel tool for DCC is herein introduced: The nucleophilic aromatic substitution of tetrazines (S_N_Tz) with phenols and alkyl thiols (Figure 1). This fast exchange reaction combines the intrinsic advantages of dynamic covalent chemistry with the chemical versatility of the tetrazine moiety. In fact, it allows not only to obtain dynamic libraries of compounds, or complex molecular structures in an easier way, but it also takes advantage of the tetrazine moiety itself, and its interesting physico‐chemical properties.

The dynamic nature of S_N_Tz has been successfully confirmed by obtaining the same distribution of compounds in a series of reactions conducted in both forward and reverse directions. Activation and deactivation of the exchange was observed by the presence or absence of a base, respectively. Additionally, other exciting features of this dynamic reaction were also proved: Post‐functionalization by IEDDA, which also inhibits the exchange process; photolysis by UV irradiation; and finally this exchange reaction successfully proved its potential in some supramolecular systems. Indeed, light induced guest‐release and responsive covalent cages were also achieved by S_N_Tz.

## Results and Discussion

**Figure 2:**
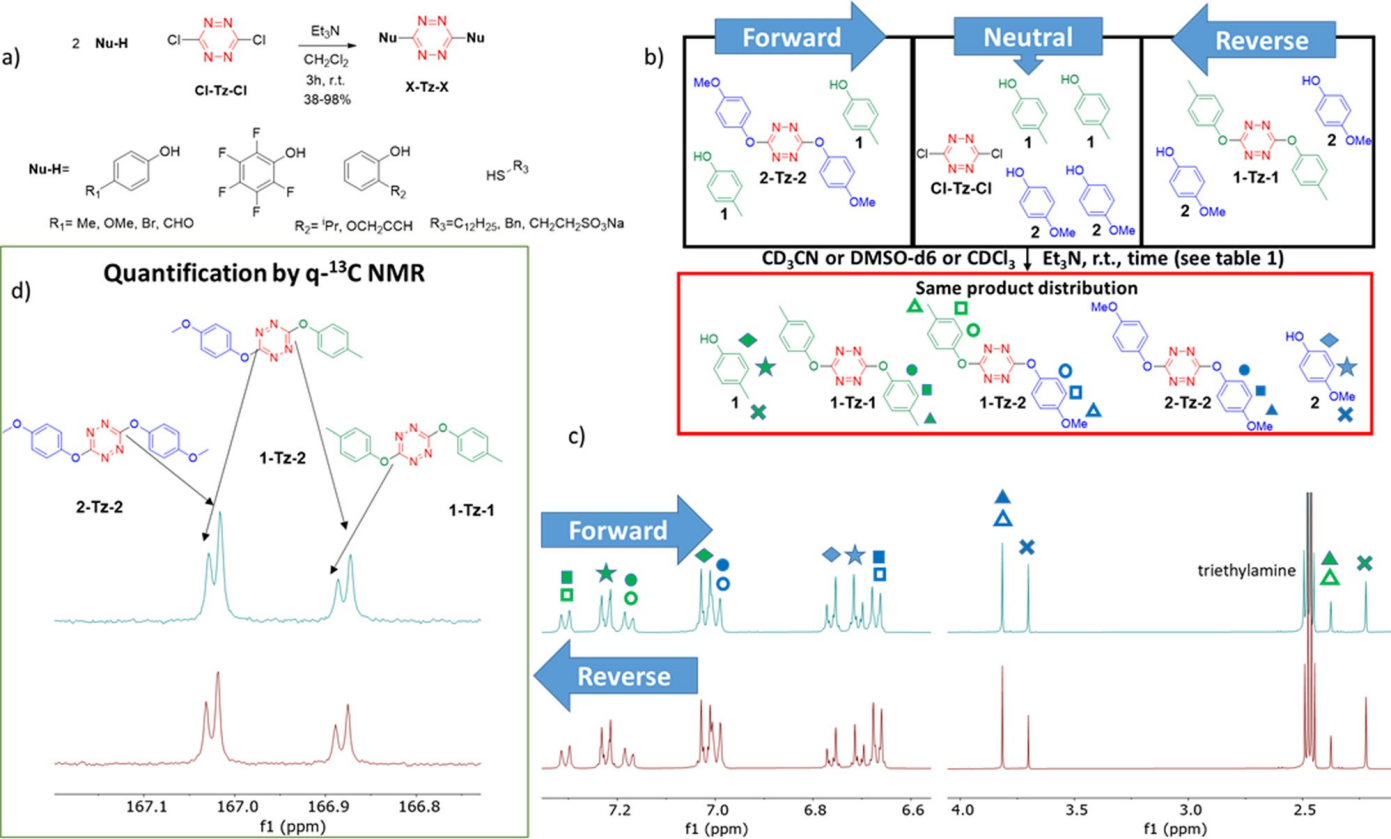
a) Synthesis of the disubstituted tetrazines was carried out from **Cl‐Tz‐Cl** and the corresponding thiol or phenol. b) Reactions performed on both directions (forward and reverse) and also starting directly from **Cl‐Tz‐Cl** (neutral), led to identical product distribution. c) ^1^H NMR of the forward and reverse reactions in CD_3_CN. Unfortunately the peaks of **1‐Tz‐2** and those of the homodimers overlap, so no quantification of all the compounds of the reaction mixture can be performed by ^1^H NMR. d) The ^13^C NMR shows slight differences between homo and hetero dimers of tetrazine, and therefore a quantitative experiment can be carried out to obtain the ratios of all the compounds in equilibrium.

3,6‐di‐substituted tetrazines were prepared starting from the corresponding nucleophile and 3,6‐dichloro‐1,2,4,5‐tetrazine (**Cl‐Tz‐Cl**) (Figure 2 a), which can be synthesized in five steps in gram scale.^[67]^ Amines and aliphatic alcohols were discarded from this work because previous studies,[^39^, ^66^, ^68^] and several experiments in our lab showed that di‐substituted tetrazines are frequently hard to obtain with that kind of nucleophiles: Harsh conditions are required and usually only low yields are obtained. On the other hand both phenols and thiols give good yields of di‐substituted tetrazines at room temperature and thus we focused on them for this work.^[67]^

We took the reaction between cresol (**1**) and the disubstituted tetrazine with 4‐methoxyphenol (**2‐Tz‐2**) as the reference process, which was compared with the reverse reaction, between 4‐methoxyphenol (**2**) and the cresol disubstituted tetrazine (**1‐Tz‐1**), to test reversibility and exchange ability in S_N_Tz (Figure 2 b). One equivalent of the disubstituted tetrazine and two equivalents of the corresponding phenol were solved in different deuterated solvents, and finally 3 equivalents of triethylamine were added. Fortunately, the experimentally indistinguishable NMR spectra obtained for reactions conducted in both forward and reverse directions demonstrate that equilibrium was achieved (Figure 2 c and d). Interestingly, when reaction was set up directly from **Cl‐Tz‐Cl** and two equivalents of phenols **1** and **2** and triethylamine in acetonitrile (neutral in Figure 2 b), exactly the same distribution of products than the forward and reverse reaction was found.^[67]^

As expected, polar aprotic solvents accelerate the reaction (Table 1). Indeed, in CDCl_3_ reaction was very slow at room temperature, although progression was evident at 50 °C, and after 15 hours at that temperature, equilibrium was reached. On the other hand, in deuterated DMSO at room temperature, reaction equilibrated so fast that it was impossible to be followed by NMR, while in acetonitrile it was complete in less than two hours at room temperature or in 30 minutes heating at 50 °C. The choice of the base also affects the kinetics of the exchange. Triethylamine (TEA) and di‐isopropylethylamine (DIPEA) yield the fastest exchange, while less basic compounds such as N‐methylmorpholine (NMM) take longer times to completion, and pyridine was not even able to induce the exchange. Inorganic bases such as Cs_2_CO_3_ in DMSO were detrimental to the reaction.^[69]^

**Table 1:** Equilibration time observed for the reverse reference reaction. 
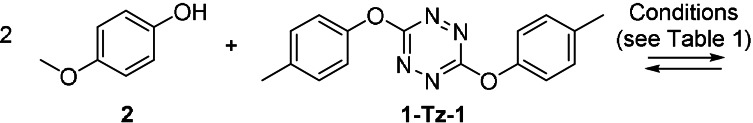

Solvent	Base	*t* [min]
CDCl_3_	TEA	1000^[a]^
CD_3_CN	TEA	90 (30)^[a]^
[D_6_]DMSO	TEA	<5^[b]^
[D_6_]DMSO	DIPEA	<5
[D_6_]DMSO	NMM	600
[D_6_]DMSO	Pyridine	n.r.^[c]^
[D_6_]DMSO	Cs_2_CO_3_	–^[d]^

[a] Heating at 50 °C. [b] Equilibrium reached already in the first NMR spectrum acquired. [c] No progress of the reaction was observed after 5 days. [d] Decomposition of the reagents.

It was also shown that a base is required for a successful exchange of phenols. In fact, when trifluoroacetic acid was added after 10 minutes to the reaction in acetonitrile, the progress of the reaction was stopped. After additional 10 minutes, a new addition of triethylamine allows to reinitiate the exchange to finally reach equilibrium. Therefore, S_N_Tz can be stopped and activated by the proper addition of an acid or a base, respectively, and reminds in this sense to the imine exchange, but with the opposite Scheme of activation/deactivation.^[67]^

Obviously, an important part of this work was to quantify all the species coexisting in the equilibrium. However, in the ^1^H NMR spectra, the peaks corresponding to the heterodimer **1‐Tz‐2** overlap with those of the homodimers **1‐Tz‐1** and **2‐Tz‐2**, and therefore it is not possible to distinguish each compound in the mixture and as a consequence, the ratio of all the species of the reaction cannot be calculated by ^1^H NMR (Figure 2 b). Conversely, some of the peaks in the ^13^C NMR spectrum can be unambiguously assigned to the heterodimer, and therefore a quantitative ^13^C NMR could be used to quantify each and every chemical species in the reaction mixture. (Figure 2 d and Table 2, entry 1).

**Table 2:** Quantification of different equilibria and their corresponding constants.

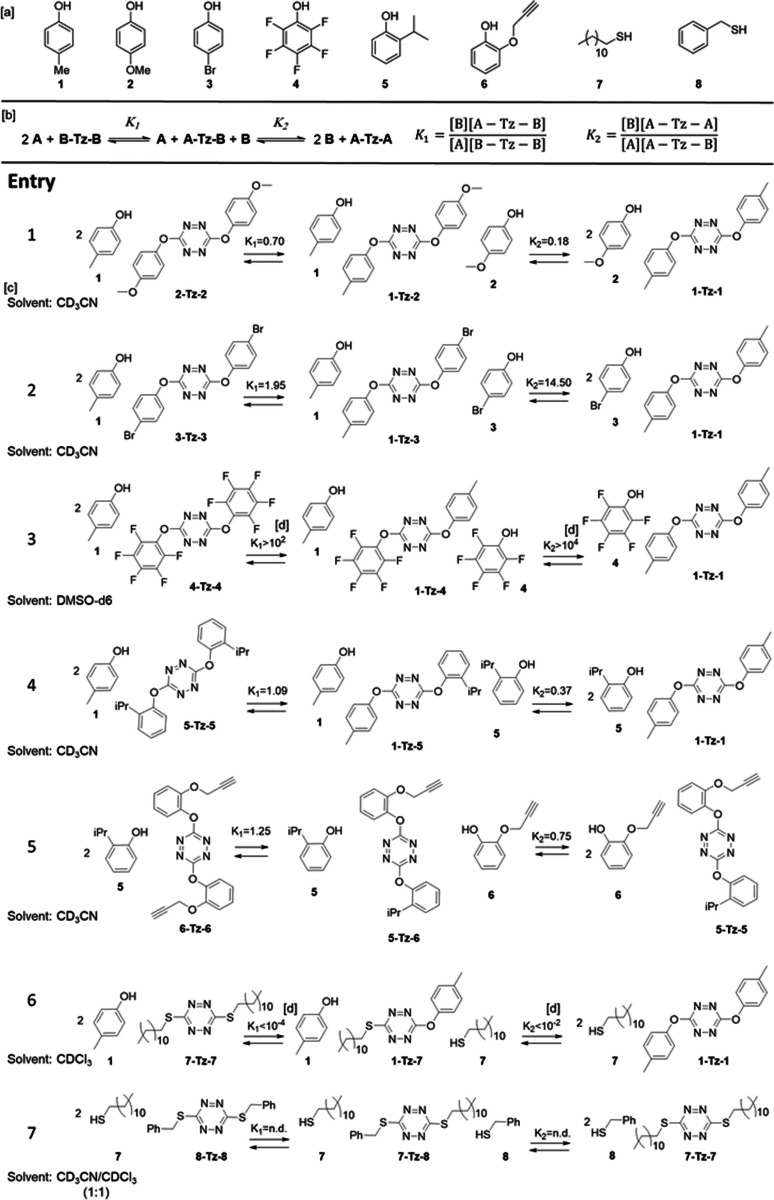

[a] Nucleophiles employed. [b] Schematic equilibria and their corresponding constants. [c] All reactions were carried out in 0.5 mL of the specified solvent (chosen owing to solubility and kinetic reasons), and 0.02 mmol of the corresponding phenol or thiol, which were adjusted to the correct stoichiometry, prior to the addition of 5 μL of triethylamine. Reactions were followed by ^1^H NMR until no further changes were observed. Equilibration times range from less than 5 minutes to 24 hours. [d] Only the peaks corresponding to the compounds displayed at one of the ends of the equilibria can be seen by ^1^H NMR, and we assumed that less than 1 % of the rest of the compounds is present in the mixture, so we could estimate a constant. n. d.: not determined.

It is clear from Table 2 (entry 1) that the equilibrium is shifted towards the release of cresol **1** while phenol **2** tends to bind the tetrazine. This result is reasonable considering the respective Hammett sigma of both substituents on the phenol, being **1** the best leaving group (*σ*_p_=−0.268 for methoxy, *σ*_p_=−0.170 for methyl group). Reversibility of this process was further proved by shifting the equilibrium backwards, i. e. towards the unfavorable direction by increasing the concentration of one of the chemical species in solution.^[70]^ Specifically, once the equilibrium was reached in the reaction between **2** and **1‐Tz‐1**, which implies that most of **2** is attached to the tetrazine ring, we added 10 more equivalents of cresol **1**, in order to force the release of phenol **2**. It is clearly seen by NMR that **2** is almost completely detached from the tetrazine ring.^[67]^

To examine the scope of this dynamic process and the effect of the nature of the nucleophile in the outcome of the equilibrium, a series of different reactions were set up in deuterated solvents at room temperature (Table 2). Both the forward and the reverse reactions were followed until no further changes were observed. Then, quantification of all the compound in the reaction mixture was performed by quantitative ^13^C NMR,^[67]^ except for the reaction with 4‐bromophenol **3** (entry 2), that could be quantified by ^1^H NMR. Moreover, as in most of the cases a complete quantification of the species in equilibrium could be achieved, then calculation of the corresponding equilibrium constants was trivial (Table 2). Curiously, there are scarce papers about dynamic covalent reactions where the equilibrium constants of the exchanges are calculated,[^71^, ^72^] even when it is a key physico‐chemical property for a reversible process and it provides a great deal of information.

From Table 2, it is clear that electron rich phenols tend to remain attached to the tetrazine ring (smaller constants) while electron poor ones are more prone to be released (larger constants) (entries 1–4). Actually, 4‐bromophenol **3** (entry 2) is mostly found free in the equilibrium, and when the extremely electron poor pentafluorophenol **4** is compared to cresol **1** (entry 3), the equilibrium is completely shifted towards the release of **4**.

It is worth mentioning that phenols with substituents in ortho position also reached the equilibrium, although much more slowly. Indeed, **5** and **6** only equilibrated after 24 hours in acetonitrile (entry 5). Thiols on the other hand might be problematic in this reaction. Many of them, such as N‐acetyl‐L‐cysteine, 2‐mercaptoethanol or all the thiophenols tested led to a competitive redox process: Reduction of the tetrazine ring to 1,4‐dihydro‐*s*‐tetrazine, with the concomitant oxidation of the thiol to the corresponding disulfide.[^67^, ^73^] Only some aliphatic thiols such as dodecanethiol and benzylmercaptan display an efficient exchange reaching equilibrium so fast that it cannot be followed by NMR (entry 7). Interestingly, alkyl thiols tend to displace phenols from the tetrazine very efficiently. Indeed dodecanethiol **7** completely displaces cresol from the tetrazine in 5 hours (entry 6),^[67]^ while in the other direction of the reaction, cresol **1** is not able to substitute the thiol attached to the tetrazine core.

**Figure 3:**
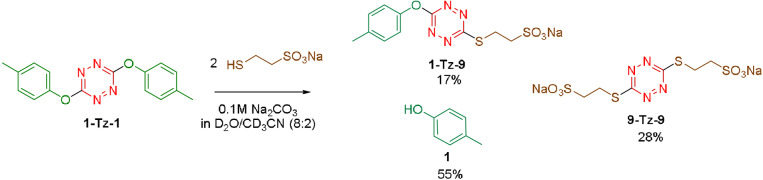
S_N_Tz in aqueous environments.

Dynamic covalent chemistry in aqueous environments is challenging for some reactions such as imines and boronic esters exchanges due to their labile nature.[^74^, ^75^, ^76^] However the bonds involved in S_N_Tz are more robust. Thus we tested one exchange between **1‐Tz‐1** and sodium 2‐mercaptoethane‐sulfonate **9** in a mixture of deuterated water and acetonitrile (8:2). We found that 0.1 M sodium carbonate is enough to promote a successful exchange (Figure 3).

The initial suspension of **1‐Tz‐1** slowly reacts, and while the mixture homogenizes, the solution progressively gets colored. The final result is a mixture of water‐soluble compounds: cresol **1**, **9‐Tz‐9** and **1‐Tz‐9**, which are stable for days in those conditions.

**Figure 4:**
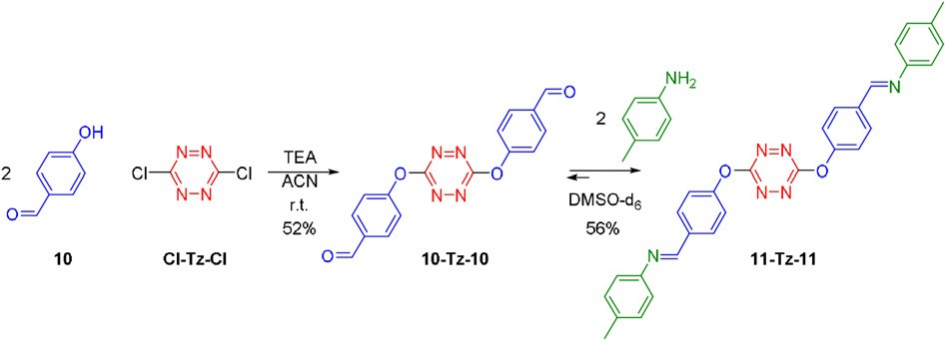
Combination of S_N_Tz and imine chemistry.

Considering the basic conditions required for S_N_Tz, we thought that it might be possible to combine it with imine chemistry that it is usually performed under acidic conditions. Combination of two or more dynamic covalent reactions in an orthogonal way is increasingly interesting because it eases the synthesis of multifunctional molecular architectures.[^77^, ^78^, ^79^, ^80^, ^81^, ^82^] Consequently, dialdehyde **10‐Tz‐10** was synthesized and exposed to toluidine in acetonitrile (Figure 4). The corresponding imine precipitated from the reaction mixture, it was filtrated and solved in deuterated DMSO. The ^1^H NMR clearly shows the formation of **11‐Tz‐11** and therefore confirms that S_N_Tz is compatible with the imine chemistry of anilines.

**Figure 5:**
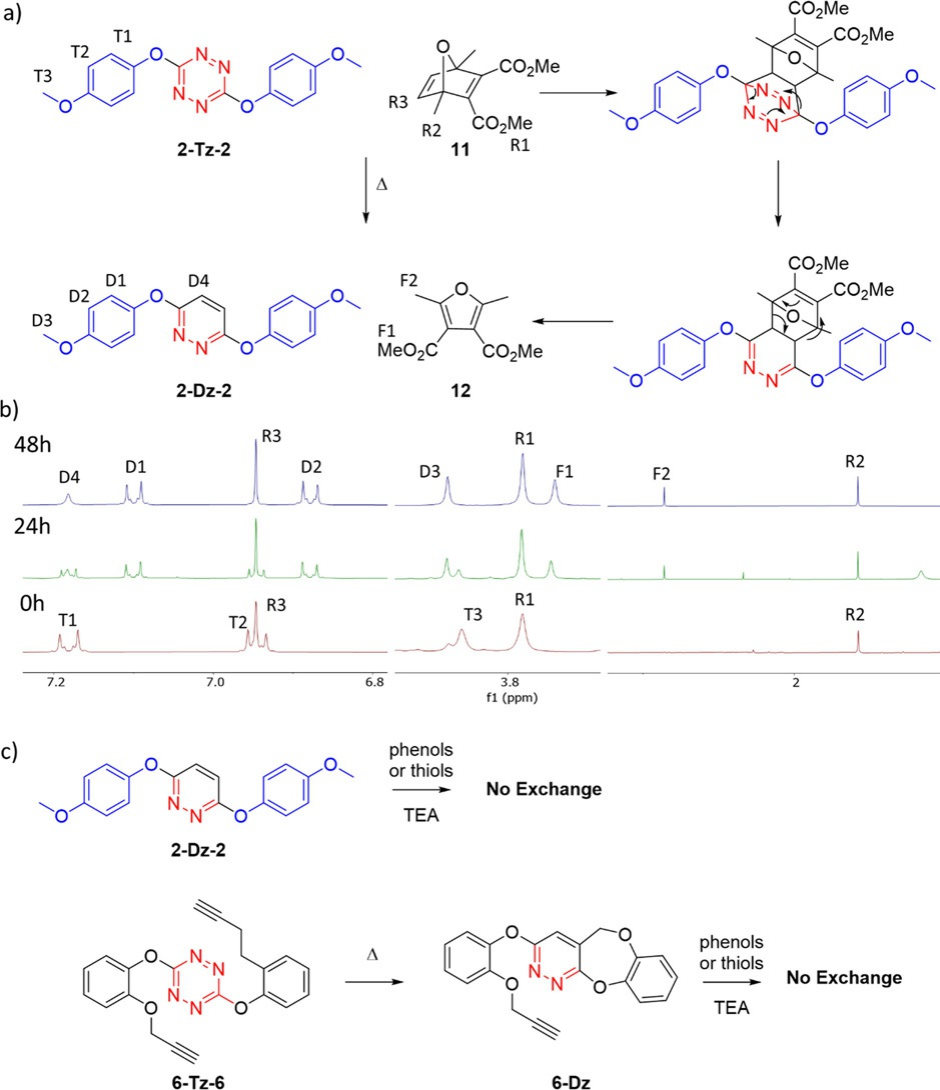
a) Inverse electron demand Diels–Alder (IEDDA) reaction on **2‐Tz‐2** with bicyclic dienophile **12**. b) ^1^H NMR of the reaction mixture before and after heating for 24 h and 48 h. c) Diazines do not undergo S_N_Tz. d) Heating converts the exchangeable **6‐Tz‐6** into unreactive **6‐Dz** by an intramolecular IEDDA reaction.

Obviously, one of the strengths of this novel dynamic reaction is the ability of post‐functionalization by inverse electron demand Diels—Alder (IEDDA). Additionally, it is interesting to check if, once the Diels–Alder reaction transforms the tetrazine into an electron‐richer 1,2‐diazine, the exchange is interrupted. Thus, the IEDDA reaction between **2‐Tz‐2** and a slight excess of bicyclic compound **12** was examined (Figure 5 a). We thought that **12** was an optimal dienophile to follow the reaction by NMR, because it displays only three singlet peaks, and once the initial cycloaddition finishes and one molecule of N_2_ is released, then a retro Diels–Alder is expected to occur, giving the diazine **2‐Dz‐2** and the furan **13** which displays just two singlets in the ^1^H NMR. Fortunately, IEDDA reaction was carried out in high yield as it can be told by the stacked NMR spectra displayed in Figure 5 b.

After 48 hours heating at 50 °C in CDCl_3_ the ^1^H NMR only showed peaks corresponding to the expected final products and the remaining **12**. The isolated yield of such post‐functionalization was 94 %. Interestingly the exchange reaction was inhibited after the functionalization (Figure 5 c). Indeed, heating **2‐Dz‐2**, and triethylamine in the presence of cresol **1** or dodecanethiol **7** led to no reaction at all, even after 5 days, neither in DMSO at room temperature, nor heating at 50 °C in CDCl_3_. Therefore, post‐functionalization by IEDDA is not only an efficient way to attach different molecules to the dynamic libraries of compounds obtained, but it is also a complementary way to cancel the exchange. Interestingly, when an intramolecular IEDDA reaction is possible, such as in compound **6‐Tz‐6**, then heating the mixture gives the corresponding diazine (**6‐Dz**) in 96 % yield, which concomitantly implies that an irreversible self‐inhibition of the exchange can be triggered by high temperatures. This property might be used for the kinetic control of the dynamic libraries of compounds obtained.[^83^, ^84^, ^85^, ^86^, ^87^, ^88^]

**Figure 6:**
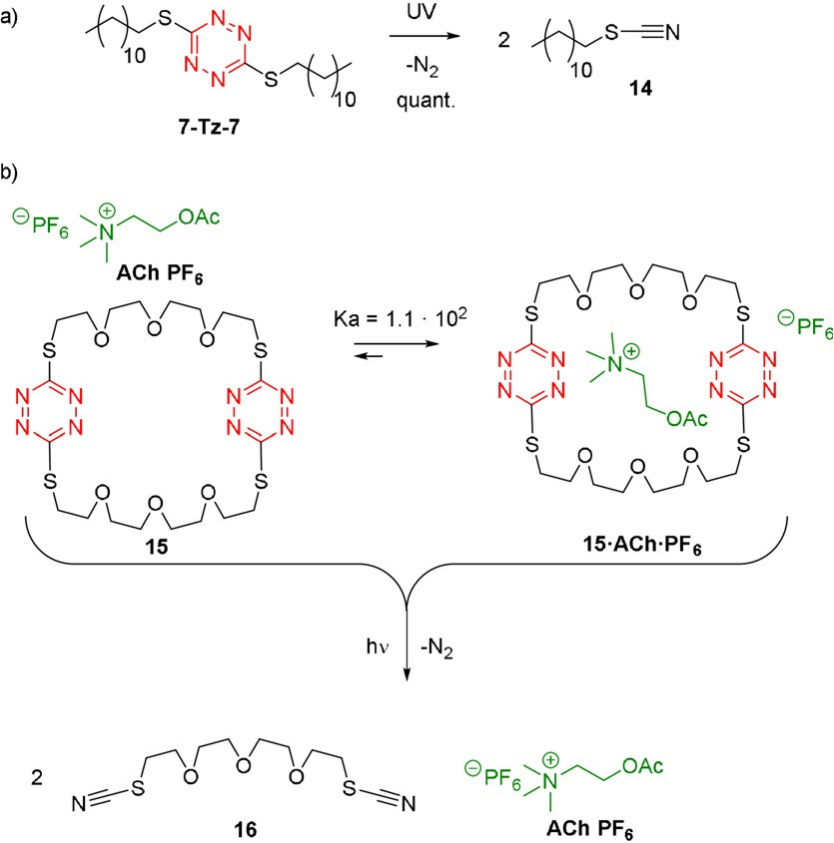
a) Photolysis of **7‐Tz‐7** by UV irradiation. b) Release of acetylcholine after macrocycle **15** is photo‐cleaved.

There are alternative ways to manipulate some of the tetrazine compounds obtained by S_N_Tz. Inspired by the work by A. B. Smith III and R. M. Hochstrasser,[^89^, ^90^] it is possible to break down the S,S‐tetrazine derivatives by photolysis which gives two molecules of the corresponding thiocyanate and one molecule of nitrogen. Indeed, by UV irradiation with a medium pressure Hg lamp (450W), complete transformation of **7‐Tz‐7** into n‐dodecyl thiocyanate **14** was achieved in two hours (Figure 6 a). It is worth mentioning, that an O,O‐tetrazine such as **1‐Tz‐1** or an O,S‐tetrazines such as **1‐Tz‐7** are not degraded by UV light. Therefore, all the S,S‐tetrazine derivatives obtained by S_N_Tz are potentially photo‐cleavable.

Controlling the behavior of molecular structures is essential to develop functional systems. As it has been proved, S_N_Tz allows for a precise chemical control of the compounds obtained, either by photodegradation under UV light or by the complete substitution of phenols by thiols. Those properties together with its dynamic nature make S_N_Tz a potentially valuable tool for the synthesis of functional systems. With all this in mind, we decided to test some of its possible applications through two examples. On one hand, we decided to prove that S_N_Tz allows for the easy synthesis of photo cleavable receptors. Controlled release of guest by light is very attractive because it is a clean and switchable process which has been reported in several supramolecular systems.[^91^, ^92^, ^93^, ^94^, ^95^]

Owing to the abovementioned intrinsical properties of S,S‐tetrazines, they could be employed to create receptors with the ability to release their guest after disassembly triggered by light. Actually, tetrazine‐bearing crown ether **15** was synthesized in two steps in 44 % yield, and it displays an association constant of 1.1×10^2^ M^−1^ with neurotransmitter acetylcholine hexafluorophosphate (ACh PF_6_) in acetonitrile (Figure 6 b).[^96^, ^97^, ^98^, ^99^] However, UV irradiation causes the macrocycle to break down into two molecules of dithiocyanate podand **16**, which shows no binding with acetylcholine. Therefore, S_N_Tz is not only an efficient way to click molecular fragments into a functional structure, but it confers macrocycle **15** the ability to release acetylcholine triggered by UV irradiation, although it seems difficult to apply this results to biological systems for the time being, considering the potent irradiation required for hours for a successful release.

**Figure 7:**
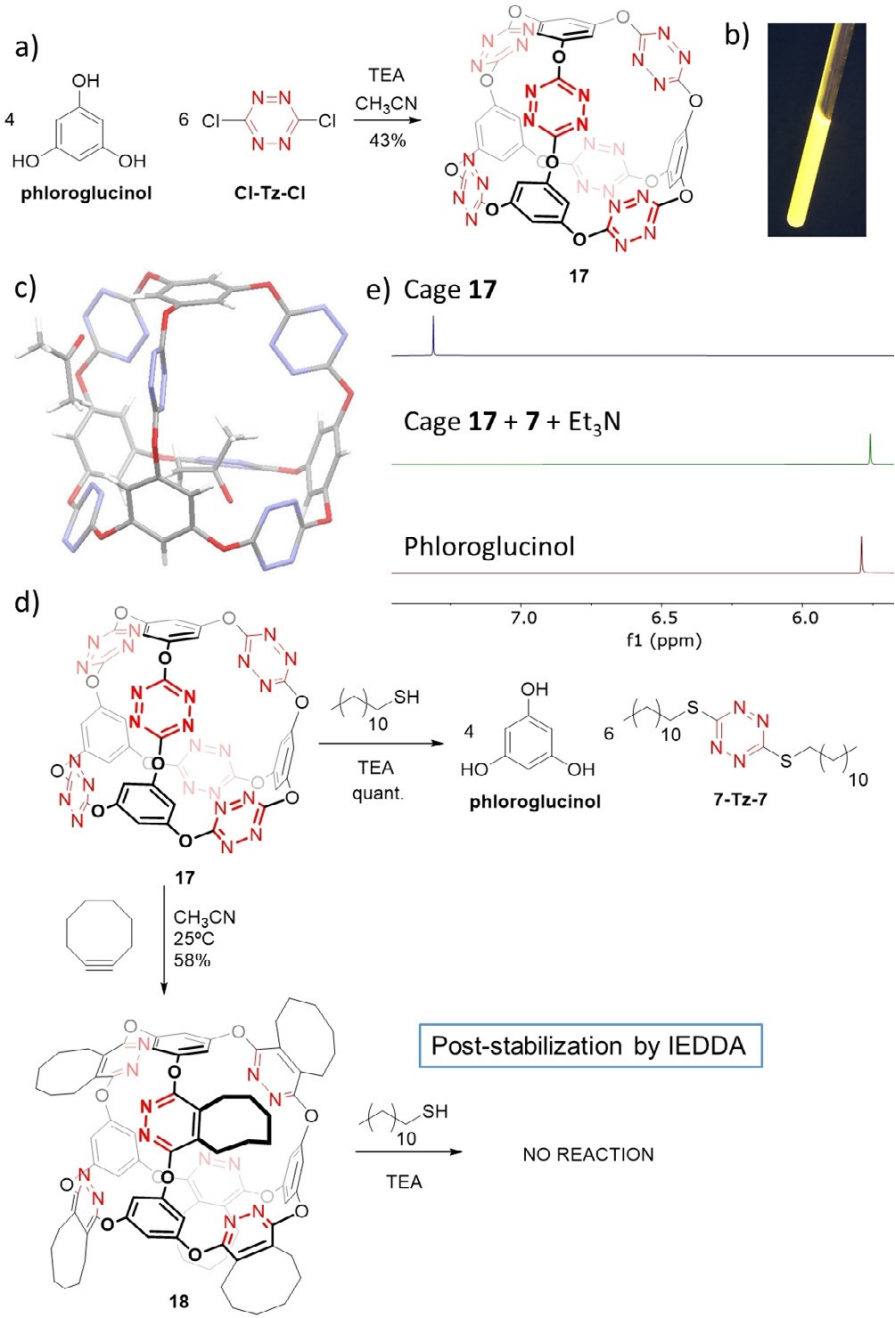
a) Synthesis of cage **17**. b) Yellow–orange fluorescence of cage **17** under UV light. c) Single‐crystal X‐ray molecular structure of **17**.^[118]^ One molecule of acetone lies inside the cavity while other is outside. d) Cage **17** is disassembled by thiol **7** in the presence of triethylamine, however post‐stabilization can be achieved by IEDDA. e) Stacked plot of the ^1^H NMR of pure **17**, the crude of the disassembling reaction mixture after 30 minutes and phloroglucinol all in CD_3_CN.

On the other hand, S_N_Tz allows clicking phenols together and declicking them with thiols.^[100]^ Such ability could be used to synthesize responsive molecular architectures, that would be disassembled upon the presence of the right chemical environment. We decided to test it on a shape persistent cage, as it is one of the traditional uses of dynamic methodologies.[^27^, ^101^, ^102^, ^103^, ^104^, ^105^, ^106^, ^107^, ^108^, ^109^, ^110^] We reasoned that phloroglucinol and **Cl‐Tz‐Cl** should lead to a truncated [4+6] tetrahedral cage, where each of the four faces is actually an i‐corona[6]arene (Figure 7 a).^[111]^ As expected, cage **17** was successfully synthesized in one step in 43 % yield.

This covalent molecular capsule displays an orange fluorescence with a maximum emission wavelength in acetone of 532 nm (Figure 7 b). A suitable single crystal was obtained by slow diffusion of hexane into acetone/dichloromethane (1:1). The compound **17** crystallized in the non‐centrosymmetric *Pna*2_1_ space group and a single cage and two acetone solvent molecules form its asymmetric unit.^[67]^ Each tetrahedral cage is formed by four phloroglucinol groups located in the vertices and six tetrazine groups on the edges. They form an almost perfect tetrahedron able to accommodate a sphere of 7 Å of diameter inside.^[67]^

In spite of the reported good anion binding abilities of i‐corona[6]arenes,^[111]^ cage **17** shows no remarkable association constant with any anion tested. This fact is probably due to rigidity of the cage compared with i‐corona[6]arenes: In the macrocycle the aromatic proton can be accommodated towards the cavity and plays a key role on the binding event, which is obviously not possible with the cage.

Interestingly, **17** is able to respond to a chemical stimulus such as the presence of a thiol. Indeed dodecanethiol **7** in the presence of triethylamine is able to rapidly disassemble the cage into phloroglucinol and **7‐Tz‐7** (Figure 7 d). Such reaction can be followed by ^1^H NMR where the singlet peak of cage **17** disappears completely in less than 30 minutes, and an up field singlet corresponding to phloroglucinol shows up (Figure 7 e). Notably, the fluorescence of the reaction mixture is lost as none of the final products is fluorescent. As expected, once cage **17** is conveniently functionalized by IEDDA, the resulting diazine cage **18** becomes unresponsive to the effect of thiols. To the best of our knowledge, there are only a couple of precedents of such post‐stabilization by Diels–Alder reaction and in both cases, only for DCC products based on imines.[^112^, ^113^]

## Conclusion

We have shown that nucleophilic substitution of tetrazines (S_N_Tz) with phenols and alkyl thiols is a truly dynamic covalent reaction. The examples reported herein may serve as glimpse of the numerous applications that S_N_Tz can find. All those possibilities are related not only to the dynamic nature of the process itself, but also to the inherent chemical properties of the tetrazine. Indeed, it has been shown that post‐functionalization by IEDDA of the compounds obtained allows for the attachment of molecular fragments while at the same time it inhibits the exchange. Moreover the thiol derivatives can be photolized by UV irradiation as an efficient and clean way to break down irreversibly those compounds. Functional supramolecular systems such as a tetrahedral responsive fluorescent cage and a macrocycle that releases acetylcholine upon irradiation were also studied.

S_N_Tz is a robust and versatile dynamic covalent reaction an therefore it unleashes a huge potential of applications in the synthesis of porous organic cages, interlocked structures, covalent adaptable networks and COFs, all of which could be post‐functionalized, and, in some cases they could also respond to the right stimulus, either UV irradiation or a specific chemical reagent. Moreover, modulation of the compounds obtained through IEDDA or many other chemical reactions,[^114^, ^115^, ^116^, ^117^] will ease the access to numerous functional systems. Other well‐known properties of tetrazines such as fluorescence or an interesting redox behavior, foresee also a bright future to S_N_Tz in the fields of molecular sensors and electronics. There are undoubtedly plenty of possibilities for this novel dynamic covalent reaction awaiting to be explored.

## Conflict of interest

The authors declare no conflict of interest.

## Supporting information

As a service to our authors and readers, this journal provides supporting information supplied by the authors. Such materials are peer reviewed and may be re‐organized for online delivery, but are not copy‐edited or typeset. Technical support issues arising from supporting information (other than missing files) should be addressed to the authors.

Supporting Information

Supporting Information
