# Supplementary material for: Dynamic Nucleophilic Aromatic Substitution of Tetrazines
Source: Angew Chem Int Ed Engl. 2021 Jul 12;60(34):18783–91. doi: 10.1002/anie.202106230 (PMC8457238; doi:10.1002/anie.202106230)

checkCIF/PLATON report

Structure factors have been supplied for datablock(s) compound_1

THIS REPORT IS FOR GUIDANCE ONLY. IF USED AS PART OF A REVIEW PROCEDURE FOR PUBLICATION, IT SHOULD NOT REPLACE THE EXPERTISE OF AN EXPERIENCED CRYSTALLOGRAPHIC REFEREE.

No syntax errors found. CIF dictionary Interpreting this report

Datablock: compound_1

Bond precision:	C-C = 0.0132 A	Wavelength=0.71073	
Cell:	a=27.0686(15)	b=11.0085(5)	c=16.2348(8)
	alpha=90	beta=90	gamma=90
Temperature:	293 K		

	Calculated	Reported
Volume	4837.7(4)	4837.8(4)
Space group	P n a 21	P n a 21
Hall group	P 2c -2n	P 2c -2n
Moiety formula	C36 H12 N24 O12, 2(C3 H6 O)	C36 H12 N24 O12, 2(C3 H6 O)
Sum formula	C42 H24 N24 O14	C42 H24 N24 O14
Mr	1088.85	1088.85
Dx,g cm-3	1.495	1.495
Z	4	4
Mu (mm-1)	0.118	0.118
F000	2224.0	2224.0
F000’	2225.04	
h,k,lmax	36,14,21	36,14,21
Nref	12186[6303]	11057
Tmin,Tmax	0.990,0.994	0.775,1.000
Tmin’	0.889	

Correction method= # Reported T Limits: Tmin=0.775 Tmax=1.000
AbsCorr = GAUSSIAN

Data completeness= 1.75/0.91 Theta(max)= 28.436

R(reflections)= 0.0804(5696) wR2(reflections)= 0.1687(11057)

S = 1.102 Npar= 726

The following ALERTS were generated. Each ALERT has the format
       test-name_ALERT_alert-type_alert-level.
Click on the hyperlinks for more details of the test.

Alert level B

```
PLAT340_ALERT_3_B Low Bond Precision on  C-C Bonds ...............    0.01318 Ang.
```

Alert level C

```
STRVA01_ALERT_4_C           Flack test results are meaningless.
           From the CIF: _refine_ls_abs_structure_Flack    0.000
           From the CIF: _refine_ls_abs_structure_Flack_su    2.000
PLAT213_ALERT_2_C Atom N17             has ADP max/min Ratio .....        3.1 prolat
PLAT234_ALERT_4_C Large Hirshfeld Difference C21      --C22      .       0.17 Ang.
PLAT234_ALERT_4_C Large Hirshfeld Difference C29      --C30      .       0.17 Ang.
PLAT241_ALERT_2_C High   'MainMol' Ueq as Compared to Neighbors of         O1 Check
PLAT241_ALERT_2_C High   'MainMol' Ueq as Compared to Neighbors of         O2 Check
PLAT241_ALERT_2_C High   'MainMol' Ueq as Compared to Neighbors of         O3 Check
PLAT241_ALERT_2_C High   'MainMol' Ueq as Compared to Neighbors of         O4 Check
PLAT241_ALERT_2_C High   'MainMol' Ueq as Compared to Neighbors of         O5 Check
PLAT241_ALERT_2_C High   'MainMol' Ueq as Compared to Neighbors of         O6 Check
PLAT241_ALERT_2_C High   'MainMol' Ueq as Compared to Neighbors of         O7 Check
PLAT241_ALERT_2_C High   'MainMol' Ueq as Compared to Neighbors of         O8 Check
PLAT241_ALERT_2_C High   'MainMol' Ueq as Compared to Neighbors of        O10 Check
PLAT241_ALERT_2_C High   'MainMol' Ueq as Compared to Neighbors of        O11 Check
PLAT241_ALERT_2_C High   'MainMol' Ueq as Compared to Neighbors of        O12 Check
PLAT241_ALERT_2_C High   'MainMol' Ueq as Compared to Neighbors of         N5 Check
PLAT241_ALERT_2_C High   'MainMol' Ueq as Compared to Neighbors of         N6 Check
PLAT241_ALERT_2_C High   'MainMol' Ueq as Compared to Neighbors of        N17 Check
PLAT241_ALERT_2_C High   'MainMol' Ueq as Compared to Neighbors of        N18 Check
PLAT242_ALERT_2_C Low    'MainMol' Ueq as Compared to Neighbors of         C3 Check
PLAT242_ALERT_2_C Low    'MainMol' Ueq as Compared to Neighbors of         C7 Check
PLAT242_ALERT_2_C Low    'MainMol' Ueq as Compared to Neighbors of         C8 Check
PLAT242_ALERT_2_C Low    'MainMol' Ueq as Compared to Neighbors of        C15 Check
PLAT242_ALERT_2_C Low    'MainMol' Ueq as Compared to Neighbors of        C16 Check
PLAT242_ALERT_2_C Low    'MainMol' Ueq as Compared to Neighbors of        C31 Check
PLAT242_ALERT_2_C Low    'MainMol' Ueq as Compared to Neighbors of        C32 Check
PLAT242_ALERT_2_C Low    'MainMol' Ueq as Compared to Neighbors of        C33 Check
PLAT242_ALERT_2_C Low    'MainMol' Ueq as Compared to Neighbors of        C34 Check
PLAT244_ALERT_4_C Low    'Solvent' Ueq as Compared to Neighbors of        C38 Check
PLAT244_ALERT_4_C Low    'Solvent' Ueq as Compared to Neighbors of        C41 Check
PLAT260_ALERT_2_C Large Average Ueq of Residue Including       O13      0.186 Check
PLAT260_ALERT_2_C Large Average Ueq of Residue Including       O14      0.122 Check
PLAT334_ALERT_2_C Small Aver. Benzene C-C Dist C1       -C6              1.37 Ang.
PLAT334_ALERT_2_C Small Aver. Benzene C-C Dist C9       -C14             1.37 Ang.
PLAT334_ALERT_2_C Small Aver. Benzene C-C Dist C17      -C22             1.37 Ang.
PLAT334_ALERT_2_C Small Aver. Benzene C-C Dist C25      -C30             1.37 Ang.
PLAT362_ALERT_2_C Short  C(sp3)-C(sp2) Bond  C37      - C38      .       1.39 Ang.
PLAT906_ALERT_3_C Large K Value in the Analysis of Variance ......     15.863 Check
PLAT906_ALERT_3_C Large K Value in the Analysis of Variance ......      3.813 Check
PLAT906_ALERT_3_C Large K Value in the Analysis of Variance ......      5.363 Check
PLAT906_ALERT_3_C Large K Value in the Analysis of Variance ......      2.994 Check
PLAT906_ALERT_3_C Large K Value in the Analysis of Variance ......      2.879 Check
```

Alert level G

```
PLAT032_ALERT_4_G Std. Uncertainty on Flack Parameter Value High .      2.000 Report
PLAT063_ALERT_4_G Crystal Size Possibly too Large for Beam Size ..       1.00 mm
PLAT199_ALERT_1_G Reported _cell_measurement_temperature ..... (K)        293 Check
PLAT200_ALERT_1_G Reported   _diffrn_ambient_temperature ..... (K)        293 Check
```

```
PLAT432_ALERT_2_G Short Inter X...Y Contact  O13      ..C8              2.98 Ang.
                                                      x,y,z  =      1_555 Check
PLAT432_ALERT_2_G Short Inter X...Y Contact  O14      ..C24             2.90 Ang.
                                                      x,y,z  =      1_555 Check
PLAT850_ALERT_4_G Check Flack Parameter Exact Value 0.00 with s.u.      2.00 Check
PLAT870_ALERT_4_G ALERTS Related to Twinning Effects Suppressed ..         ! Info
PLAT883_ALERT_1_G No Info/Value for _atom_sites_solution_primary .    Please Do !
PLAT910_ALERT_3_G Missing # of FCF Reflection(s) Below Theta(Min).         1 Note
PLAT912_ALERT_4_G Missing # of FCF Reflections Above STh/L=  0.600       374 Note
PLAT916_ALERT_2_G Hooft y and Flack x Parameter Values Differ by .      0.60 Check
PLAT933_ALERT_2_G Number of OMIT Records in Embedded .res File ...         1 Note
```

```
   0 ALERT level A = Most likely a serious problem - resolve or explain
   1 ALERT level B = A potentially serious problem, consider carefully
  42 ALERT level C = Check. Ensure it is not caused by an omission or oversight
  13 ALERT level G = General information/check it is not something unexpected

   3 ALERT type 1 CIF construction/syntax error, inconsistent or missing data
  36 ALERT type 2 Indicator that the structure model may be wrong or deficient
   7 ALERT type 3 Indicator that the structure quality may be low
  10 ALERT type 4 Improvement, methodology, query or suggestion
   0 ALERT type 5 Informative message, check
```

It is advisable to attempt to resolve as many as possible of the alerts in all categories. Often the minor alerts point to easily fixed oversights, errors and omissions in your CIF or refinement strategy, so attention to these fine details can be worthwhile. In order to resolve some of the more serious problems it may be necessary to carry out additional measurements or structure refinements. However, the purpose of your study may justify the reported deviations and the more serious of these should normally be commented upon in the discussion or experimental section of a paper or in the "special_details" fields of the CIF. checkCIF was carefully designed to identify outliers and unusual parameters, but every test has its limitations and alerts that are not important in a particular case may appear. Conversely, the absence of alerts does not guarantee there are no aspects of the results needing attention. It is up to the individual to critically assess their own results and, if necessary, seek expert advice.

Publication of your CIF in IUCr journals

A basic structural check has been run on your CIF. These basic checks will be run on all CIFs submitted for publication in IUCr journals (*Acta Crystallographica*, *Journal of Applied Crystallography*, *Journal of Synchrotron Radiation*); however, if you intend to submit to *Acta Crystallographica Section C* or *E* or *IUCrData*, you should make sure that full publication checks are run on the final version of your CIF prior to submission.

Publication of your CIF in other journals

Please refer to the *Notes for Authors* of the relevant journal for any special instructions relating to CIF submission.

PLATON version of 05/12/2020; check.def file version of 05/12/2020

Datablock compound_1 - ellipsoid plot

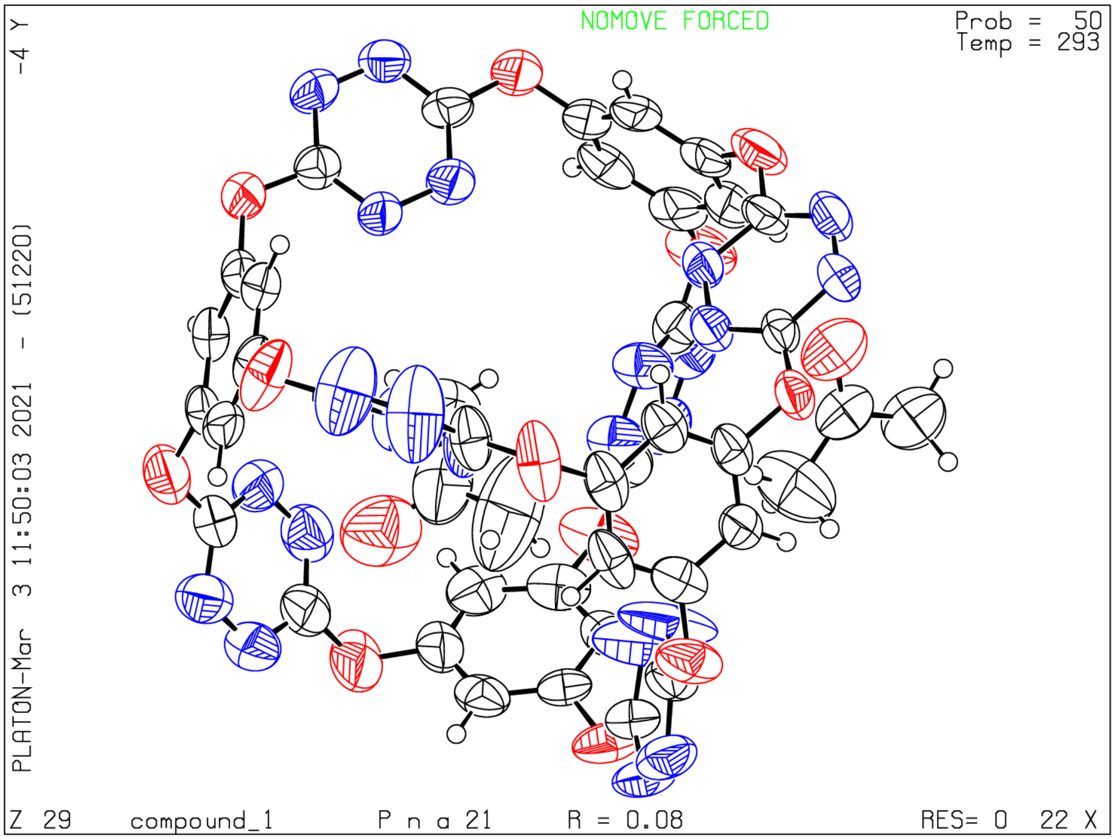

Supplement: Supplementary file 1 — Supporting Information [file ANIE-60-18783-s001.pdf]
