# Supplementary material for: Dynamic Nucleophilic Aromatic Substitution of Tetrazines
Source: Angew Chem Int Ed Engl. 2021 Jul 12;60(34):18783–91. doi: 10.1002/anie.202106230 (PMC8457238; doi:10.1002/anie.202106230)
Supplement: Supplementary file 2 — Supporting Information [file ANIE-60-18783-s002.pdf]

Angewandte Chemie
Eine Zeitschrift der Gesellschaft Deutscher Chemiker

Supporting Information

Dynamic Nucleophilic Aromatic Substitution of Tetrazines

*Tanausú Santos, David S. Rivero, Yaiza Pérez-Pérez, Endika Martín-Encinas, Jorge Pasán, Antonio Hernández Daranas, and Romen Carrillo**

anie_202106230_sm_miscellaneous_information.pdf

SUPPORTING INFORMATION

Table of Contents

1. Materials and methods S2
2. Synthesis of 3,6-dichloro-1,2,4,5-tetrazine S2
3. Synthesis of O, O-tetrazine derivatives. S5
4. Synthesis of S, S-tetrazine derivatives S9
5. Synthesis of O, S-tetrazine derivatives S10
6. Inverse Electron Demand Diels Alder Reactions S11
7. Synthesis of macrocycle **15** S13
8. Synthesis of cage **17** S15
9. Post-functionalization of cage **17**: Synthesis of cage **18** S15
10. Covalent Dynamic Studies: ^{1}H NMR experiments S16
11. Covalent Dynamic Studies: Quantification and Equilibrium Constants S23
12. Kinetics Studies S33
13. Shifting the Equilibrium S41
14. Run&Stop experiment procedure S42
15. Photolysis Studies S43
16. Determination of the association constant S46
17. Fluorescence spectrum of cage **17** S48
18. Competitive redox reactions with thiol S48
19. Disassembly reaction of cage **17** S55
20. Crystal Data and X-Ray Molecular Structure of **17** S56
21. NMR spectra S60
22. References S84

Materials and methods.

All reagents from commercial suppliers were used without further purification. All solvents were freshly distilled before use from appropriate drying agents. All other reagents were recrystallized or distilled when necessary. Reactions were performed under a dry nitrogen atmosphere. Analytical TLCs were performed with silica gel 60 F_{254} plates. Visualization was accomplished by UV light or vanillin with acetic and sulfuric acid in ethanol with heating. Column chromatography was carried out using silica gel 60 (230-400 mesh ASTM). ^{1}H NMR spectra were recorded at 500 MHz and 400MHz, ^{13}C NMR spectra were recorded at 126 MHz and 100 MHz. Chemical shifts were reported in units (ppm) by assigning TMS resonance in the ^{1}H NMR spectrum as 0.00 ppm (deuterated chloroform, 7.26 ppm; acetonitrile-d_3 1.94 ppm; DMSO-d_6 2.50 ppm; acetone-d_6 2.05 ppm). Data were reported as follows: chemical shift, multiplicity (s = singlet, d = doublet, t = triplet, q=quartet, dd = double doublet, ddd = double double doublet, m =multiplet and br = broad), coupling constant (*J* values) in Hz and integration. Chemical shifts for ^{13}C NMR spectra were recorded in ppm from tetramethylsilane using the central peak of $CDCl_3$ (77.14 ppm) as the internal standard. High resolution mass spectra (HRMS) was measured by ESI method with an Agilent LC-Q-TOF-MS 6520 spectrometer.

Synthesis of 3,6-dichloro-1,2,4,5-tetrazine (Cl-Tz-Cl).

3,6-dichloro-1,2,4,5-tetrazine (**Cl-Tz-Cl**) is central to this manuscript, and it was essential to access to large quantities of it. Even when it is commercially available (CAS number: 106131-61-7), it is relatively expensive, and at the same time it is easily synthesized in 5 steps in gram scale. The synthesis of 3,6-dichloro-1,2,4,5-tetrazine has been reported elsewhere. [1,2,3,4,5] Herein we have optimized some of the steps in order to increase the yield and the reproducibility.

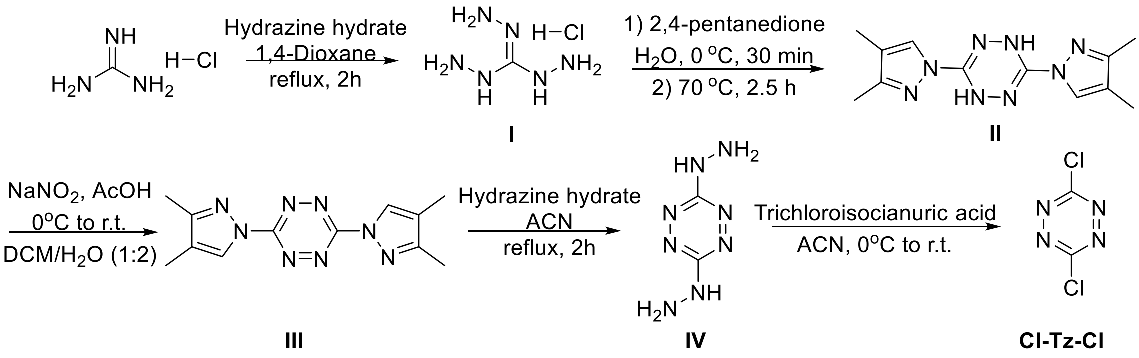

Figure S1.

Synthesis of hydrazinecarbohydrazonhydrazide **I**

To a 250 mL round bottom flask equipped with a reflux condenser and a stirrer were added guanidine hydrochloride (24.0 g, 251.2 mmol) and 1,4-dioxane (125 mL). This solvent seems to be key for a good yield, as reactions carried out in THF gave much worse results. After 5 minutes, guanidine is dissolved and hydrazine monohydrate (36.6 mL, 753.7 mmol) was added in one portion and refluxed for 2h. During this time a white precipitate is formed. The solution was cooled to room temperature and the solid was filtered and rinsed with 1,4-dioxane (2 x 50 mL). Again, it is important to use dioxane to wash the precipitate, because when THF was used, unsatisfactory results were obtained. The solid was dried under high vacuum during 24 h obtaining a white powder 35.3 g (99%). This compound has to be thoroughly dried, otherwise a slight pink coloration is perceptible and next step will not work properly. **^{13}C NMR** (126 MHz, D_2O, δ ppm): 159.5 (C). **HR-MS** (ESI$^+$, m/z): $[M]^+$ = CH_9ClN_6, calcd.: 177.0236; found 177.0242.

Synthesis of 3,6-bis(3,4-dimethyl-1H-pyrazol-1-yl)-1,4-dihydro-1,2,4,5-tetrazine **II**

To a 250 mL round bottom flask was added **I** (10 g, 71,1 mmol), a magnetic bar and water (80 mL). The white solid is dissolved at room temperature and the mixture was cooled to 0 ºC with an ice-bath. 2,4-pentanedione (14.6 mL, 142.3 mmol) was added dropwise. Progressively the solution turns yellow and a white precipitate appears. Once 2,4-pentanedione is added, the mixture is heated at 70 ºC until no more precipitation is observed. During this time the white solid is dissolved, and a yellow precipitate appears. This step should never be left for more than 2h. Longer reaction times leads to an unsuccessful reaction, because a sticky yellow solid is formed instead. Such sticky solid also appears if this step is set up with improperly dried starting material **I**. The yellow

solid is hot filtered, washed with water (3 x 50 mL) and dried under high vacuum. As a result, 8.1 g (83 %) of a yellow solid was obtained. **^{1}H NMR** (500 MHz, $CDCl_3$, δ ppm): 2.24 (s, 6H, 2 CH_3), 2.50 (s, 6H, 2 CH_3), 5.98 (s, 2H, 2 CH), 8.11 (br. s., 2H, 2 NH). **^{13}C NMR** (126 MHz, $CDCl_3$, δ ppm): 13.6 (2 CH_3), 14.0 (2 CH_3), 110.1 (2 CH), 142.7 (2 C), 145.7 (2 C), 150.1 (2 C). **HR-MS** (ESI^+, m/z): $[M+Na]^+$= $C_{12}H_{16}N_8Na$, calcd.: 295.1396; found: 295.1394.

Note: If the reaction is developed with amounts over 15 g of **I**, the amount time required to obtain the yellow solid increases while the yield decreases. Pure final product **II** is actually a white solid. The yellow color is due to an impurity which can be easily removable by column chromatography (DCM) although it does not affect further steps.

Synthesis of 3-(3,4-dimethyl-1H-pyrazol-1-yl)-6-(3,4-dimethyl-4,5-dihydro-1H-pyrazol-1-yl)-1,2,4,5-tetrazine **III**

$NaNO_2$ (8.0 g, 115.7 mmol) was placed in a 1 L dissolved round bottom flask equipped with a big magnetic stirrer and the gas outlet of the dropping funnel connected to a trap containing a 1M NaOH solution. 3,6-bis(3,4-dimethyl-1*H*-pyrazol-1-yl)-1,4-dihydro-1,2,4,5-tetrazine **II** (10.5 g, 38.6 mmol) was added and the mixture was dissolved in 430 mL of a 1:2 mixture of DCM/H_2O. Then it was cooled to 0 ºC. Acetic acid (11 mL, 192.8 mmol) was added dropwise while the colour changes from yellow to bright red with the concomitant generation of an orange gas. After addition was completed, the mixture was strongly stirred during 2 h until gas evolution stopped and the organic phase was separated. The aqueous layer was extracted with DCM (2 x 100 mL), the combined organic layers were washed with sat. $NaHCO_3$ (2 x 100 mL) and dried over Na_2SO_4. The solution was filtered and concentrated under vaccum. The obtained dark red solid was suspended in H_2O, filtered on vacuum, washed with H_2O (1 x 50 mL) and cold MeOH (2 x 25 mL) obtaining 8.3 g (80 %) of a bright red powder. **^{1}H NMR** (500 MHz, $CDCl_3$, δ ppm): 2.39 (s, 6H, 2 CH_3), 2.71 (s, 6H, 2 CH_3), 6.19 (s, 2H, 2 CH). **^{13}C NMR** (126 MHz, $CDCl_3$, δ ppm):14.0 (2 CH_3), 14.8 (2 CH_3), 112.1 (2 CH), 144.0 (2 C), 154.6 (2 C), 159.5 (2 C). **HR-MS** (ESI^+, m/z): $[M+Na]^+$= $C_{12}H_{14}N_8Na$, calcd.: 293.1239; found: 293.1239.

Synthesis of 3,6-dihydrazineyl-1,2,4,5-tetrazine **IV**

To a solution of 3-(3,4-dimethyl-1H-pyrazol-1-yl)-6-(3,4-dimethyl-4,5-dihydro-1H-pyrazol-1-yl)-1,2,4,5-tetrazine **III** (8.3 g, 30.7 mmol) in acetonitrile (150 mL) was added hydrazine monohydrate (4.5 mL, 92,1 mmol) obtaining an immediate change of color of the solution from red to maroon. The mixture was refluxed for 2h, cooled to 25 ºC and filtered. The solid was washed with acetonitrile (2 x 50 mL) and dried under high vacuum affording 4.3 g (99%) of a maroon powder. Even when reference 5 reported the ^{13}C NMR spectra of **IV** in $CDCl_3$, the solid we obtained was insoluble in chloroform, acetonitrile and acetone. In deuterated water it decomposed. Thus we proceed with the synthesis without characterizing this step.

3,6-dichloro-1,2,4,5-tetrazine **Cl-Tz-Cl**

3,6-dihydrazineyl-1,2,4,5-tetrazine **IV** (4.3 g, 27.5 mmol) was suspended in acetonitrile (200 mL) and cooled to 0 ºC with an ice-bath and the gas outlet of the dropping funnel connected to a trap (1M NaOH solution). Trichloroisocianuric acid (19.2 g, 82.6 mmol) was dissolved in acetonitrile (75 mL) and added dropwise to the solution that immediately changes from maroon to bright orange and a white precipitate appears. Once the addition finishes, the solution was let to reach room temperature and it remains at that temperature for 30 min. Then, the reaction mixture was filtered through a celite pad and it was washed with cold acetonitrile. The solvent was evaporated under vacuum and the resulting orange oil was purified by column chromatography (DCM/n-Hex, 1:1) obtaining 4.2 g (99%) of bright orange crystals. **^{13}C NMR** (126 MHz, $CDCl_3$, δ ppm): 168.3 (2 C).

Synthesis of O, O-tetrazine derivatives.

To a solution of 3,6-dichloro-1,2,4,5-tetrazine **Cl-Tz-Cl** (1 equivalent) in DCM (0.1M) was added the corresponding phenol (2.2 equivalents) and Et_3N (2.2 equivalents) and the reaction mixture was stirred at room temperature for 3 h. The solvent was removed under vacuum and the afforded residue was purified by silica gel flash column chromatography to afford pure product.

3,6-bis(p-tolyloxy)-1,2,4,5-tetrazine (**1-Tz-1**). The general procedure was followed using p-cresol **1** (2.2 mmol, 0.237 g) and purified by silica gel flash column chromatography using an elution of hexane/dichloromethane (20:80), affording 0.161 g (55%) of a red solid identified as **1-Tz-1**. (m.p. 147-149 ºC). **^{1}H NMR** (500 MHz, $CDCl_3$, *δ* ppm): 2.38 (s, 6H, 2 CH_3), 7.14 (d, J = 8.5 Hz, 4H, 4 CH), 7.24 (d, J = 8.5 Hz, 4H, 4 CH). **^{13}C NMR** (126 MHz, $CDCl_3$, *δ* ppm): 21.1 (2 CH_3), 120.7 (4 CH), 130.7 (4 CH), 136.4 (2 C), 150.4 (2 C), 167.5 (2 C). **IR** (KBr): ῡ = 3046 (w), 2857 cm^{-1} (w, C-H methyl); 1598 (s), 1508 cm^{-1} (w, C=C); 1196 cm^{-1} (s, C-O-C asymmetric); 1064 (s), 1013 cm^{-1} (s, C-H in plane). **UV/Vis** ($CHCl_3$): λ_{max} (ε)= 352 (5223), 535 nm (1962 $mol^{-1}dm^3cm^{-1}$). **HR-MS** (ESI^+, m/z): $[M+Na]^+$ = $C_{16}H_{14}N_4NaO_2$, calcd.: 317.1014; found 317.1019. **Anal.** Calcd. for $C_{16}H_{14}N_4O_2$: C, 65.30; H, 4.79; N, 19.04; O, 10.8; Found: C,65.10; H,4.97; N, 18.92; O, 11.01.

3,6-bis(4-methoxyphenoxy)-1,2,4,5-tetrazine (**2-Tz-2**). The general procedure was followed using 4-methoxyphenol **2** (2.2 mmol, 0.272 g) and purified by silica gel flash column chromatography using an elution of hexane/dichloromethane (2:8), affording 0.312 g (96%) of a red solid identified as **2-Tz-2**. (m.p. 164-166 ºC). **^{1}H NMR** (500 MHz, $CDCl_3$, *δ* ppm): 3.82 (s, 6H, 2 OCH_3), 6.95 (d, J = 9.2 Hz, 4H, 4 CH), 7.18 (d, J = 9.2 Hz, 4H, 4 CH). **^{13}C NMR** (126 MHz, $CDCl_3$, *δ* ppm): 55.8 (2 OCH_3), 115.1 (4 CH), 122.0 (4 CH), 146.0 (2 C), 157.8 (2 C), 167.7 (2 C). **IR** (KBr): ῡ = 2971 (w), 2838 cm^{-1} (w, C-H methoxy); 1385 cm^{-1} (s, C-H methoxy); 1249 cm^{-1} (s, alkyl-aryl ether); 1175 cm^{-1} (s, C-O-Tz); 840 (s), 803 cm^{-1} (m, 1,4-aromatic). **UV/Vis** ($CHCl_3$): λ_{max} (ε)= 315 (7497), 537 nm (1640 $mol^{-1}dm^3cm^{-1}$). **HR-MS** (ESI^+, m/z): $[M+Na]^+$= $C_{16}H_{14}N_4NaO_4$, calcd.: 349.0913; found 349.0914.

3,6-bis(4-bromophenoxy)-1,2,4,5-tetrazine (**3-Tz-3**). The general procedure was followed using 4-bromophenol **3** (0.81 mmol, 0.101 g) and purified by silica gel flash column chromatography using an elution of hexane/dichloromethane (1:1), affording 0.060 g (38%) of a red solid identified as **3-Tz-3**. (m.p. 220-221 ºC). **^{1}H NMR** (500 MHz, $CDCl_3$, *δ* ppm): 7.17 (d, *J* = 9.4 Hz, 4H, 4 CH), 7.58 (d, *J* = 9.4 Hz, 4H, 4 CH). **^{13}C NMR** (126 MHz, $CDCl_3$, *δ* ppm): 119.8 (2 C), 122.8 (4 CH), 133.3 (4 CH), 151.4 (2 C), 167.3 (2 C). **IR** (KBr): ῡ = 1161 cm^{-1} (s, C-O-Tz); 847 (w); 831 cm^{-1} (s, 1,4-aromatic); 642 cm^{-1} (s. C-Br). **UV/Vis** ($CHCl_3$): λ_{max} (*ε*)= 342 (4642), 533 nm (1459 $mol^{-1}dm^3cm^{-1}$). **HR-MS** (ESI^+, m/z): $[M+Na]^+$= $C_{14}H_8Br_2N_4NaO_2$, calcd.:

444.8912; found 444.8916. **Anal.** Calcd. for $C_{14}H_8Br_2N_4O_2$: C, 39.54; H, 1.88; N, 12.84; Found: C, 39.65; H, 1.90; Br, 37.69; N, 13.21; O, 7.55.

3,6-bis(perfluorophenoxy)-1,2,4,5-tetrazine (**4-Tz-4**). The general procedure was followed using 2,3,4,5,6-pentafluorophenol **4** (2.2 mmol, 0.404 g) affording 0.231 g (52%) of a red solid identified as **4-Tz-4**. (m.p. 171-172 ºC). **^{13}C NMR** (126 MHz, $CDCl_3$, δ ppm): 137.3 (2 CF), 139.5 (4 CF), 140.3 (2 CF), 142.4 (4 CF), 166.6 (2 C). **IR** (KBr): $\tilde{\upsilon}$ = 1369 cm^{-1} (vs, C-F); 992 cm^{-1} (s, pentasubstituted aromatic); 733 cm^{-1} (w, C-O). **UV/Vis** ($CHCl_3$): λ_{max} (ε)= 321 (5676), 519 nm (1236 $mol^{-1}dm^3cm^{-1}$). **HR-MS** (ESI^-, m/z): $[M]^-$= $C_{14}F_{10}N_4O_2$, calcd.: 445.9862; found 445.9867. **Anal.** Calcd. for $C_{14}F_{10}N_4O_2$: C, 37.69; F, 42.58; N, 12.56; O, 7.17; Found: C, 37.92; N, 12.81.

3,6-bis(2-isopropylphenoxy)-1,2,4,5-tetrazine (**5-Tz-5**). The general procedure was followed using 2-isopropylphenol **5** (1.18 mmol, 0.16 mL) and purified by silica gel flash column chromatography using an elution of hexane/dichloromethane (7:3),affording 0.153 g (81%) of a red solid identified as **5-Tz-5**. (m.p. 131-133 ºC). **^{1}H NMR** (500 MHz, $CDCl_3$, δ ppm): 1.25 (d, J = 7.2 Hz, 12H, 4 CH_3), 3.18 (hept, J = 6.9 Hz, 2H, 2 CH), 7.12 (dd, J = 7.7 Hz, 1.7 Hz, 2H, 2 CH), 7.25-7.33 (m, 4H, CH), 7.44 (dd, J = 7.6 Hz, 2.5 Hz, 2H, 2 CH). **^{13}C NMR** (126 MHz, $CDCl_3$, δ ppm): 23.2 (4 CH_3), 27.5 (2 CH), 121.2 (2 CH), 127.1 (2 CH), 127.4 (2 CH), 127.6 (2 CH), 140.4 (2 C), 150.1 (2 C), 167.6 (2 C). **IR** (KBr): $\tilde{\upsilon}$ = 2962 (w), 2875 cm^{-1} (w, C-H methyl); 1178 cm^{-1} (s, C-O-C asymmetric); 875 cm^{-1} (1,4-aromatic); 790 (m), 752 cm^{-1} (o-substitued aromatic). **UV/Vis** ($CHCl_3$): λ_{max} (ε)= 342 (11476), 537 nm (2662 $mol^{-1}dm^3cm^{-1}$). **HR-MS** (ESI^+, m/z): $[M+Na]^+$= $C_{20}H_{22}N_4NaO_2$, calcd.: 373.1640; found 373.1642. **Anal.** Calcd. for $C_{20}H_{22}N_4NO_2$: C, 68.55; H, 6.33; N, 15.99; O, 9.13; Found: C, 68.47; H, 6.61; N, 15.92; O, 9.00.

3,6-bis(2-(prop-2-yn-1-yloxy)phenoxy)-1,2,4,5-tetrazine (**6-Tz-6**). The general procedure was followed using 2-(prop-2-yn-1-yl)phenol **6** (0.4 mmol, 0.059 g) and purified by silica gel flash column chromatography using an elution of hexane/dichloromethane (1:1), affording 0.050 g (67%) of a red solid identified as **6-Tz-6**. (m.p. 176-178 ºC). **^{1}H NMR** (500 MHz, CD_3CN, δ ppm): 2.79 (t, J = 2.3 Hz, 2H, 2 CCH), 4.69 (d, J = 2.35 Hz, 4H, 2 CH_2), 7.12 (dt, J = 7.7 Hz, 1.5 Hz, 2H, 2 CH), 7.23 (dd, J = 8.3 Hz, 1.2 Hz, 2H, 2 CH), 7.33-7.36 (m, 4H, CH). **^{13}C NMR** (126 MHz, CD_3CN,

δ ppm): 57.4 (2 CH_2), 77.2 (2 CC), 78.9 (2 CC), 115.7 (2 CH), 123.2 (2 CH), 128.3 (2 CH), 142.6 (2 C), 149.8 (2 C), 168.1 (2 C). **IR** (KBr): ῡ = 3750 cm^{-1} (br, Ph-OH); 3279 (s), 3255 cm^{-1} (s, C-H alkyne); 2122 cm^{-1} (w, C-C alkyne); 1390 cm^{-1} (m, Ph-OH); 1261 cm^{-1} (s, -O-CH_2-alkyne); 1182 cm^{-1} (s, C-O-Tz); 732, 750 cm^{-1} (s, 1,4-aromatic overtones). **UV/Vis** ($CHCl_3$): λ_{max} (ε)= 330 (12462), 525 nm (2561 $mol^{-1}dm^3cm^{-1}$). **HR-MS** (ESI^+, m/z): $[M+Na]^+$= $C_{20}H_{14}N_4NaO_2$, calcd.: 397.0913; found 397.0915.

4,4'-(1,2,4,5-tetrazine-3,6-diyl)dibenzaldehydetetrazine **(10-Tz-10)**. The general procedure was followed using 4-hydroxybenzaldehyde **10** (2.2 mmol, 0.269 g) and purified by silica gel flash column chromatography using an elution of dichloromethane, affording 0.168 g (52%) of a red solid identified as **10-Tz-10**. (m.p. 227-228 ºC). **^{1}H NMR** (500 MHz, CD3CN, δ ppm): 7.52 (d, J = 8.4 Hz, 4H, 4 CH), 8.02 (d, J = 8.4 Hz, 4H, 4 CH), 10.02 (s, 2H, 2 CHO). **^{13}C NMR** (126 MHz, CD3CN, δ ppm): 122.4 (4 CH), 132.6 (4 CH), 135.6 (2 C), 157.9 (2 C), 168.2 (2 C), 192.2 (2 C). **IR** (KBr): ῡ = 3072 (w), 2850 cm−1 (w, C-H aldehyde); 1691 cm−1 (s, C=O); 1205 cm−1 (s, C-O-Tz); 853 (s), 819 (s, 1,4-aromatic). **UV/Vis** (CHCl3): λmax (ε)= 339 (11677), 533 nm (2977 mol−1dm3cm−1). **HR-MS** (ESI-, m/z): [M+Cl]-= $C_{16}H_{10}N_4$ $^{35}ClO_2$, calcd.: 357.0391; found 357.0399. [M+Cl]-= $C_{16}H_{10}N_4$ $^{37}ClO_2$, calcd.: 359.0361; found 359.0367. **Anal.** Calcd. for $C_{16}H_{10}N_4O_2$: C, 59.63; H, 3.13; N, 17.38; O, 19.86; Found: C, 59.56; H, 3.41; N, 17.54; O, 19.49.

1,1'-((1,2,4,5-tetrazine-3,6-diyl)bis(4,1-phenylene))bis(N-p-tolylmethanimine) **(11-Tz-11)**. To a solution of 4,4'-(1,2,4,5-tetrazine-3,6-diyl)dibenzaldehydetetrazine **(10-Tz-10)** (0.02 mmol, 6.4 mg) in CD_3CN (0.5 mL) in a NMR tube, was added p-methylaniline (0.1 mmol, 10.7 mg). The reaction mixture was shaken at room temperature and inmediatly a red precipitate started to appear. After 1 h no clear signals were observed in the H NMR. The reaction mixture was filtered, washed with acetonitrile and the solvent was removed under vacuum in a rotavap. The resulting red solid (5.6 mg, 0.011 mmol, 56% yield) was solved in DMSO-*d6* and it was identified as the double imine **11-Tz-11**. **^{1}H NMR** (400 MHz, DMSO-d6, δ ppm): 2.33 (s, 6H, 2 CH3), 7.23 (d, J = 9.2 Hz, 8H, 8 CH), 7.54 (d, J = 8.7 Hz, 4H, 4 CH), 8.07 (d, J = 8.7 Hz, 4H, 4 CH), 8.67 (s, 2H, 2 CNC).

Synthesis of S, S-tetrazine derivatives

To a solution of 3,6-dichloro-1,2,4,5-tetrazine **Cl-Tz-Cl** (1 equivalent) in DCM (0.1M) was added the corresponding thiol (2.2 equivalents) and Et_3N (2.2 equivalents) and the reaction mixture was stirred at room temperature for 3 h. The reaction mixture solvent was removed under vacuum and the afforded oil was purified by silica gel flash column chromatography to afford the product.

3,6-bis(dodecylthio)-1,2,4,5-tetrazine (**7-Tz-7**). The general procedure was followed using dodecane-1-thiol **7** (1.73 mmol, 0.4 mL) and purified by silica gel flash column chromatography using an elution of 0-1% of ethyl acetate in hexane, affording 0.281 g (81 %) of a red solid identified as **7-Tz-7**. (m.p. 72-74 ºC). **^{1}H NMR** (500 MHz, $CDCl_3$, δ ppm): 0.88 (t, J = 7.1 Hz, 4H, CH_3), 1.26-1.34 (m, 32H, CH_2), 1.44-.150 (m, 4H, CH_2), 1.75-1.81 (m, 4H, CH_2), 3.27 (t, J = 7.4 Hz, 2H, CH_2). **^{13}C NMR** (126 MHz, $CDCl_3$, δ ppm): 14.3 (2 CH_3), 22.9 (2 CH_2), 28.9 (2 CH_2), 29.0 (2 CH_2), 29.2 (2 CH_2), 29.5 (2 CH_2), 29.6 (2 CH_2), 29.7 (2 CH_2), 29.8 (2 CH_2), 29.9 (2 CH_2), 30.7 (2 CH_2), 32.1 (2 CH_2), 172.9 (2 C). **IR** (KBr): ῦ = 2935 (s), 2866 cm^{-1} (s, C-H); 1464 cm^{-1} (s, C-H bending); 1261 (s), 1230 cm^{-1} (s, CH_3); 875 (s), 803 cm^{-1} (w, 1,4-aromatic); 714 cm^{-1} (m, C-S). **UV/Vis** ($CHCl_3$): λ_{max} (ε)= 426 (2853), 529 nm (1793 $mol^{-1}dm^3cm^{-1}$). **HR-MS** (ESI^+, m/z): $[M+Na]^+$= $C_{26}H_{50}N_4NaS_2$, calcd.: 505.3375; found 505.3381. **Anal.** Calcd. for $C_{26}H_{50}N_4S_2$: C, 64.05; H, 10.32; N, 11.95; S, 13.68; Found: C, 63.92; H, 10.46; N, 11.63; S, 13.74.

3,6-bis(benzylthio)-1,2,4,5-tetrazine (**8-Tz-8**). The general procedure was followed using phenylmethanethiol **8** (1.79 mmol, 0.21 mL) and purified by silica gel flash column chromatography using an elution of 1-5% of ethyl acetate in hexane, affording 0.176 g (73%) of a red solid identified as **8-Tz-8**. (m.p. 157-159 ºC). **^{1}H NMR** (500 MHz, $CDCl_3$, δ ppm): 4.51 (s, 4H, CH_2), 7.26-7.36 (m, 6H, CH), 7.45 (d, J = 7.2 Hz, 4H, CH). **^{13}C NMR** (126 MHz, $CDCl_3$, δ ppm): 35.0 (2 CH_2), 128.0 (2 CH), 128.9 (4 CH), 129.4 (4 CH), 135.7 (2 C), 175.5 (2 C). **IR** (KBr): ῦ = 3084 (w), 3033 cm^{-1} (w, C-H); 1452 cm^{-1} (m, C-H bending); 882 (m), 802 cm^{-1} (w, 1,4-aromatic); 719 cm^{-1} (s, C-S). **UV/Vis** ($CHCl_3$): λ_{max} (ε)= 422 (2496), 531 nm (1659 $mol^{-1}dm^3cm^{-1}$). **HR-MS** (ESI^+, m/z): $[M+Na]^+$= $C_{16}H_{14}N_4NaS_2$, calcd.: 349.0556; found 349.0558. **Anal.** Calcd. for

$C_{16}H_{14}N_4S_2$: C, 58.87; H, 4.32; N, 17.16; S, 19.64; Found: C, 58.6; H, 4.66; N, 17.26; S, 19.79.

3,6-bis(2-sodium ethanesulfonate thio)-1,2,4,5-tetrazine (**9-Tz-9**). A solution of 3,6-dichloro-1,2,4,5-tetrazine **Cl-Tz-Cl** (1 mmol, 151 mg) in 10 mL of $CHCl_3$ (0.1M) and a solution of sodium 2-mercaptoethanesulfonate **9** (2 mmol, 328 mg) in distilled water (10mL) were shaken together on an extraction funnel for 2 minutes. After that time, the aqueous phase was colored while the organic one was almost colorless. The aqueous phase was separated and dried under reduced pressure. The resulting orange solid was dried under high vacuum yielding 329 mg of **9-Tz-9** (81%). (m.p. 295-297 ºC "gas release"). **^{1}H NMR** (500 MHz, $CDCl_3$, δ ppm): 3.72-3.65 (m, 4H, CH_2), 3.43-3.37 (m, 4H, CH_2). **^{13}C NMR** (126 MHz, $CDCl_3$, δ ppm): 24.8 (2 CH_2), 49.8 (2 CH), 171.8 (2C). **IR** (KBr): ῦ = 3609 (m), 3540 cm−1 (m, C-H); 1204 (s), 1170 (s), 1046 cm^{-1} (s, S=O); 803 cm−1 (m, 1,4-aromatic); 742 cm−1 (m, C-S from SO_3Na); 717 cm^{-1} (w, C-S). **UV/Vis** (H_2O): λ_{max} (ε)= 415 (833), 501 nm (402 $mol^{-1}dm^3cm^{-1}$). **HR-MS** (ESI^-, m/z): $[M\text{-}Na]^-$= $C_6H_8N_4NaO_6S_4$, calcd.: 382.9224; found 382.9228.

Synthesis of O, S-tetrazine derivative

The synthesis of this kind of tetrazine derivatives (heterodimers) is more complicated than that of O, O- or S, S-tetrazines (homodimers). Indeed, the tendency is to obtain the homodimers and just tiny amounts of the heterodimer. The first attempts were to synthesize the monosubstituted tetrazine **1-Tz-Cl** or **7-Tz-Cl**. The latter was obtained in very poor yield in dichloromethane and in acetonitrile. Starting from **1-Tz-Cl**, adding 1 equivalent of thiol **7**, only **7-Tz-7** was isolated. Thus we decided to substitute one of the cresols from **1-Tz-1** with the thiol:

3-(dodecylthio)-6-(p-tolyloxy)-1,2,4,5-tetrazine (1-Tz-7)

To a solution of 3,6-bis(4-methylphenoxy)-1,2,4,5-tetrazine (**1-Tz-1**) (0.45 mmol, 0.133 g) in DCM (45 mL) was added a solution of dodecanethiol **7** (0.27 mmol, 0.05mL) and DIPEA (0.23 mmol, 0.05 mL) in DCM (10 mL) dropwise. The reaction mixture was

stirred at room temperature until addition was complete. Then, reaction mixture solvent was removed under vacuum and the organic layer was washed with a solution of HCl 1M. the organic layer was dry with $MgSO_4$ and solvent was removed under vacuum. Afforded oil was purified by silica gel flash column chromatography using an elution of 1% ethyl acetate in hexane to afford 0.0075 g (4%) of a red solid identified as **1-Tz-7**. (m.p. 59-61 ºC). **^{1}H NMR** (500 MHz, $CDCl_3$, δ ppm): 0.88 (t, J = 7.0 Hz, 3H, CH_3), 1.26-1.33 (m, 15H, CH_2), 1.42-1.48 (m, 2H, CH_2), 1.74-1.80 (m, 2H, CH_2), 2.39 (s, 3H, CH_3), 3.26 (t, J = 7.5 Hz, 2H, SCH_2), 7.14 (d, J = 8.5 Hz, 2H, 2 CH), 7.26(d, J = 8.5 Hz, 2H, 2 CH). **^{13}C NMR** (126 MHz, $CDCl_3$, δ ppm): 14.3 (CH_3), 21.1 (CH_2), 22.8 (CH_2), 28.90 (CH_2), 29.0 (CH_2), 29.2 (CH_2), 29.5 (CH_2), 29.6 (CH_2), 29.7 (CH_2), 29.8 (CH_3), 29.9 (CH_2), 31.1 (CH_2), 32.0 (CH_2), 120.7 (2 CH), 130.7 (2 CH), 136.4 (C), 150.0 (C), 167.3 (C) 173.1 (C). **IR** (KBr): ῡ = 2915 (s), 2860 cm^{-1} (s, C-H); 1448 cm^{-1} (s, C-H bending); 1058 (s), 1018 cm^{-1} (s, C-H in plane).; 814 (s), 795 cm^{-1} (w, 1,4-aromatic); 718 cm^{-1} (m, C-S). **UV/Vis** ($CHCl_3$): λ_{max} (ε)= 404 (4999), 534 nm (2512 $mol^{-1}dm^3cm^{-1}$). **HR-MS** (ESI^+, m/z): $[M+Na]^+$= $C_{21}H_{32}N_4NaOS$, calcd.: 411.2195; found 411.2197. **Anal.** Calcd. for $C_{21}H_{32}N_4NaOS$: C, 64.91; H, 8.30; N, 14.42; O, 4.12; S, 8.25; Found: C, 65.03; H, 8.00; N, 14.41; O, 3.93; S, 8.63.

Inverse Electron Demand Diels Alder Reactions.

Synthesis of 3,6-bis(4-methoxyphenoxy)pyridazine 2-Dz-2

To a solution of 3,6-bis(4-methoxyphenoxy)-1,2,4,5-tetrazine (**2-Tz-2**) (0.32 mmol, 0.105 g) in $CHCl_3$ (5 mL) dimethyl-1,4-dimethyl-7-oxabicyclo[2.2.1]hepta-2,5-diene-2,3-dicarboxylate (0.89 mmol, 0.211 g) was added and the reaction mixture was stirred at 50 ºC for 48 h. The reaction mixture solvent was removed under vacuum and to the afforded residue ether was added and a white precipitate was formed (0.104 g, 94%) of a white solid identified as **2-Dz-2**. (m.p. 195-197 ºC). **^{1}H NMR** (500 MHz, $CDCl_3$, δ ppm): 3.79 (s, 6H, 2 OCH_3), 6.88 (d, J = 9.0 Hz, 4H, 4 CH), 7.10 (d, J = 9.0 Hz, 4H, 4 CH), 7.18 (s, 2H, 2 CH). **^{13}C NMR** (126 MHz, $CDCl_3$, δ ppm): 55.8 (2 OCH_3), 114.8 (4 CH), 121.6 (2 CH), 122.3 (4 CH), 147.2 (2 C), 157.0 (2 C), 163.4 (2 C). **IR** (KBr): ῡ = 2960 (w), 2835 cm^{-1} (w, C-H methoxy); 1615 (w), 1595 cm^{-1} (w, C=C); 1428 cm^{-1} (s, C-H methoxy); 1243 cm^{-1} (s, alkyl-aryl ether); 1184 cm^{-1} (s, C-O-Tz); 832 (s), 814 cm^{-1} (w, 1,4-aromatic). UV/Vis (*$CHCl_3$*): λ_{max} (ε)= 279 nm (16497 $mol^{-1}dm^3cm^{-1}$). **HR-MS**

(ESI$^+$, m/z): $[M+Na]^+= C_{18}H_{16}N_2NaO_4$, calcd.: 347.1008; found 347.1012. **Anal.** Calcd. for $C_{18}H_{16}N_2O_4$: C, 66.66; H, 4.97; N, 8.64; O, 19.73; Found: C, 66.68; H, 5.03; N, 8.60; O, 19.69.

3-(2-(prop-2-yn-1-yloxy)phenyl)-5H-benzo[2,3][1,4]dioxepino[5,6-c]pyridazine 6-Dz

A solution of 3,6-bis(2-(prop-2-yn-1-yloxy)phenoxy)-1,2,4,5-tetrazine (**6-Tz-6**). (1 mmol, 0.397 g) in CH_3CN (10 mL) was stirred at 80 ºC for 72 h. The reaction mixture solvent was removed under vacuum and the afforded oil was purified by silica gel flash column chromatography using an elution of dichloromethane, to afford 0.332 g (94%) of a white solid identified as **6-Dz**. (m.p. 150-152 ºC). ^{1}**H NMR** (500 MHz, $CDCl_3$, δ ppm): 2.72 (t, J = 2.4 Hz, 1H, CCH), 4.66 (d, J = 2.4 Hz, 2H, CH_2), 5.20 (s, 2H, CH_2), 7.04-7.12 (m, 4H, CH), 7.19-7.24 (m, 2H, CH), 7.26-7.32 (m, 3H, CH). ^{13}**C NMR** (126 MHz, $CDCl_3$, δ ppm): 57.3 (CH_2), 69.5 (CH_2), 77.0 (CCH), 79.2 (CCH), 115.9 (CH), 118.9 (CH), 122.4 (CH), 122.8 (CH), 123.1 v123.9 (CH), 125.0 (CH), 126.2 (CH), 127.4 (CH), 133.7 (C), 143.7 (C), 145.9 (C), 149.2 (C), 150.3 (C), 163.7 (C), 164.4 (C). **IR** (KBr): ῦ = 3488 cm^{-1} (br, Ph-OH); 3283 (s), 3073 cm^{-1} (s, C-H alkyne); 2141 cm^{-1} (w, C-C alkyne); 1490 cm^{-1} (m, C-H bending); 1361 cm^{-1} (w, Ph-OH); 1235 cm^{-1} (s, -O-CH_2-alkyne); 1181 cm^{-1} (s, C-O-Tz). **UV/Vis** ($CHCl_3$): λ_{max} (ε)= 300 nm (9886 $mol^{-1}dm^3cm^{-1}$). **HR-MS** (ESI$^+$, m/z): $[M+Na]^+= C_{20}H_{14}N_2NaO_4$, calcd.: 369.0846; found 369.0839. **Anal.** Calcd. for $C_{20}H_{14}N_2O_4$: C, 69.36; H, 4.07; N, 8.09; O, 18.48; Found: C, 69.46; H, 4.06; N, 7.79; O, 18.70.

Synthesis of Dimethyl-1,4-dimethyl-7-oxabicyclo[2.2.1]hepta-2,5-diene-2,3-dicarboxylate 12

To a solution of dimethyl 2-butynedioate, commonly called dimethyl acetylenedicarboxylate (7.15 mmol, 0.88 mL) in dioxane (5 mL), 2, 5-dimethylfurane was added (9.3 mmol, 0.903 g) and the reaction mixture was stirred at reflux for 24 h. The reaction mixture solvent was removed under vacuum and the afforded oil was purified by silica gel flash column chromatography using an elution of ethyl acetate/hexane (2:8) to afford 1.251 g (73%) of a yellow oil identified as **12**. ^{1}**H NMR** (500 MHz, $CDCl_3$, δ ppm): 1.79 (s, 6H, 2 CH_3), 3.79 (s, 6H, 2 OCH_3), 6.94 (s, 2H, 2 CH). ^{13}**C NMR** (126 MHz, $CDCl_3$, δ ppm): 15.5 (2 CH_3), 52.3 (2 OCH_3), 92.2 (2 C), 147. (2 =CH), 155.0 (2 =C),

164.5 (2 CO). **HR-MS** (ESI$^+$, m/z): $[M+Na]^+$= $C_{12}H_{14}NaO_5$, calcd.: 261.0739; found 261.0741.

Synthesis of macrocycle 15

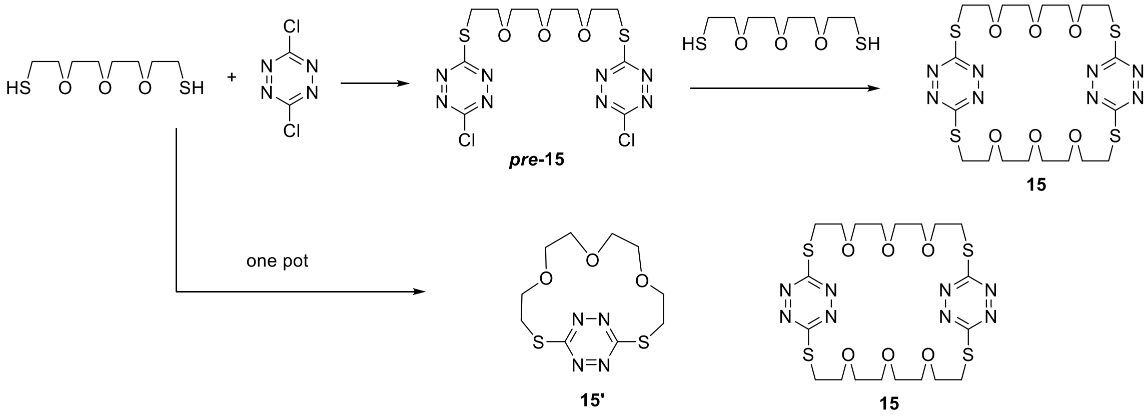

Figure S2.

Dithiol 2,2'-((oxybis(ethane-2,1-diyl))bis(oxy))bis(ethane-1-thiol) was synthesized as previously reported.[6]

One pot procedure

To a solution of diisopropylethylamine (2.54 mmol, 0.44 mL) in 50 mL of tetrahydrofuran, was simultaneously added dropwise a solution of **Cl-Tz-Cl** (1.27 mmol, 0.191 g) in THF (20 mL) and a solution of 2,2'-((oxybis(ethane-2,1-diyl))bis(oxy))bis(ethane-1-thiol) (1.27 mmol, 0.287 g) in THF (20 mL) and the reaction mixture was stirred at room temperature for 16 h. The reaction mixture solvent was removed under vacuum and the afforded oil was purified by silica gel flash column chromatography using an elution of ethyl acetate/hexane (4:8→8:2) to afford 0.232 g (60%) of a red solid identified as **15'** (m.p. 121-123 ºC) and 0.070 g (9%) of **15** (m.p. 104-105 ºC).

Characterization of **15'**

^{1}H NMR (500 MHz, $CDCl_3$, δ ppm): 3.80 (dd, J = 5.7, 4.7 Hz, 4H, 2 CH_2), 3.61 (dd, J = 5.7, 4.7 Hz, 4H, 2 CH_2), 3.52 – 3.48 (m, 4H, 2 CH_2), 3.36 – 3.31 (m, 4H, 2 CH_2). **^{13}C**

NMR (126 MHz, $CDCl_3$, δ ppm): 31.0, 70.5, 70.7, 71.5, 172.3. **IR** (KBr): ῦ = 2904 (m), 2857 cm^{-1} (m, C- H); 1245 cm^{-1} (s, alkyl-aryl ether); 1134 (m), 1098 cm^{-1} (m, C-O-C asymetric); 878 (m), 826 cm^{-1} (w, 1,4-aromatic); 673 cm^{-1} (w, C-S). **UV/Vis** (*$CHCl_3$*): λ_{max} (ε)= 408 (1491), 522 nm (944 $mol^{-1}dm^3cm^{-1}$). **HR-MS** (ESI^+, m/z): $[M+Na]^+$= $C_{10}H_{16}N_4O_3NaS_2$, calcd.: 327.0562; found 327.0559. **Anal.** Calcd. for $C_{10}H_{16}N_4O_3S_2$: C, 39.46; H, 5.30; N, 18.41; O, 15.77; S, 21.07; Found: C, 39.67; H, 5.45; N, 18.8; O, 14.98, S, 21.10.

Characterization of **15**

^{1}H NMR (500 MHz, $CDCl_3$, δ ppm): 3.55 (t, *J* = 6.0 Hz, 8H, 4 CH_2), 3.62-3.67 (m, 16H, 8 CH_2), 3.84 (t, *J* = 6.0 Hz, 8H, 4 CH_2). **^{13}C NMR** (126 MHz, $CDCl_3$, δ ppm): 30.4 (4 CH_2), 69.1 (4 CH_2), 70.7 (4 CH_2), 70.9 (4 CH_2), 172.6 (4 C). **IR** (KBr): ῦ = 2944 (m), 2866 cm^{-1} (m, C- H); 1235 cm^{-1} (s, alkyl-aryl ether); 1106 (m), 1089 cm^{-1} (m, C-O-C asymetric); 876 (m), 823 cm^{-1} (w, 1,4-aromatic); 672 cm^{-1} (w, C-S). **UV/Vis** ($CHCl_3$): λ_{max} (ε)= 423 (4917), 528 nm (3097 $mol^{-1}dm^3cm^{-1}$). **HR-MS** (ESI^+, m/z): $[M+Na]^+$= $C_{20}H_{32}N_8NaO_6S_4$, calcd.: 631.1225; found 631.1228. **Anal.** Calcd. for $C_{20}H_{32}N_8O_6S_4$: C, 39.46; H, 5.30; N, 18.41; O, 15.77; S, 21.07; Found: C, 39.77; H, 5.51; N, 18.24; O, 15.70, S, 20.78.

Two-step procedure

To a solution of **Cl-Tz-Cl** (2.92 mmol, 0.441 g) in THF (10 mL) was added dropwise a solution of 2,2'-((oxybis(ethane-2,1-diyl))bis(oxy))bis(ethane-1-thiol) (1.46 mmol, 0.331 g) and diisopropylethylamine (2.92 mmol, 0.52 mL) in THF (5 mL) and the reaction mixture was stirred at room temperature for 16 h. The solvent was removed under vacuum and the afforded oil was purified by silica gel flash column chromatography using an elution of ethyl acetate/hexane (8:2) to afford 0.412 g (62%) of a red solid identified as ***pre*-15**. **^{1}H NMR** (500 MHz, $CDCl_3$, δ ppm): 3.84 (t, *J* = 6.2 Hz, 4H), 3.64 (dddt, *J* = 7.6, 5.8, 4.1, 1.9 Hz, 8H), 3.55 (t, *J* = 6.1 Hz, 4H). **^{13}C NMR** (126 MHz, $CDCl_3$, δ ppm): 30.9, 68.8, 70.7, 165.8, 176.0. **HR-MS** (ESI^+, m/z): $[M+Na]^+$= $C_{14}H_{22}N_8O_5NaS_2$, calcd.: 469.1052; found 469.1048.

To a solution of diisopropylethylamine (0.36 mmol, 0.063 mL) in 20 mL of THF, was simultaneously added dropwise a solution of ***pre*-15** (0.18 mmol, 0.082 g) in THF (12 mL) and a solution of 2,2'-((oxybis(ethane-2,1-diyl))bis(oxy))bis(ethane-1-thiol) (0.18 mmol, 0.041 g) in THF (12 mL) and the reaction mixture was stirred at room temperature for 16 h. The reaction mixture solvent was removed under vacuum and the afforded oil was purified by silica gel flash column chromatography using an elution of ethyl acetate/hexane (8:2) to afford 0.078 g (71%) of a red solid identified as **15**.

Synthesis of cage 17

In a 500 mL round bottom flask 200 mL of ACN were added, followed by TEA (0,75 mL, 5,4 mmol). Phloroglucinol (150 mg, 1,2 mmol) and 1,4-dichlorotetrazine (270 mg, 1,8 mmol) were dissolved in 50 mL of ACN and added dropwise to the first solution employing a dropping funnel during a period of 2h. After the addition was complete, the reaction mixture was stirred at room temperature for 30 minutes and then 2 mL of a saturated aqueous solution of NH_4Cl were added and the solvent was evaporated under reduced pressure. The red solid crude was purified by silica gel flash column chromatography using, first, an elution of DCM/Hex (50:50) and then with DCM/Acetone (95:5) affording 0.126 g (43%) of a pink solid. (m.p. ≥ 345 ºC "decomposes"). **^{1}H NMR** (400 MHz, $CDCl_3$, δ ppm): 7.13 (s, 12H, 12 CH). **^{13}C NMR** (101 MHz, $CDCl_3$, δ ppm): 116.0 (CH), 154.0 (C), 167.8 (C). **IR** (KBr): $\tilde{\upsilon}$ = 1620 cm^{-1} (s, C-H aromatic); 1380 cm^{-1} (s, C-O); 1125 cm^{-1} (s, C-O-C asymetric); 891 (m), 854 cm^{-1} (w, 1,3,5-aromatic). **UV/Vis** ($CHCl_3$): λ_{max} (ε)= 334 nm (38822 $mol^{-1}dm^3cm^{-1}$), 532 nm (6417 $mol^{-1}dm^3cm^{-1}$). **HR-MS** (ESI^+, m/z): $[M+Na]^+$= $C_{36}H_{12}N_{24}NaO_{12}$, calcd.: 995.0964; found 995.0977.

Post-functionalization of cage 17: Synthesis of cage 18.

In a 10 mL round bottom flask 1mL of dry acetonitrile and DCM (8:2) were added, followed by **17** (5 mg, 0.005 mmol) and cyclooctyne (56 mg, 0.05 mmol) which was previously synthesized as reported elsewhere.[7] The reaction mixture was stirred at room temperature until the disappearance of the red color. A solid precipitates out of the solution. The solvent was evaporated under reduced pressure. The resulting solid was washed with hexane and dried under high vacuum, affording 4.2 mg (58% yield) of a white-pink solid. (m.p. ≥ 318 ºC "decomposes"). **^{1}H NMR** (500 MHz, $CDCl_3$, δ ppm):

6.75 (s, 12H, 12 CH), 2.85 (t, *J* = 6.3 Hz, 24H), 1.80-1.72 (m, 24H), 1.48-1.41 (m, 24H). **^{13}C NMR** (126 MHz, $CDCl_3$, δ ppm): 24.4 (CH_2), 26.3 (CH_2), 29.3 (CH_2), 115.3 (CH), 132.2 (C), 155.3 (C), 161.6 (C). **IR** (KBr): $\tilde{\upsilon}$ = 3737-2997 cm^{-1} (br, C-H stretching alkane); 2927 cm^{-1} (m, C-H stretching alkene); 1617 cm^{-1} (m, C=C stretching alkene); 1455 cm^{-1} (m, C-H bending alkane); 1375 cm^{-1} (vs, C-O aromatic ether); 1118 cm^{-1} (s, C-O-C asymetric); 977 cm^{-1} (s, C=C bending); 879 (m), 833 cm^{-1} (w, 1,3,5-aromatic). **UV/Vis** ($CHCl_3$): λ_{max} (ε)= 283 nm (26478 mol^{-1}dm^{3}cm^{-1}). **HR-MS** (ESI$^+$, m/z): $[M+Na]^+$= $C_{84}H_{84}N_{24}N_{12}O_{12}Na$, calcd.: 1475.6229; found 1475.6230. **Anal.** Calcd. for $C_{84}H_{84}N_{24}N_{12}O_{12}$: C,69.67; H, 5.95; N, 11.18; O, 13.20; Found: C, 39.77; H, 5.51; N, 18.24; O, 15.70, S, 20.78.

<u>Covalent Dynamic Studies: ^{1}H NMR experiments.</u>

To a solution of the corresponding tetrazine **x-Tz-x** (0.011 mmol) in the corresponding deuterated solvent (0.5 mL) was added the corresponding phenol or thiol (0.022 mmol) and Et_3N (0.033 mmol, 5 µL) and the reaction mixture was monitored by ^{1}H NMR at 25ºC. Unfortunately, in almost every case, the peaks corresponding to homo and heterodimers are undistinguishable. Therefore, proper quantification of all the chemical species in the reaction media cannot be performed by ^{1}H NMR. However, comparing the spectra of the reactions performed in both direction it is clear that equilibrium is reached. For a better visualization, we quantify the ratio of free phenol/thiol versus phenol/thiol attached to tetrazine. In the latter case, the homo- and hetero-dimer were considered together and called Tz-x. Thus, the tendency of the phenol/thiol to be released from the tetrazine or to remain attached to it can be easily examined.

Nonetheless, a proper quantification was performed by q-^{13}C NMR, which is detailed below.

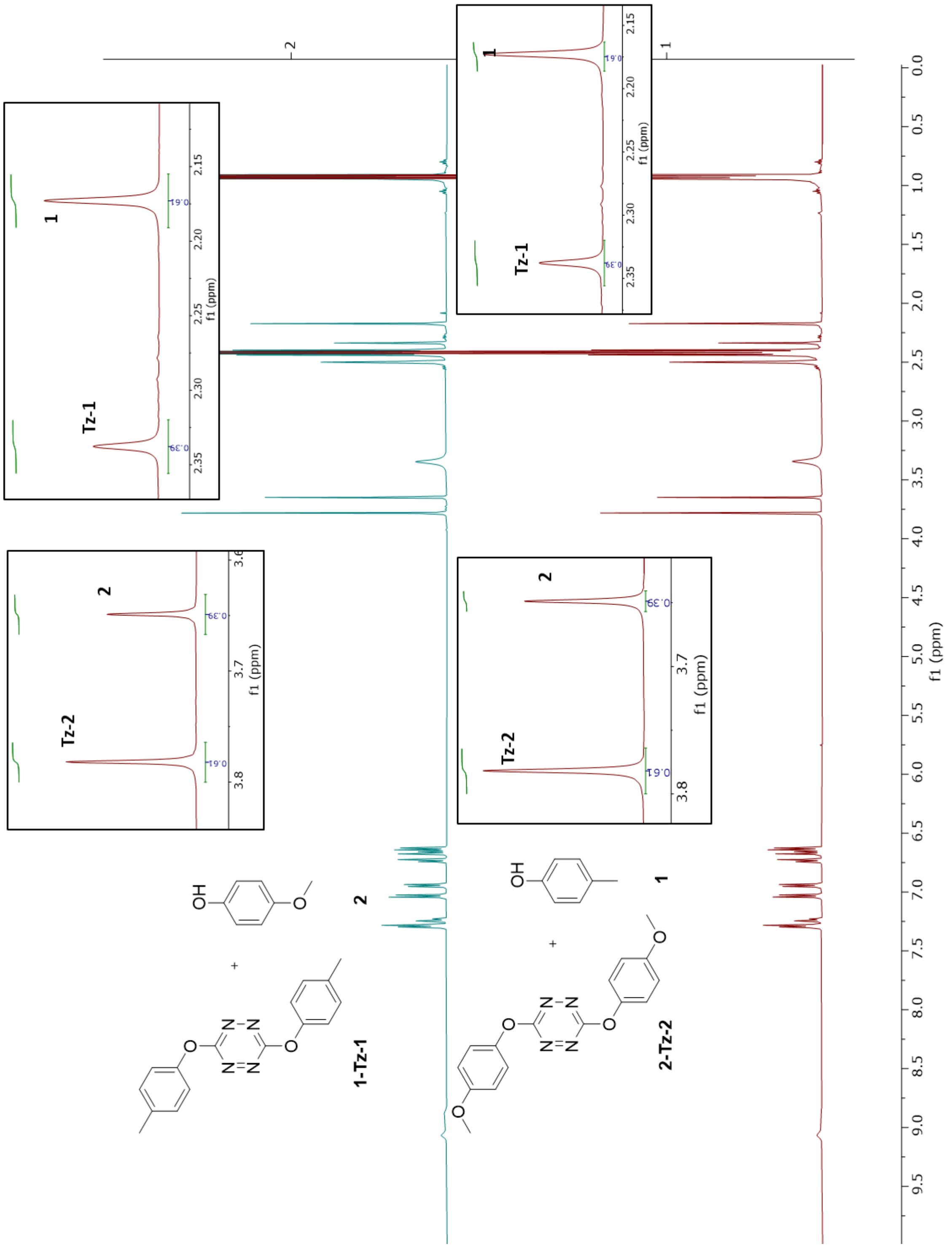

Figure S3

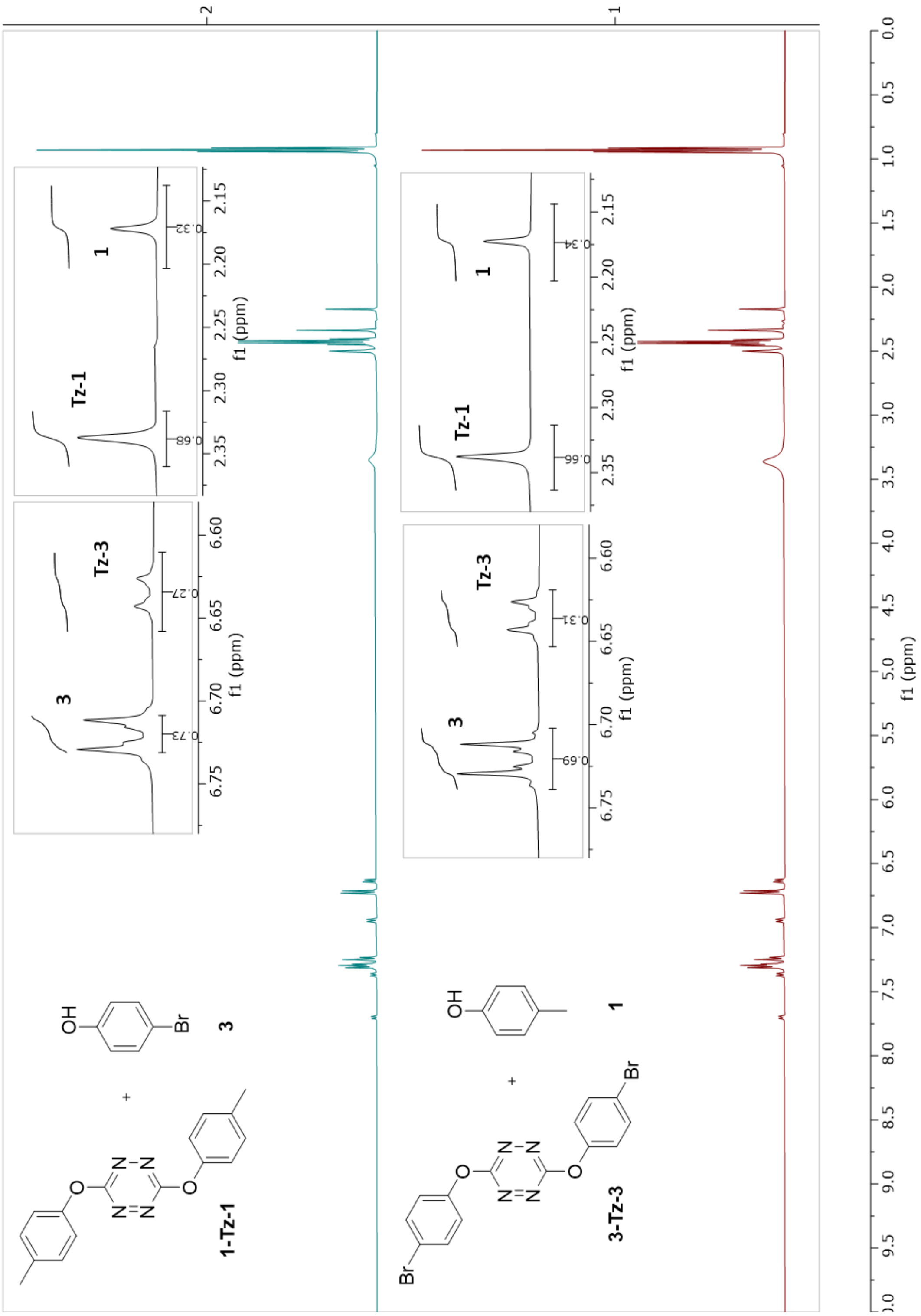

Figure S4

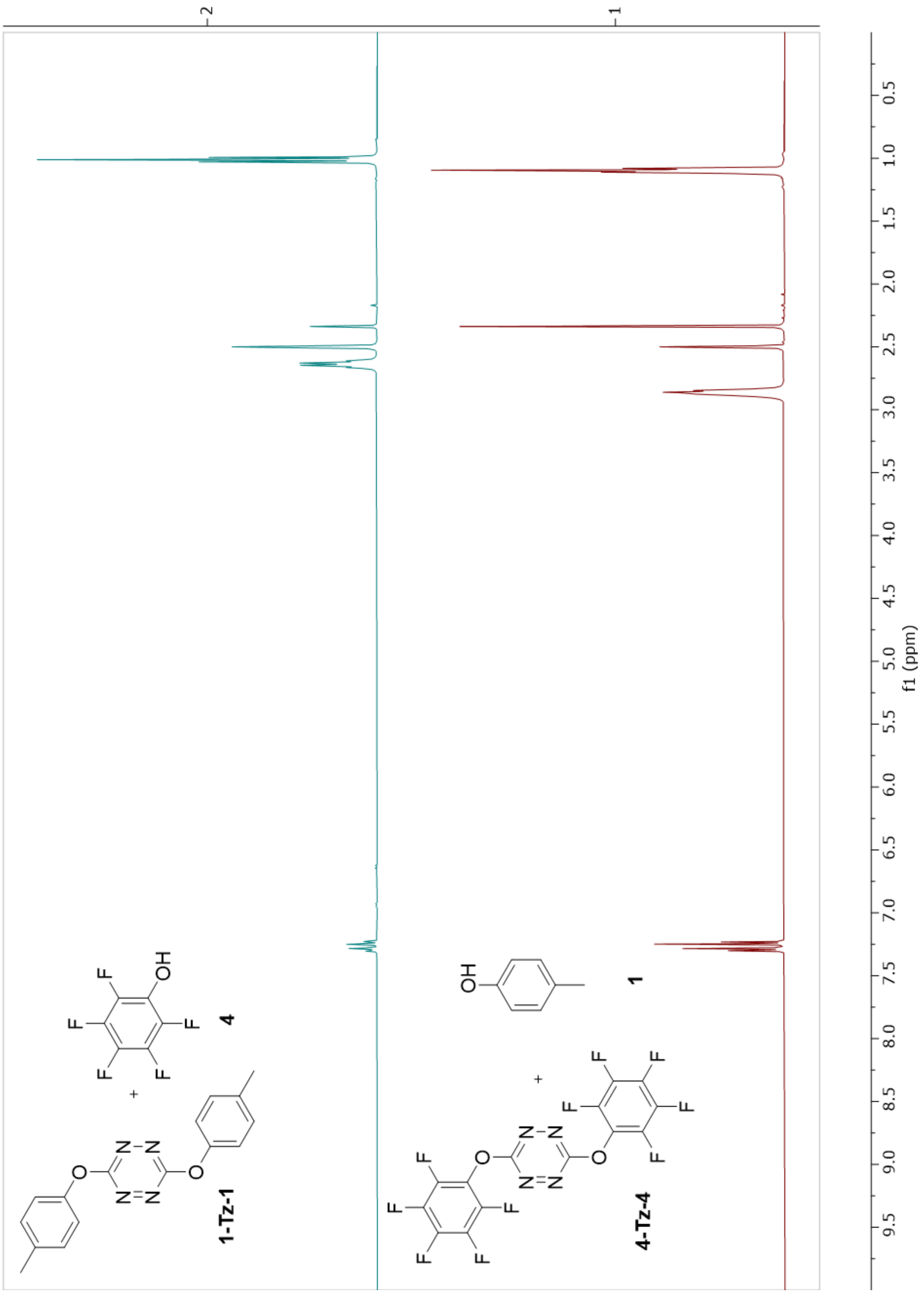

Figure S5

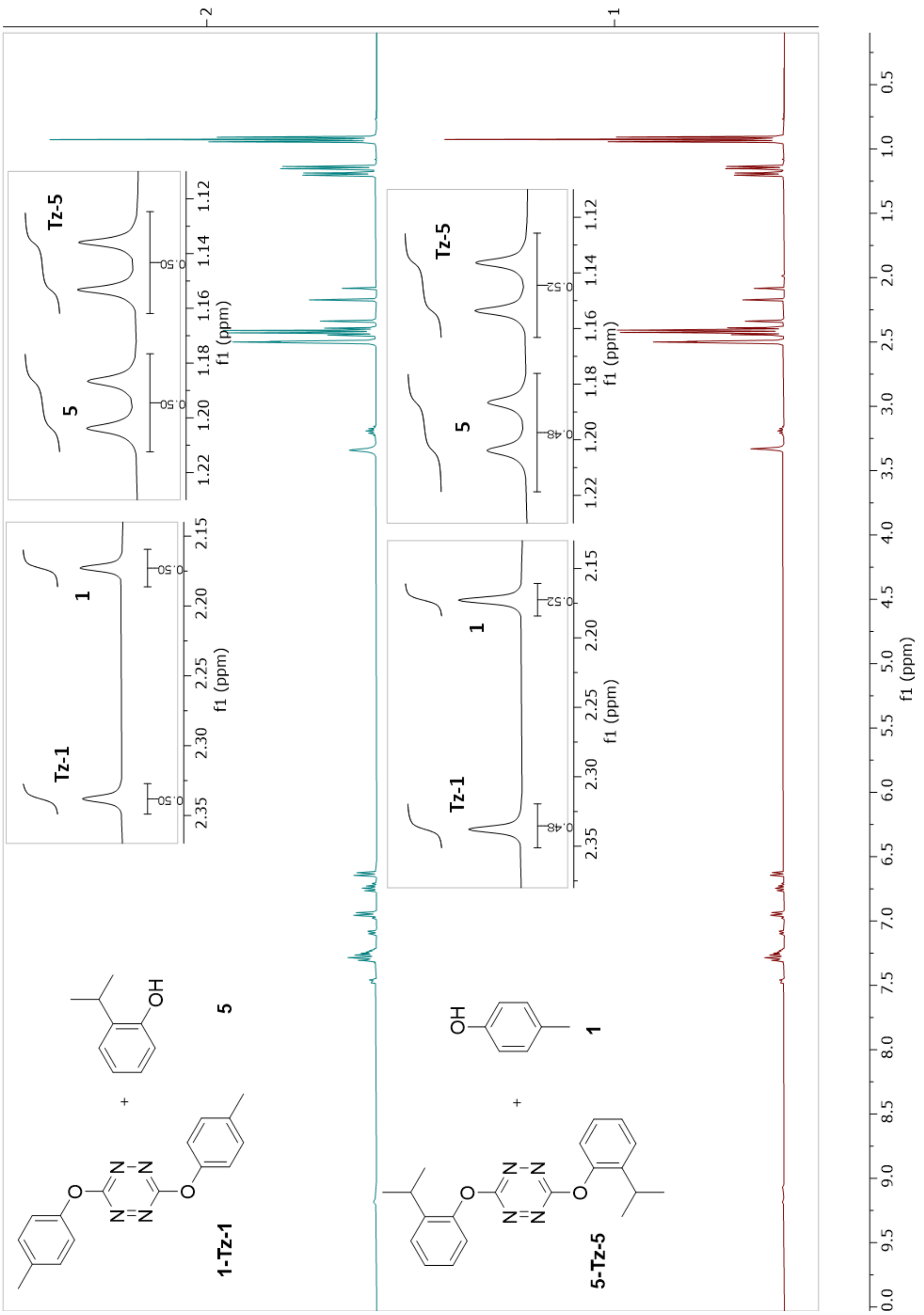

Figure S6

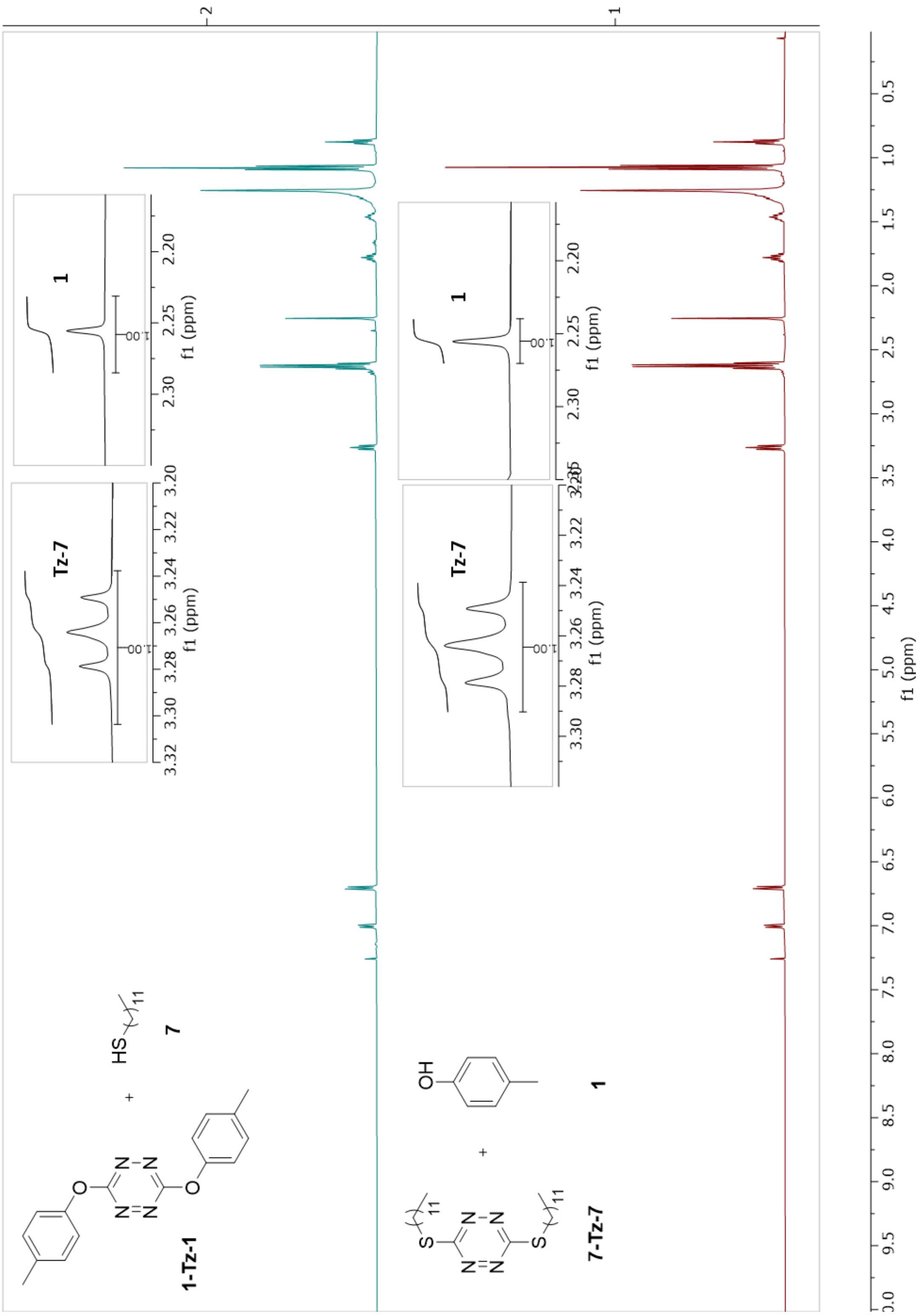

Figure S7

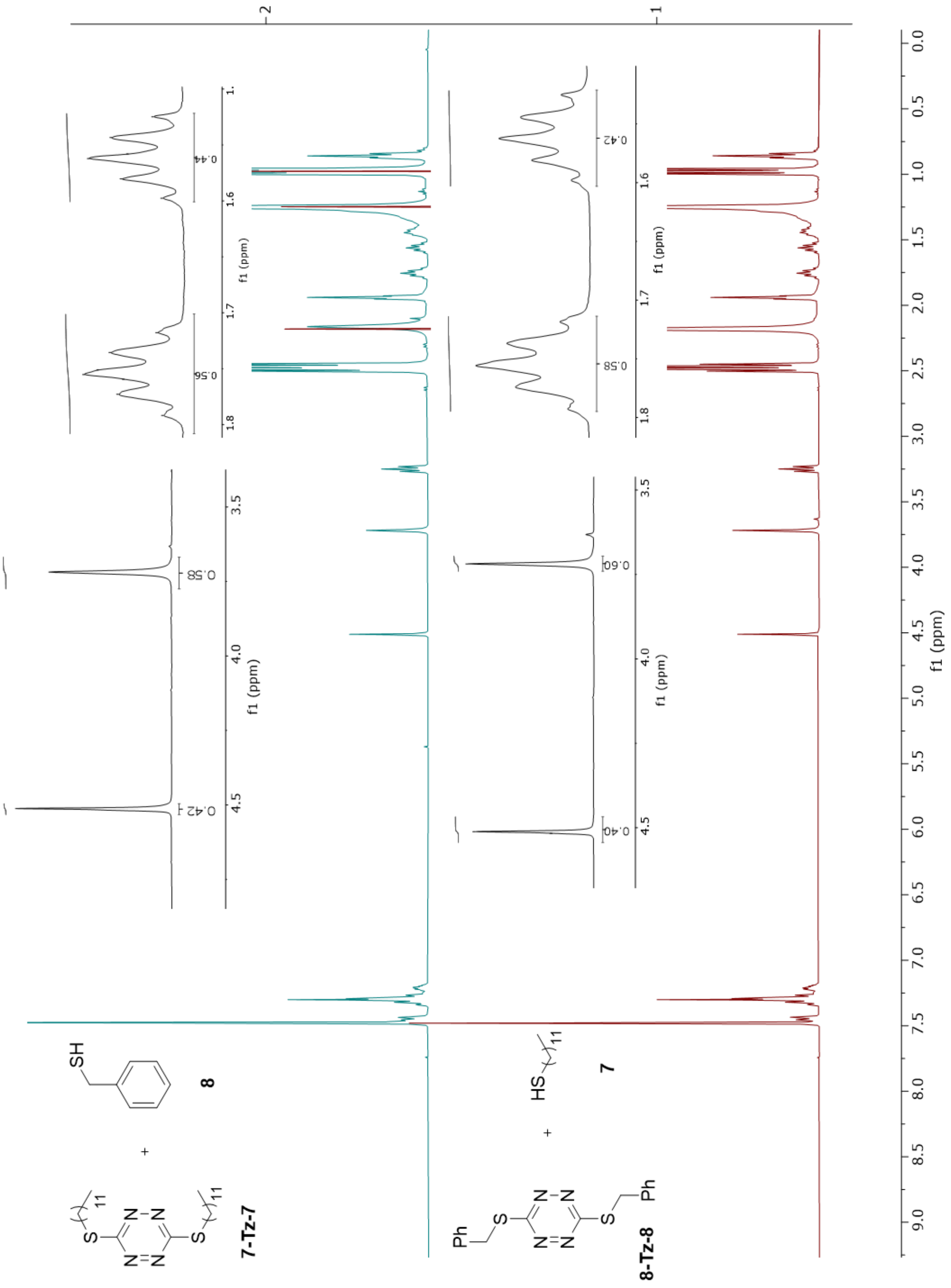

Figure S8

Covalent Dynamic Studies: Quantification and Equilibrium Constants.

Quantification of all the compounds in the reaction mixtures had to be performed most of the times by quantitative ^{13}C NMR because it is the only way to distinguish peaks from the homo and heterodimers, and quantify them. All NMR spectra were recorded at 298 K on a Bruker NEO 500 spectrometer equipped with a CryoProbe^{TM} Prodigy BBO 5mm probe. Quantitative ^{13}C NMR measurements were done using the inverse-gated-decoupling pulse sequence that allows ^{1}H decoupled quantitative ^{13}C NMR spectra to be obtained. Proton decoupling was achieved using the WALTZ65 scheme and used only during the acquisition time, avoiding differential NOE enhancements for each carbon. ^{13}C NMR spectra were acquired using a FID size of 128k, a spectral width of 25KHz, an acquisition time of 2.62s resulting in a FID resolution of 0,38 Hz, 12 μs 90° pulses and a number of transients between 2500 and 3000. Recovery times between 45s to 60s were used. Changes in signal intensities were not observed using longer delays.
NMR spectra was processed and analyzed using Bruker Topspin 4.1 and MNova software. An exponential apodisation function of 0.2 Hz was applied to the FIDs prior to Fourier transformation, followed by baseline correction. Peak areas were calculated using line-fitting to a Lorentz-Gauss functions using routines incorporated in the previously mentioned software.

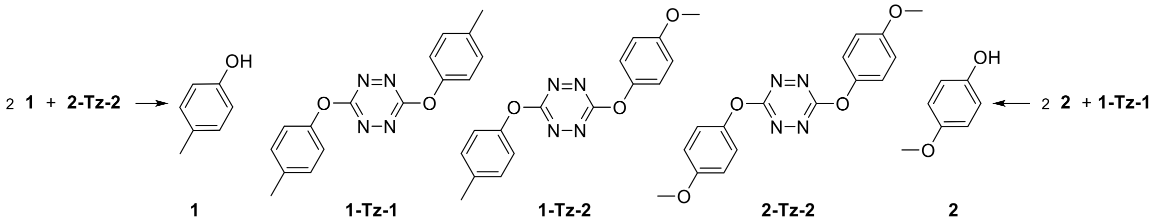

1 1-Tz-1 1-Tz-2 2-Tz-2 2

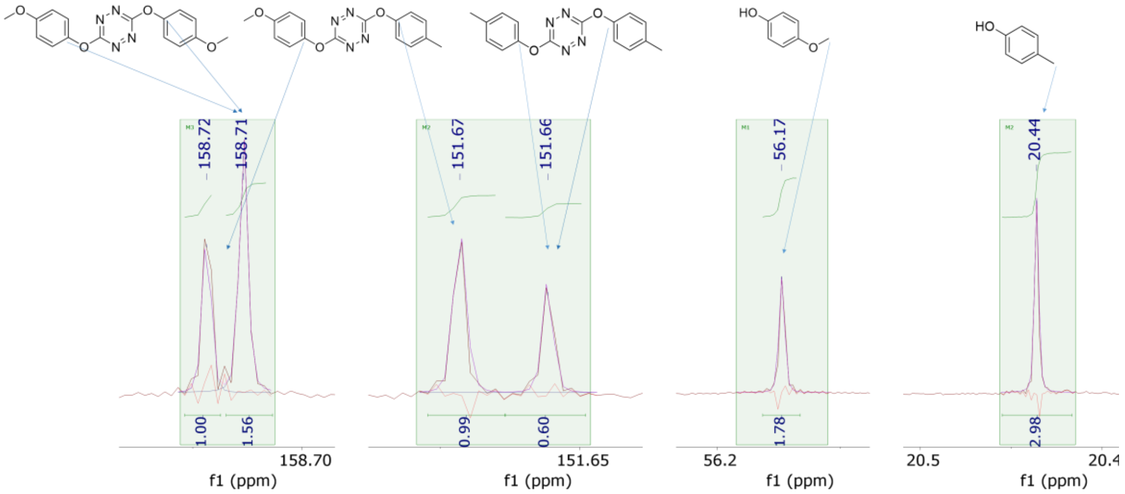

In CD_3CN

	Compound	ppm	Intensity	Width	Area	Relative Area	Molar Fraction
1	**1-Tz-2**	158.72	456742.6	0.32	877567.73	877567.73	0.135
2	**2-Tz-2**	158.71	793474.6	0.30	1516147.79	758073.895	0.108
3	**1-Tz-2**	151.67	481824.3	0.36	1031188.49	1031188.49	0.135
4	**1-Tz-1**	151.66	339595.7	0.29	612668.42	306334.21	0.043
5	**2**	56.17	826983.8	0.35	1811972.72	1811972.72	0.257
6	**1**	20.44	1395967.5	0.35	3220554.45	3220554.45	0.457

Relative area is the area of a peak divided by the number of equivalent carbons. In the case of **1-Tz-2** we took the average of both peaks

$$2\,\mathbf{1} + \mathbf{2\text{-}Tz\text{-}2} \overset{K_1}{\rightleftharpoons} \mathbf{1} + \mathbf{1\text{-}Tz\text{-}2} + \mathbf{2} \overset{K_2}{\rightleftharpoons} 2\,\mathbf{2} + \mathbf{1\text{-}Tz\text{-}1}$$

$$K_1 = \frac{[2][1-\mathrm{Tz}-2]}{[1][2-\mathrm{Tz}-2]} = 0.70 \qquad K_2 = \frac{[2][1-\mathrm{Tz}-1]}{[1][1-\mathrm{Tz}-2]} = 0.18$$

In DMSO-d6

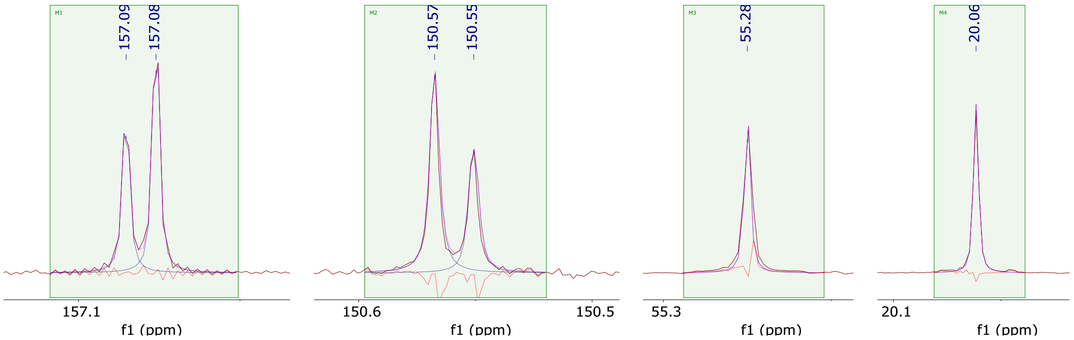

	Compound	ppm	Intensity	Width	Area	Relative Area	Molar Fraction
1	**1-Tz-2**	157.09	818836.6	0.25	1696841.24	1696841.24	0.156
2	**2-Tz-2**	157.08	1491896.7	0.20	2359884.26	1179942.13	0.111
3	**1-Tz-2**	150.57	411232.8	0.49	1628626.73	1628626.73	0.156
4	**1-Tz-1**	150.55	249117.8	0.52	1060491.24	530245.62	0.050
5	**2**	55.28	1200024.6	0.27	2419548.70	2419548.70	0.227
6	**1**	20.06	1782126.0	0.38	4855727.86	4855727.86	0.456

$$K_1 = \frac{[2][1-\mathrm{Tz}-2]}{[1][2-\mathrm{Tz}-2]} = 0.70 \qquad K_2 = \frac{[2][1-\mathrm{Tz}-1]}{[1][1-\mathrm{Tz}-2]} = 0.16$$

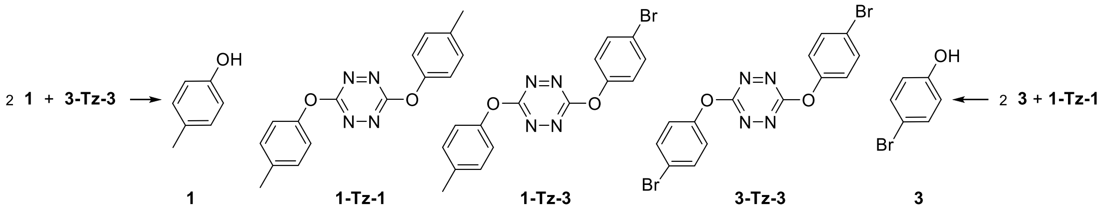

In CD_3CN

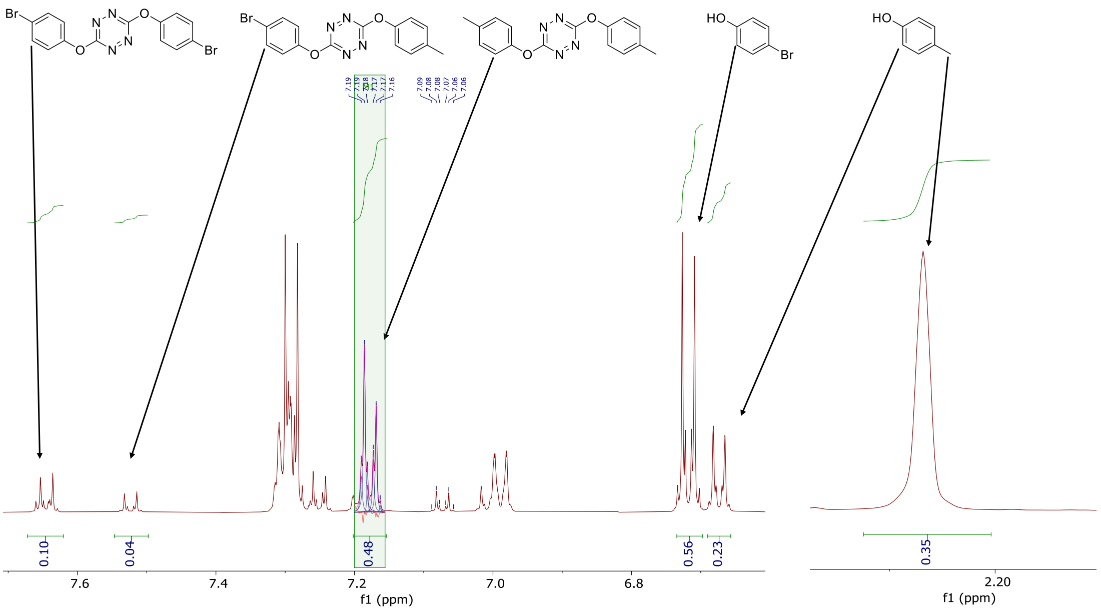

	Compound	ppm	Area	Relative Area	Molar Fraction
1	**3-Tz-3**	7.65	0.10	0.025	0.045
2	**1-Tz-3**	7.52	0.04	0.02	0.036
3	**1-Tz-1**	7.18	0.48	0.12	0.214
4	**3**	6.72	0.56	0.28	0.500
5	**1**	6.68	0.23	0.115	0.205

$$2\,\mathbf{1} + \mathbf{3\text{-}Tz\text{-}3} \underset{}{\overset{K_1}{\rightleftharpoons}} \mathbf{1} + \mathbf{1\text{-}Tz\text{-}3} + \mathbf{3} \overset{K_2}{\rightleftharpoons} 2\,\mathbf{3} + \mathbf{1\text{-}Tz\text{-}1}$$

$$K_1 = \frac{[3][1-\mathrm{Tz}-3]}{[1][3-\mathrm{Tz}-3]} = 1.95 \qquad K_2 = \frac{[3][1-\mathrm{Tz}-1]}{[1][1-\mathrm{Tz}-3]} = 14.50$$

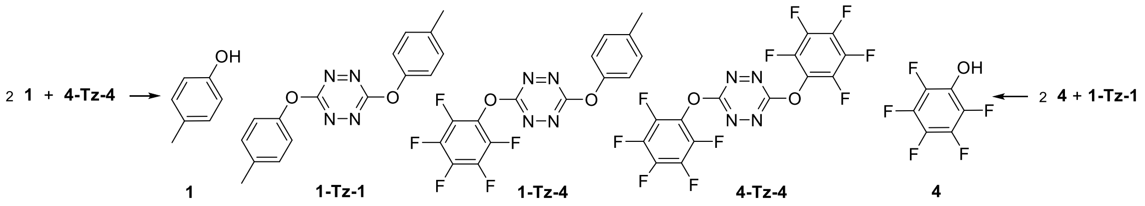

This equilibrium is completely shifted towards the formation of **1-Tz-1** and **4** (molar fractions 0.333 and 0.667 respectively). Therefore it can be considered an irreversible reaction.

2 **1** + **4-Tz-4** ⟶ **1** + **1-Tz-4** + **4** ⟶ 2 **4** + **1-Tz-1**

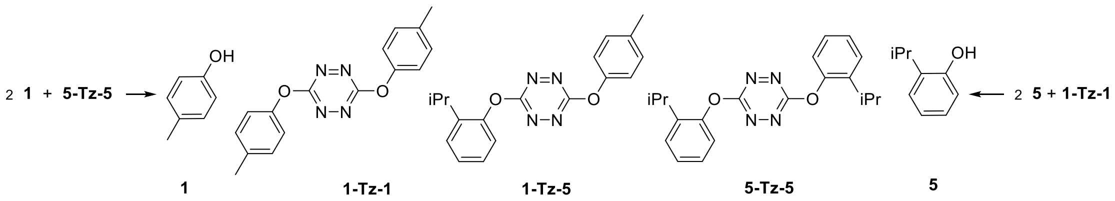

In CD_3CN

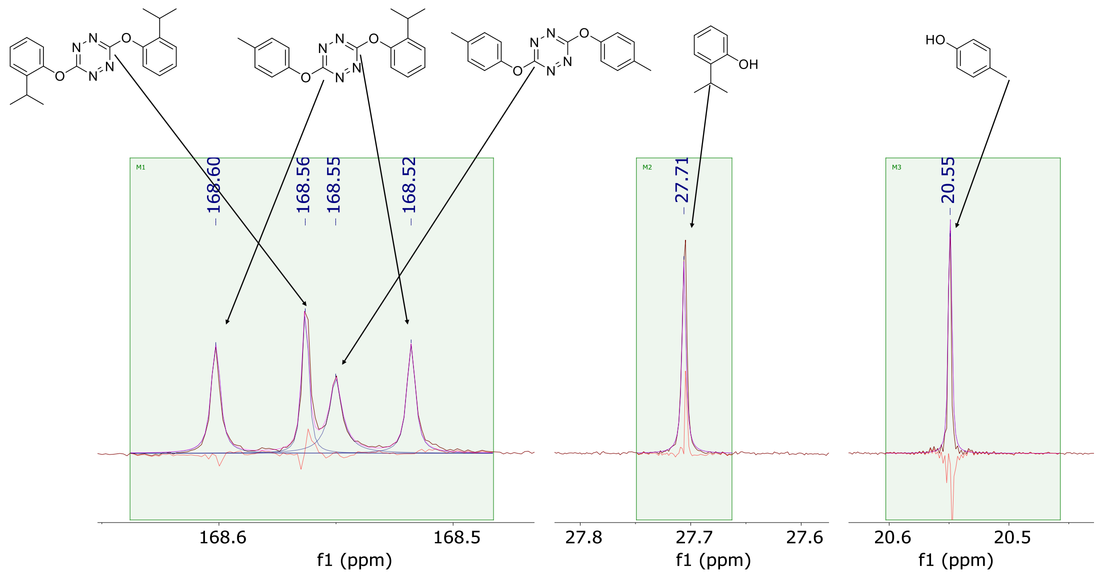

	Compound	ppm	Intensity	Width	Area	Relative Area	Molar Fraction
1	**1-Tz-5**	168.60	352837.7	0.54	1498670.82	1498670.82	0.130
2	**5-Tz-5**	168.56	526732.7	0.56	1917844.04	958922.02	0.081
3	**1-Tz-1**	168.55	252016.1	0.82	1683724.36	841862.18	0.071
4	**1-Tz-5**	168.52	354912.8	0.54	1564539.72	1564539.72	0.130
5	**5**	27.71	757859.2	0.55	3426675.85	3426675.85	0.290
6	**1**	20.55	1588539.5	0.46	5033483.52	5033483.52	0.427

$$2\,\mathbf{1} + \mathbf{5\text{-}Tz\text{-}5} \overset{K_1}{\rightleftharpoons} \mathbf{1} + \mathbf{1\text{-}Tz\text{-}5} + \mathbf{5} \overset{K_2}{\rightleftharpoons} 2\,\mathbf{5} + \mathbf{1\text{-}Tz\text{-}1}$$

$$K_1 = \frac{[5][1-\mathrm{Tz}-5]}{[1][5-\mathrm{Tz}-5]} = 1.09 \qquad K_2 = \frac{[5][1-\mathrm{Tz}-1]}{[1][1-\mathrm{Tz}-5]} = 0.37$$

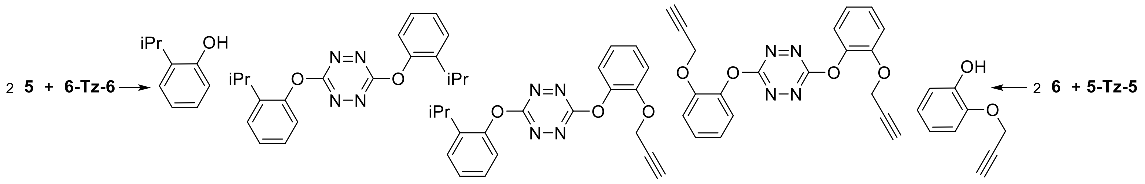

5 **5-Tz-5** **5-Tz-6** **6-Tz-6** **6**

In CD_3CN

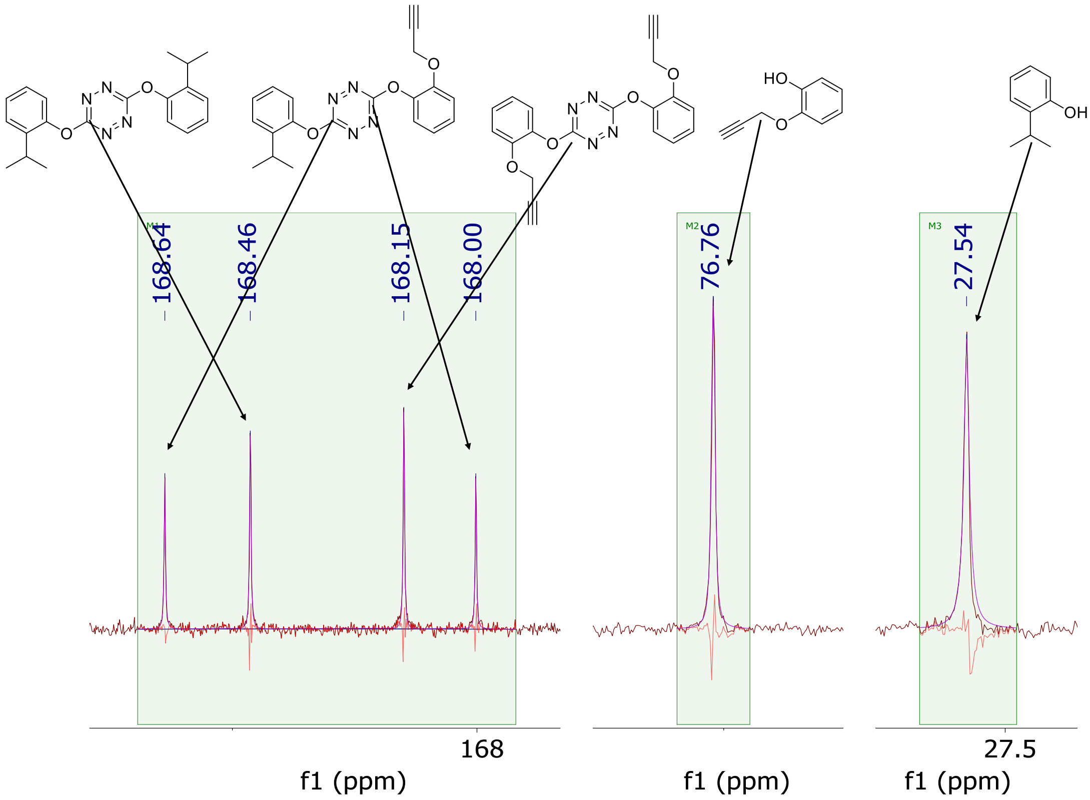

	Compound	ppm	Intensity	Width	Area	Relative Area	Molar Fraction
1	**5-Tz-6**	168.64	177989.6	0.41	590979.08	590979.08	0.116
2	**5-Tz-5**	168.46	227737.9	0.47	811772.13	405886.065	0.081
3	**6-Tz-6**	168.15	257797.9	0.47	908966.50	454483.25	0.090
4	**5-Tz-6**	168.00	179029.3	0.41	580681.66	580681.66	0.116
5	**6**	76.76	390641.0	0.58	1697827.76	1697827.76	0.337
6	**5**	27.54	289626.7	0.74	1894189.30	1894189.30	0.376

$$2\ \mathbf{5} + \mathbf{6\text{-}Tz\text{-}6} \overset{K_1}{\rightleftharpoons} \mathbf{5} + \mathbf{5\text{-}Tz\text{-}6} + \mathbf{6} \overset{K_2}{\rightleftharpoons} 2\ \mathbf{6} + \mathbf{5\text{-}Tz\text{-}5}$$

$$K_1 = \frac{[6][5 - \mathrm{Tz} - 6]}{[5][6 - \mathrm{Tz} - 6]} = 1.16 \quad K_2 = \frac{[6][5 - \mathrm{Tz} - 5]}{[5][5 - \mathrm{Tz} - 6]} = 0.63$$

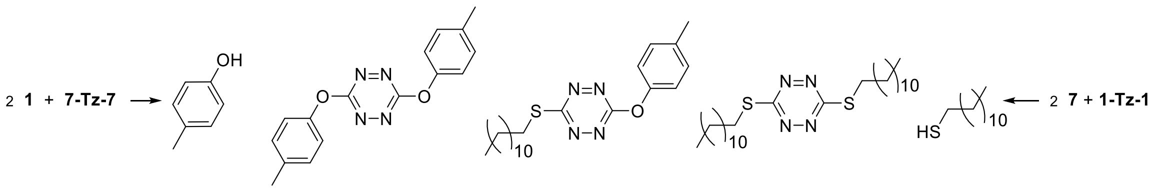

1 1-Tz-1 1-Tz-7 7-Tz-7 7

This equilibrium is completely shifted towards the formation of **7-Tz-7** and **1** (molar fractions 0.333 and 0.667 respectively). Therefore it can be considered an irreversible reaction.

2 **1** + **7-Tz-7** ⟶ **1** + **1-Tz-7** + **7** ⟶ 2 **7** + **1-Tz-1**

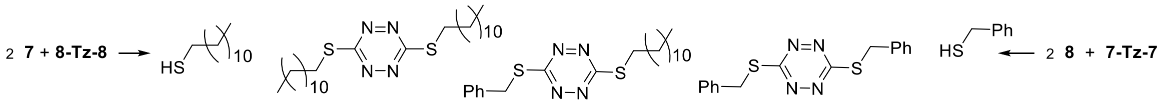

7 **7-Tz-7** **7-Tz-8** **8-Tz-8** **8**

In $CDCl_3/CD_3CN$ (1:1)

This reactions degrades progressively and therefore it is not possible to quantify the compounds by q-CNMR because it requires long times of acquisition. Thus we can only provide the ratio of free thiol versus thiol attached-to-tetrazine shown in *Figure S8*. In the latter case, the homo- and hetero-dimer were considered together and called **Tz-7** or **Tz-8**. Thus, the tendency of a thiol to be released from the tetrazine or to remain attached to it can be easily examined.

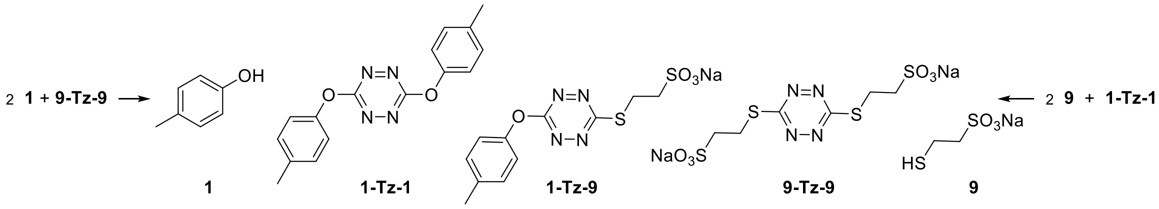

In D_2O (0.1 M Na_2CO_3)/CD_3CN (8:2)

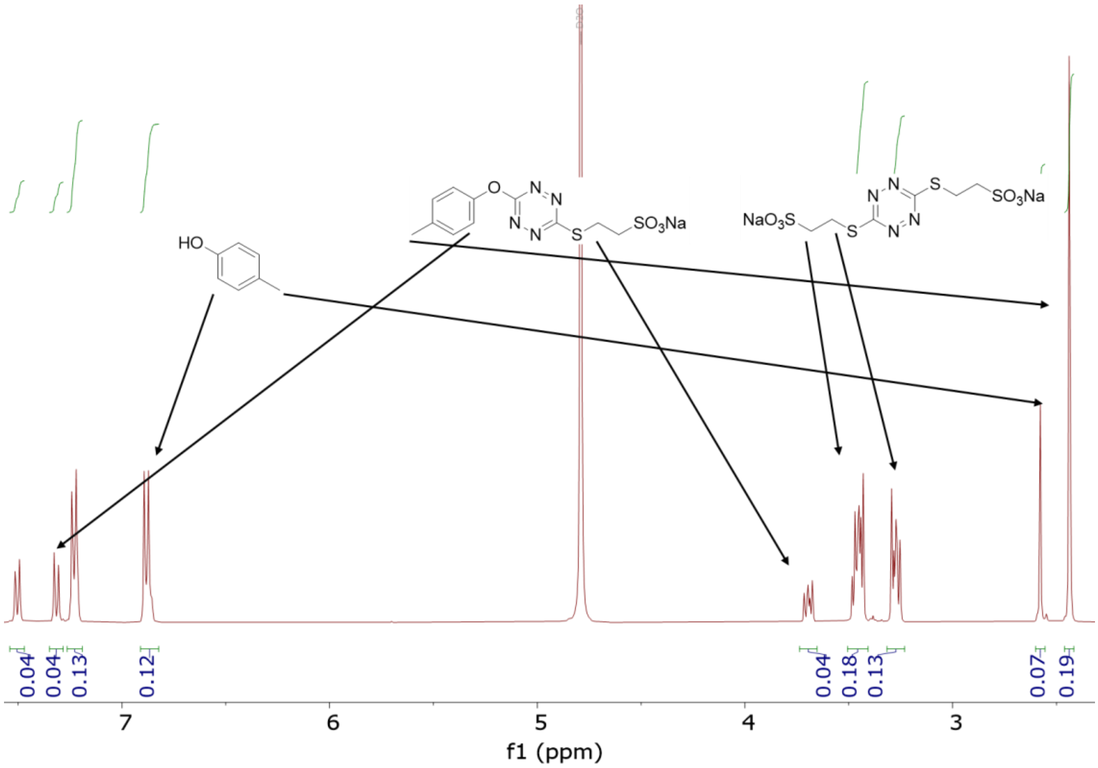

	Compound	ppm	Area	Relative Area	Molar Fraction
1	**1-Tz-9**	7.31	0.04	0.02	0.170
2	**1**	7.23	0.13	0.065	0.553
3	**9-Tz-9**	3.27	0.13	0.0325	0.277
4	**1-Tz-1**	-	0	0	0
5	**9**	-	0	0	0

Table S1. Summary of the quantification of different equilibria and their constants

[a]

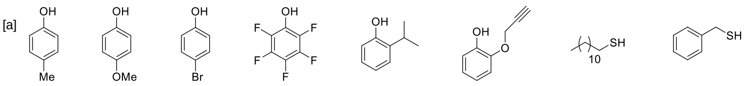

1 2 3 4 5 6 7 8

[b] 2 **A** + **B-Tz-B** $\underset{}{\overset{K_1}{\rightleftharpoons}}$ **A** + **A-Tz-B** + **B** $\underset{}{\overset{K_2}{\rightleftharpoons}}$ 2 **B** + **A-Tz-A**

$$K_1 = \frac{[\mathrm{B}][\mathrm{A-Tz-B}]}{[\mathrm{A}][\mathrm{B-Tz-B}]} \qquad K_2 = \frac{[\mathrm{B}][\mathrm{A-Tz-A}]}{[\mathrm{A}][\mathrm{A-Tz-B}]}$$

Entry

1

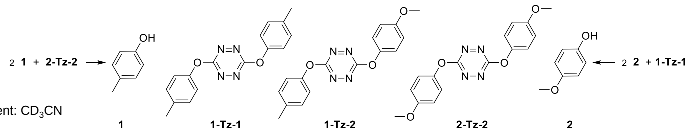

[c] Solvent: CD_3CN

	1	1-Tz-1	1-Tz-2	2-Tz-2	2
**Molar Fraction**	0.457	0.043	0.135	0.108	0.257

$$K_1 = \frac{[2][1-\mathrm{Tz}-2]}{[1][2-\mathrm{Tz}-2]} = 0.70 \qquad K_2 = \frac{[2][1-\mathrm{Tz}-1]}{[1][1-\mathrm{Tz}-2]} = 0.18$$

2

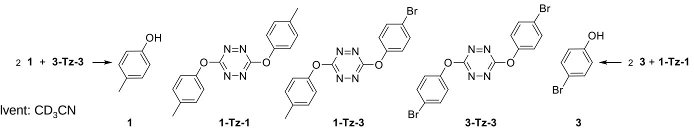

Solvent: CD_3CN

	1	1-Tz-1	1-Tz-3	3-Tz-3	3
**Molar Fraction**	0.205	0.214	0.036	0.045	0.500

$$K_1 = \frac{[3][1-\mathrm{Tz}-3]}{[1][3-\mathrm{Tz}-3]} = 1.95 \qquad K_2 = \frac{[3][1-\mathrm{Tz}-1]}{[1][1-\mathrm{Tz}-3]} = 14.50$$

3

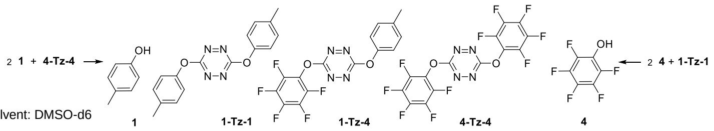

Solvent: DMSO-d6

	1	1-Tz-1	1-Tz-4	4-Tz-4	4
**Molar Fraction**	0	0.333	0	0	0.667

Completely shifted

4

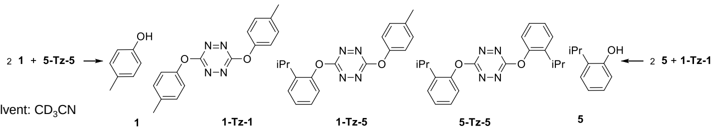

Solvent: CD_3CN

	1	1-Tz-1	1-Tz-5	5-Tz-5	5
**Molar Fraction**	0.427	0.071	0.130	0.081	0.290

$$K_1 = \frac{[5][1-\mathrm{Tz}-5]}{[1][5-\mathrm{Tz}-5]} = 1.09 \qquad K_2 = \frac{[5][1-\mathrm{Tz}-1]}{[1][1-\mathrm{Tz}-5]} = 0.37$$

5

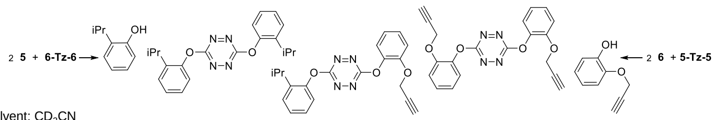

Solvent: CD_3CN

	5	5-Tz-5	5-Tz-6	6-Tz-6	6
**Molar Fraction**	0.337	0.081	0.116	0.090	0.376

$$K_1 = \frac{[6][5-\mathrm{Tz}-6]}{[5][6-\mathrm{Tz}-6]} = 1.25 \qquad K_2 = \frac{[6][5-\mathrm{Tz}-5]}{[5][5-\mathrm{Tz}-6]} = 0.75$$

6

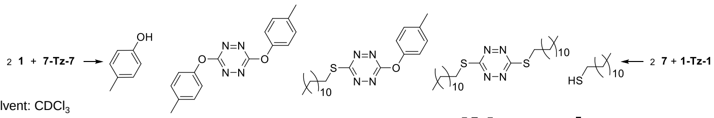

Solvent: $CDCl_3$

	1	1-Tz-1	1-Tz-7	7-Tz-7	7
**Molar Fraction**	0.667	0	0	0.333	0

Completely shifted

7

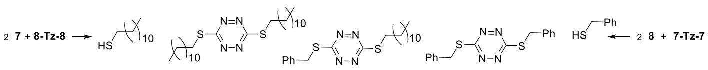

Solvent: $CD_3CN/CDCl_3$ (1:1)

	7	7-Tz-7	7-Tz-8	8-Tz-8	8
**Molar Fraction**	0,210	(7-Tz-7+7-Tz8) 0,290	(7-Tz-7+7-Tz8) 0,200		0,300

K_1 = not determined K_2 = not determined

[a] Nucleophiles employed. [b] Schematic equilibria and their corresponding constants. [c] All reactions were carried out in 0.5 ml of the specified solvent (chosen due to solubility and kinetic reasons), and 0.02 mmol of the corresponding phenol or thiol, which were adjusted to the correct stoichiometry, prior to the addition of 5 µL of triethylamine. Reactions were followed by 1H NMR until no further changes were observed. Equilibration times range from less than 5 minutes to 24 hours.

Kinetics Studies

Phenol-phenol exchange

General procedure. To a solution of **1-Tz-1** (0.011 mmol) in the corresponding deuterated solvent (0.5 mL) and phenol **2** (0.022 mmol), the corresponding base (0.033 mmol) was added and the reaction mixture was monitored by ^{1}H NMR at the temperature indicated in each figure. The reaction in DMSO-d6 is too fast to be followed by NMR, and the reaction was complete in the first spectrum acquired.

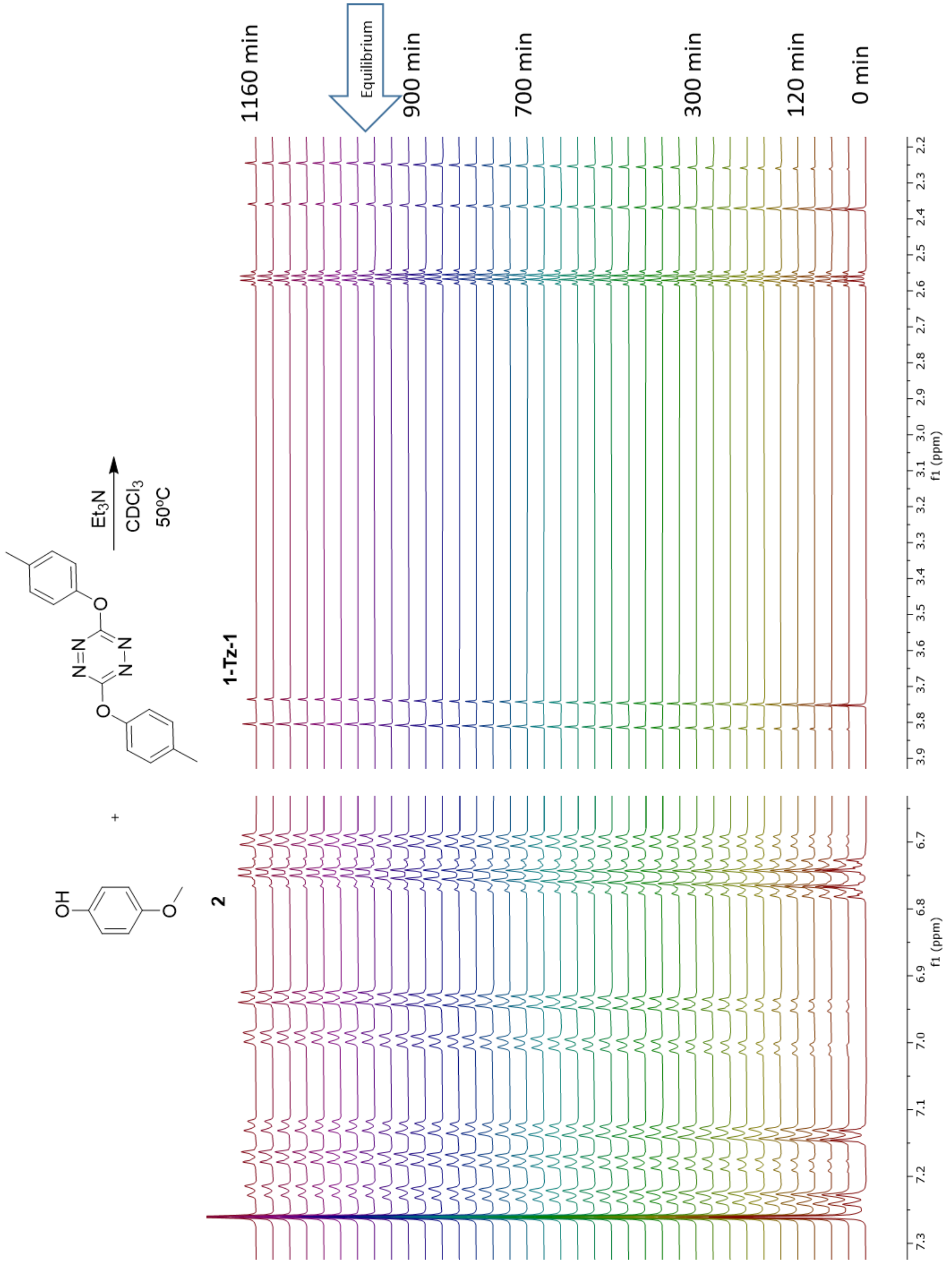

2
1-Tz-1
Et$_3$N
CDCl$_3$
50ºC
Equilibrium
1160 min
900 min
700 min
300 min
120 min
0 min
f1 (ppm)

Figure S9

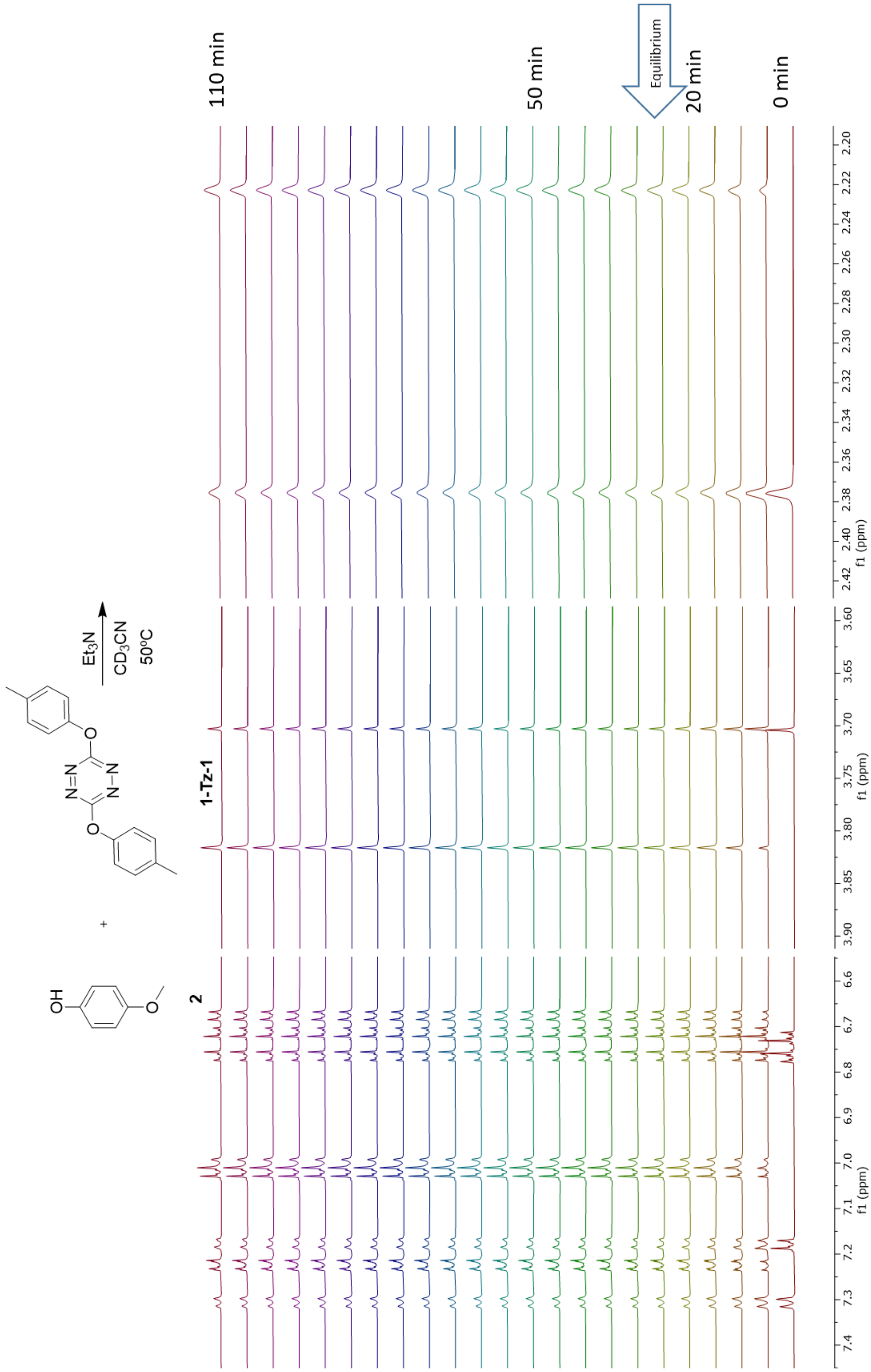

2
1-Tz-1
Et3N
CD3CN
50ºC
110 min
50 min
Equilibrium
20 min
0 min
f1 (ppm)

Figure S10

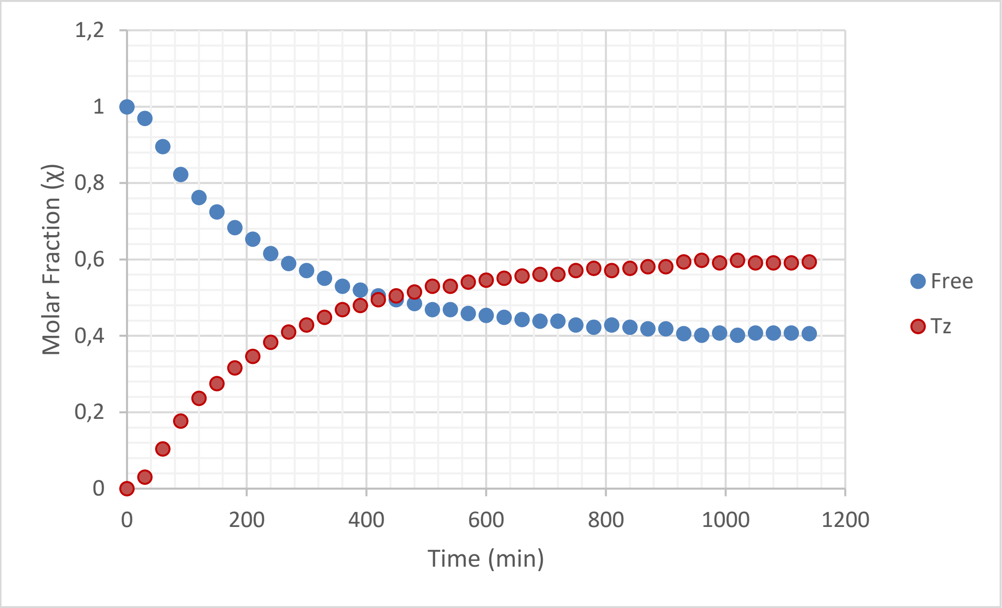

Figure S11. Kinetics of the reaction between ***1-Tz-1*** *and* ***2*** *(reverse direction) in deuterated chloroform at 50°C. Red dots represent the molar fraction of* ***Tz-2****, while blue dots represent the molar fraction of free* ***2****.*

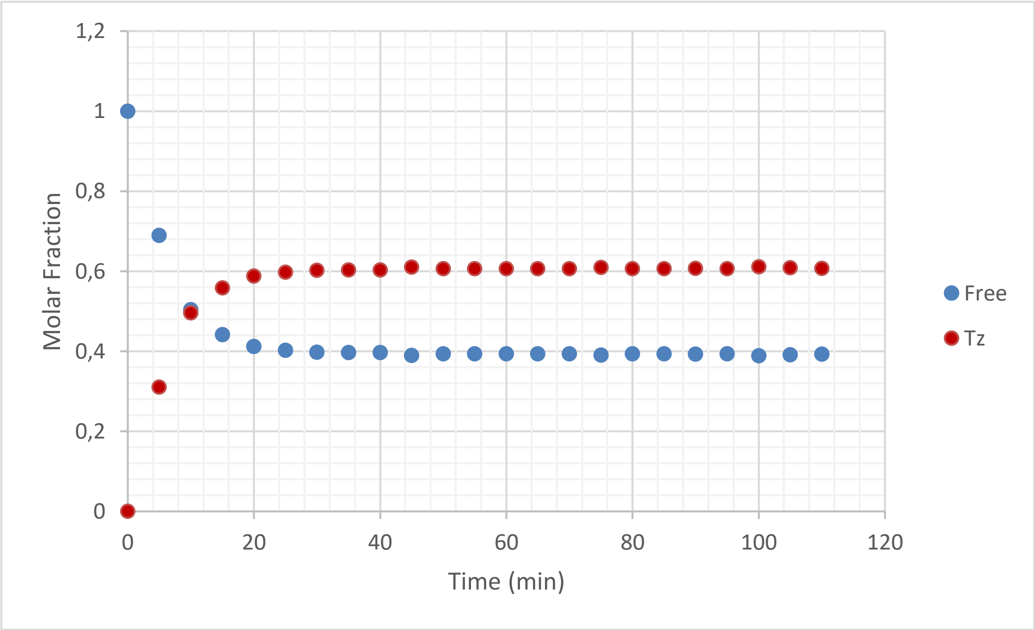

Figure S12. Kinetics of the reaction between ***1-Tz-1*** *and* ***2*** *(reverse) in deuterated acetonitrile at 50°C. Red dots represent the molar fraction of* ***Tz-2****, while blue dots represent the molar fraction of free* ***2****.*

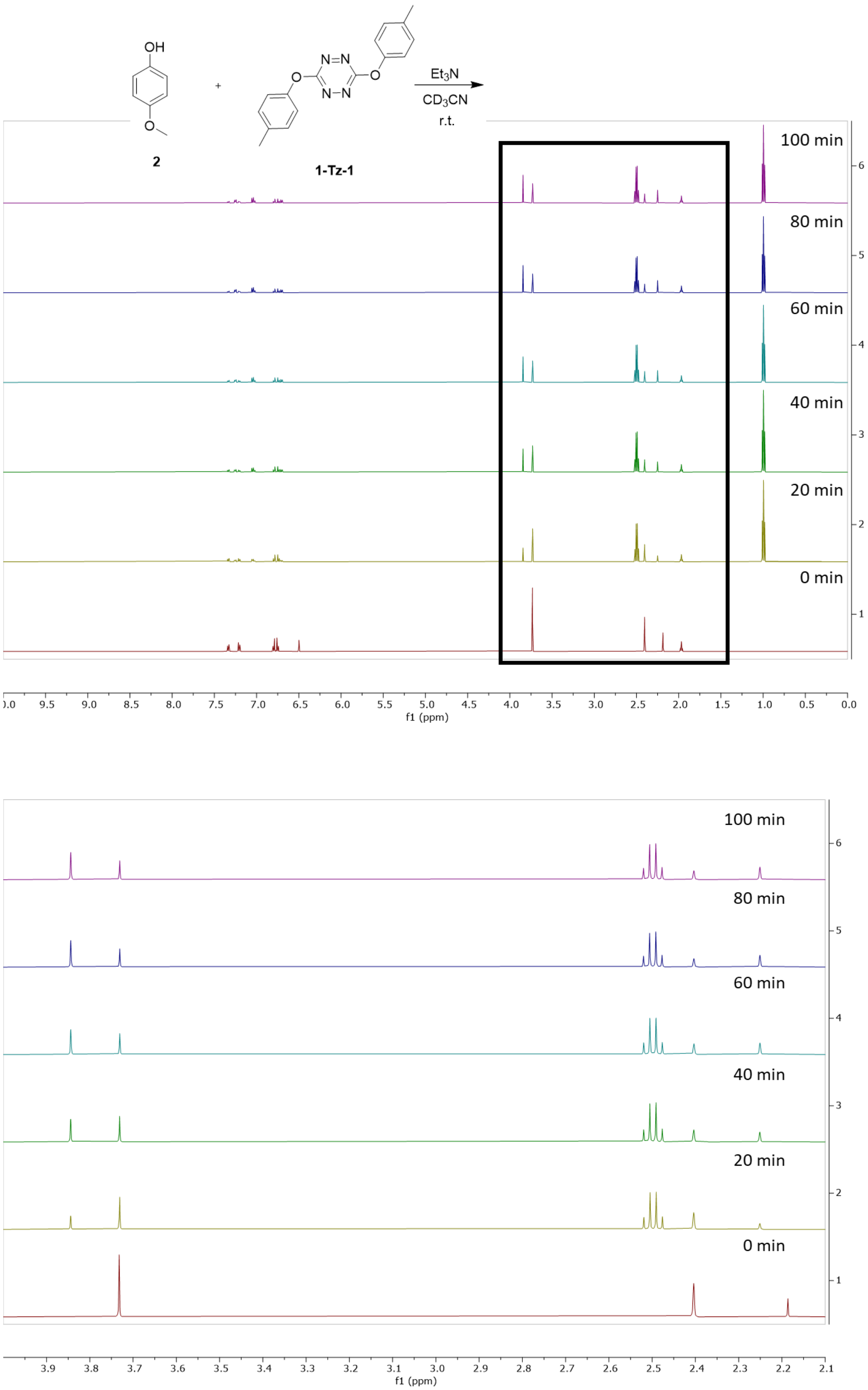
OH
O
2
N=N
N–N
O
O
1-Tz-1
Et3N
CD3CN
r.t.
100 min
80 min
60 min
40 min
20 min
0 min
f1 (ppm)
100 min
80 min
60 min
40 min
20 min
0 min
f1 (ppm)

Figure S13.

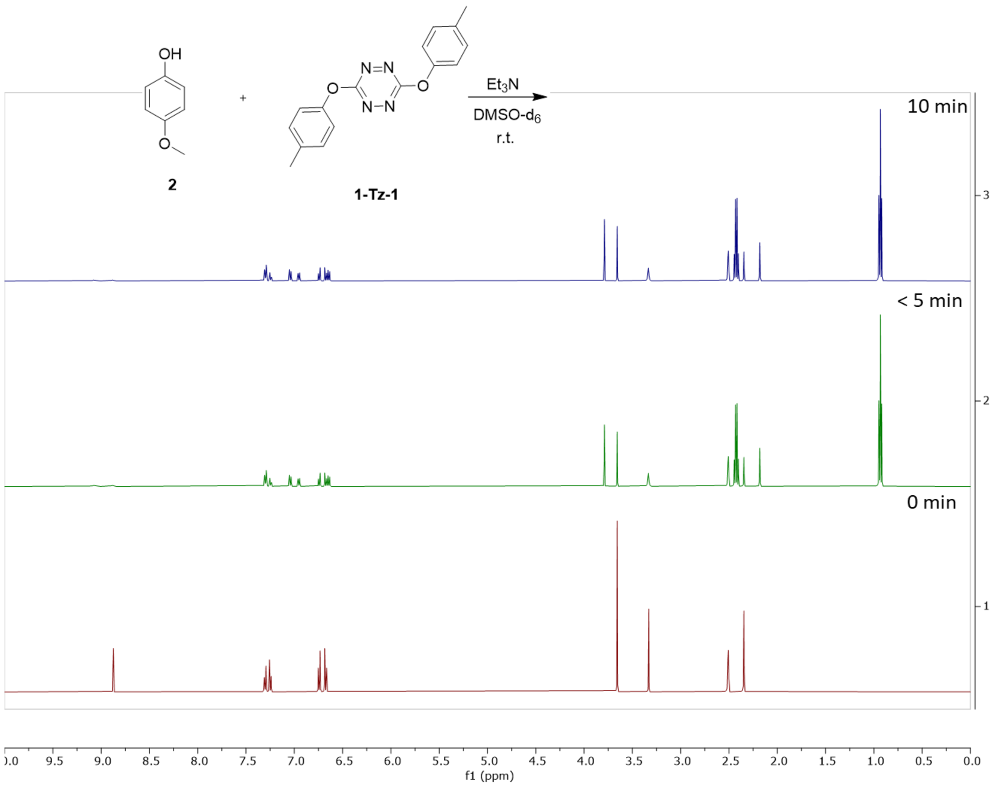

Figure S14.

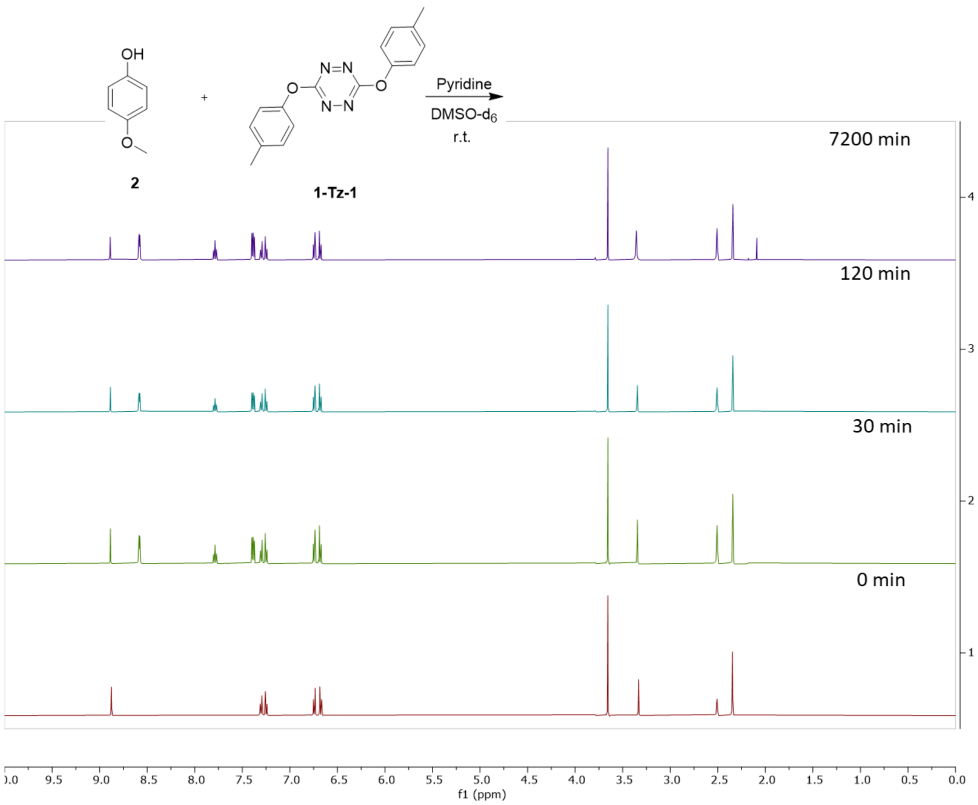

Figure S15.

Phenol-thiol exchange

To a solution of **1-Tz-1** (0.011 mmol) in deuterated chloroform (0.5 mL) and thiol **7** (0.022 mmol), triethylamine (0.033 mmol) was added and the reaction mixture was monitored by ^{1}H NMR at 25ºC.

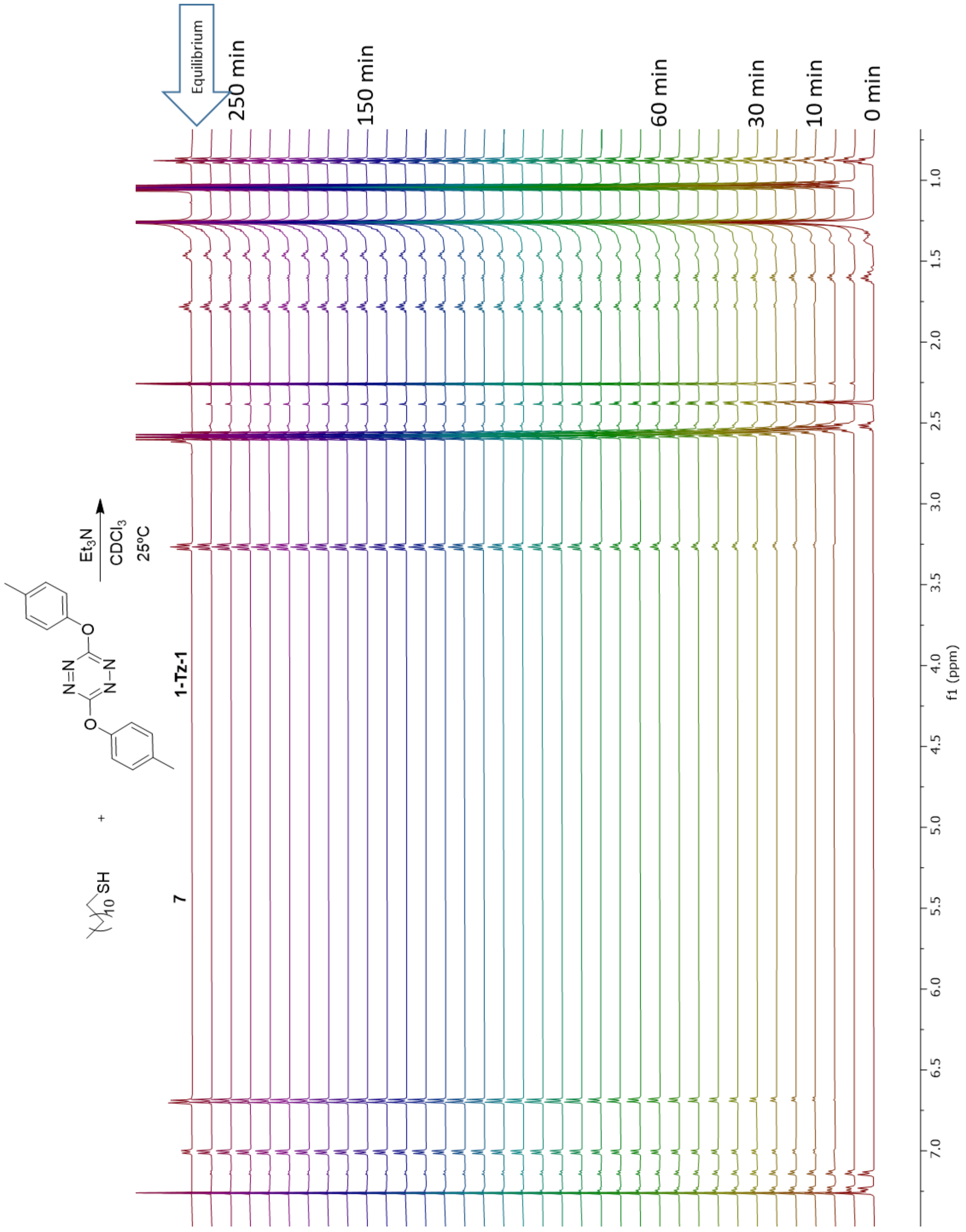

Figure S16.

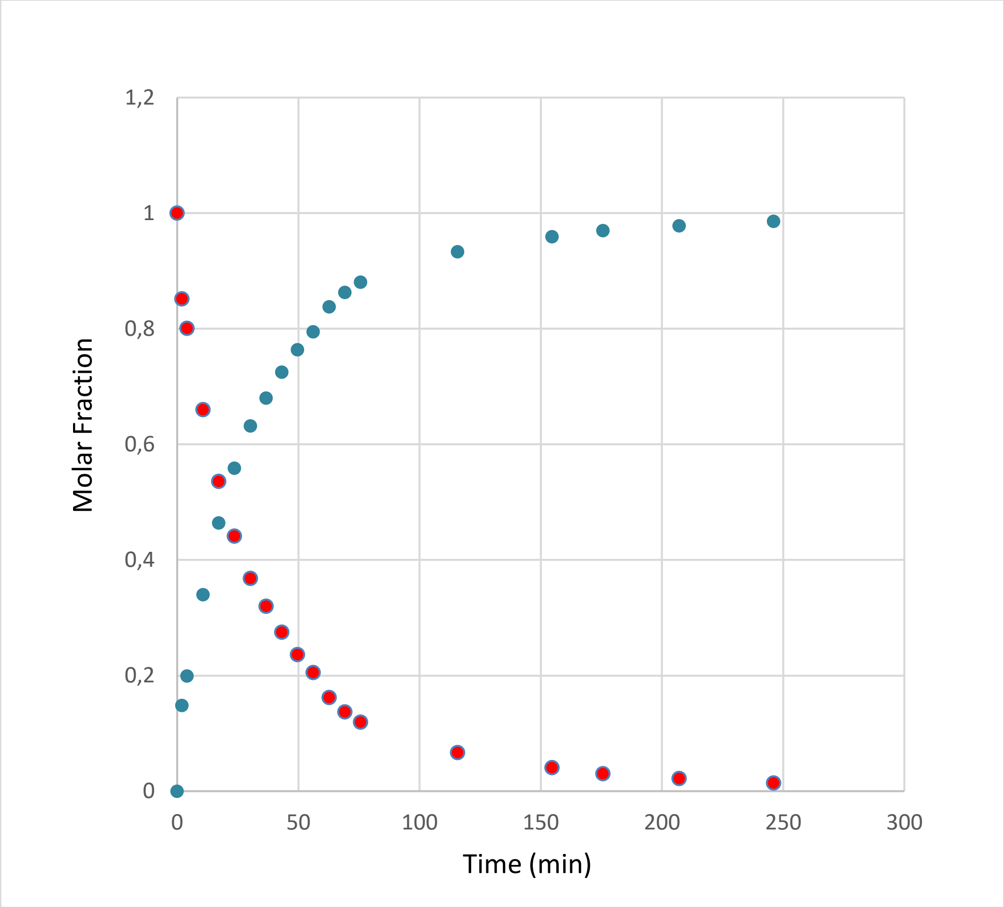

*Figure S17. Kinetics of the reaction between **1-Tz-1** and **7** in deuterated chloroform at 25°C. Red dots represent the molar fraction of **Tz-1**, while blue dots represent the molar fraction of free **1**.*

<u>Thio-thiol exchange</u>

To a solution of **7-Tz-7** (0.011 mmol) and thiol **8** (0.022 mmol), in deuterated chloroform (0.5 mL) or acetonitrile, or a 1:1 mixture of both, triethylamine (0.033 mmol) was added and the reaction mixture was monitored by ^{1}H NMR at 25ºC. The reaction is too fast to be followed by NMR, and the reaction was complete in the first spectrum acquired. It was also detected that a progressive and unknown decomposition of the compounds took place, precluding the quantification by ^{13}C NMR which requires long acquisition times.

<u>Shifting the equilibrium.</u>

In order to further prove the reversible nature of this reaction, we examined if equilibrium could be shifted "uphill", towards the unfavourable direction by increasing the concentration of one of the chemical species in solution. Specifically, once the equilibrium was reached in the reaction between **2** and **1-Tz-1**, which implies that most of **2** is attached to the tetrazine ring, we added 10 more equivalents of cresol **1**, in order to force the release of free phenol **2**. It is clearly seen by NMR that the compounds **Tz-2** (homo and heterodimer) are greatly minimized and the ratio **2:Tz-2** is completely inverted.

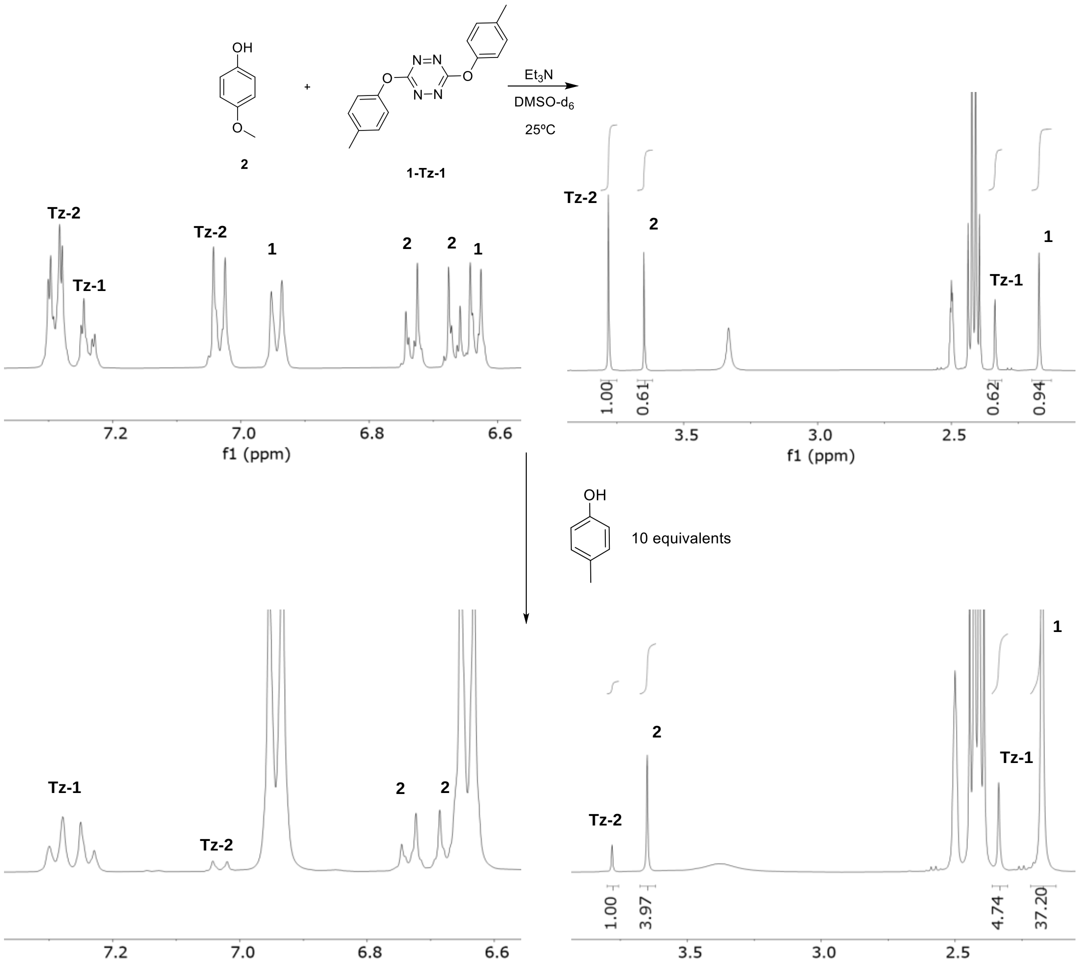

Figure S18.

Run&Stop experiment procedure.

To a solution of the tetrazine **1-Tz-1** (0.011 mmol) in CD_3CN (0.5 mL), phenol **2** (0.022 mmol) and Et_3N (0.033 mmol, 5 µL) was added and the reaction mixture was monitored by ^{1}H NMR at 25ºC. After 20 minutes, TFA (0.034 mmol) was added and the reaction mixture was monitored by ^{1}H-NMR at 25ºC. After 20 minutes, Et_3N (0.070 mmol, 9.8 µL) was added and the reaction mixture was monitored by ^{1}H NMR at 25ºC.

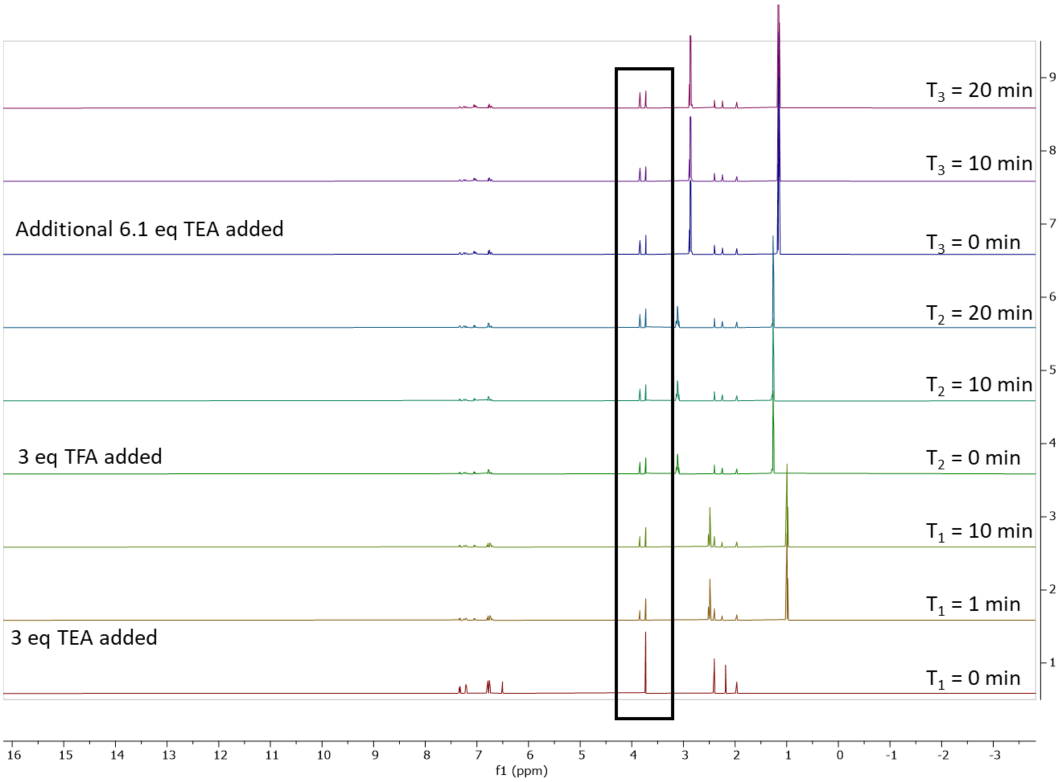

Figure S19.

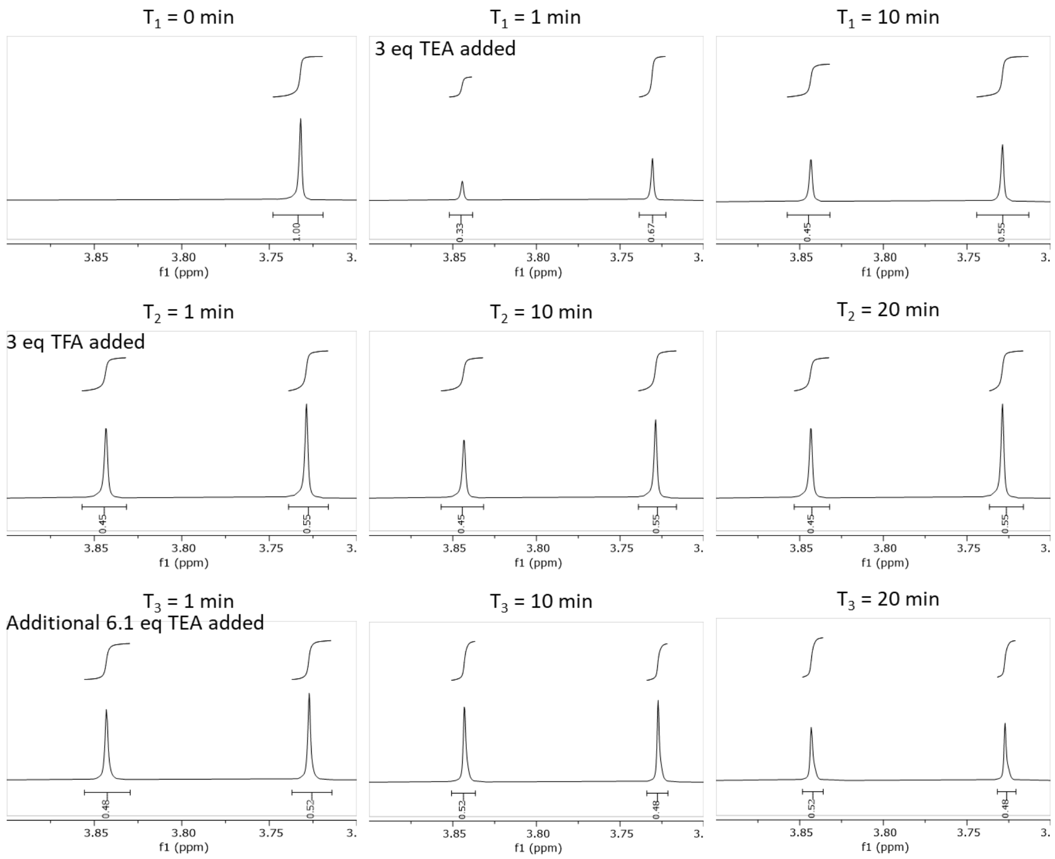

Figure S20.

<u>Photolysis Studies</u>

General procedure. 0.5 mL of a 0.01M solution of the corresponding tetrazine is added to a quartz NMR tube. The solution is exposed to UV light with an ACE-Hanovia photochemical lamp 7830-60 (450W) and monitored by ^{1}H NMR experiment.

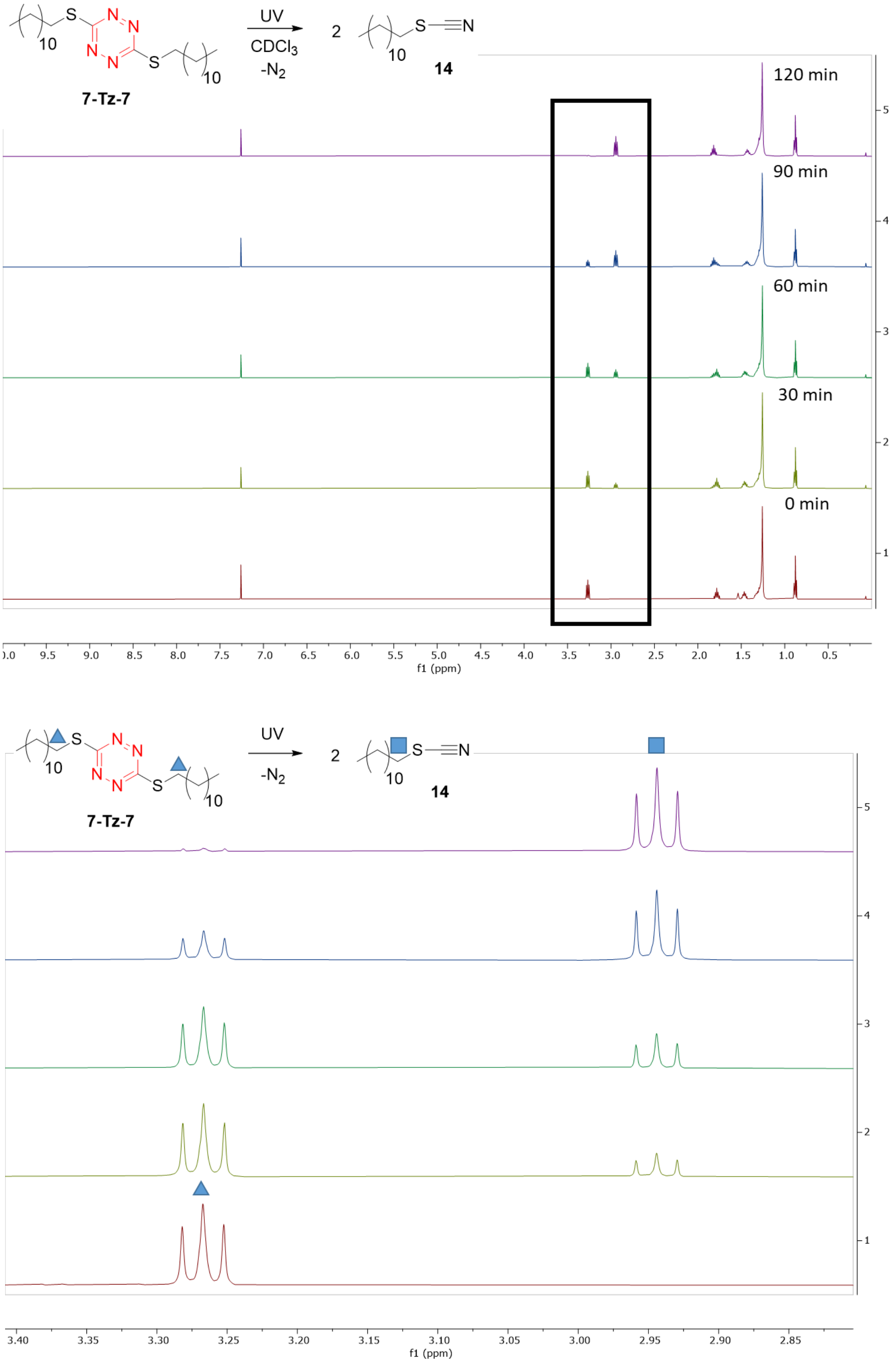

Figure S21.

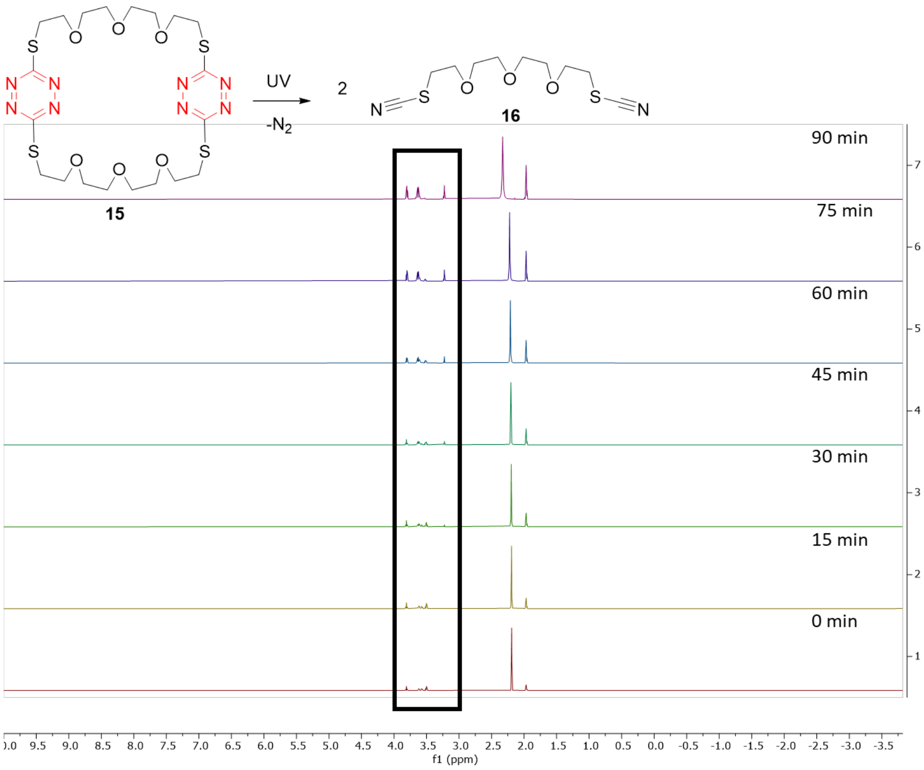
UV
-N2
2
15
16
90 min
75 min
60 min
45 min
30 min
15 min
0 min
f1 (ppm)

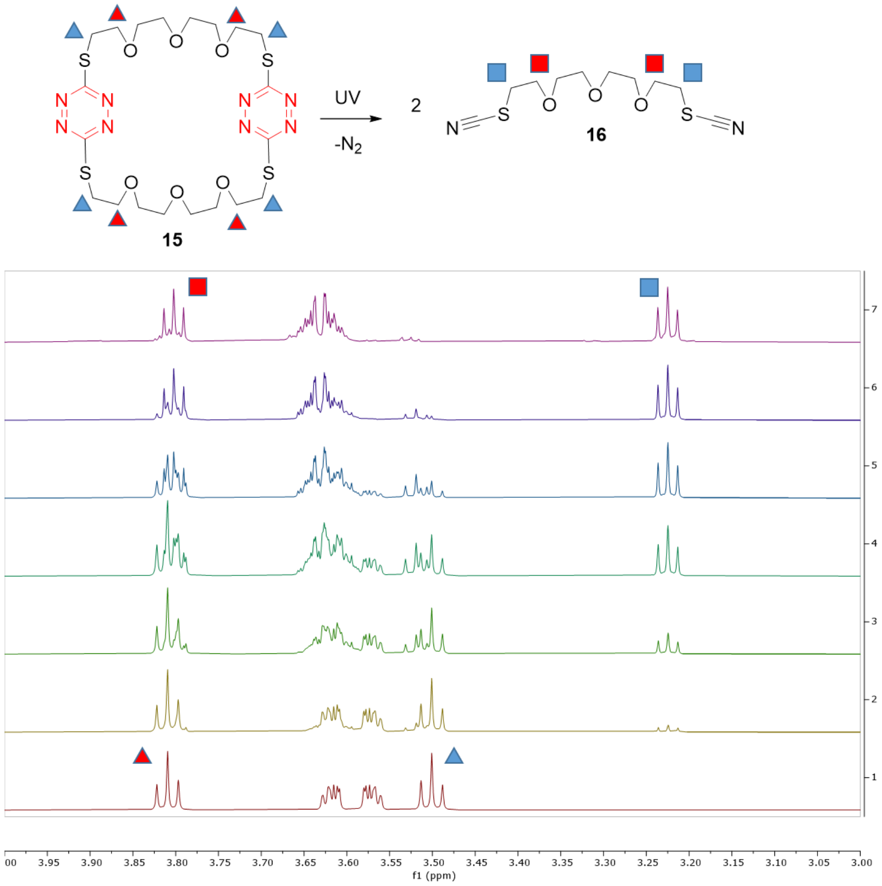
UV
-N2
2
15
16
f1 (ppm)

Figure S22.

Photolysis Products detected by NMR

14

^{1}H NMR (500 MHz, $CDCl_3$, δ ppm): 2.92 (t, J = 7.3 Hz, 2H, CH_2), 1.79 (p, J = 7.3 Hz, 2H, CH_2), 1.40 (q, J = 7.2 Hz, 2H, CH_2), 1.33-1.20 (m, 16H), 0.85 (t, J = 6.9 Hz, 3H). **^{13}C NMR** (126 MHz, $CDCl_3$, δ ppm): 14.2 (3 CH_3), 22.8 (2 CH_2), 28.1 (2 CH_2), 28.9 (2 CH_2), 29.4 (4CH_2), 29.6 (2CH_2), 30.0 (CH_2), 31.0 (CH_2), 32.0 (CH_2) 34.2 (CH_2), 112.5 (SCN). **HR-MS** (ESI^+, m/z): $[M+Na]^+$= $C_{13}H_{25}NNaS$, calcd.: 250.1605; found 250.1606.

16

^{1}H NMR (500 MHz, CD_3CN, δ ppm): 3.20 (t, J = 5.7 Hz, 4H, CH_2), 3.59 (ddd, J = 6.0, 3.2, 1.4 Hz, 4H), 3.62 (ddd, J = 5.8, 3.1, 1.2 Hz, 4H), 3.77 (t, J = 5.7 Hz, 4H). **^{13}C NMR** (126 MHz, $CDCl_3$, δ ppm): 14.2 (3 CH_3), 22.8 (2 CH_2), 28.1 (2 CH_2), 28.9 (2 CH_2), 29.4 (4CH_2), 29.6 (2CH_2), 30.0 (CH_2), 31.0 (CH_2), 32.0 (CH_2) 34.2 (CH_2), 112.5 (SCN).

Determination of the association constant

Association constant of macrocycle **15** were measured by UV titration and fitting of the isotherms on supramolecular.org. UV titrations were performed by addition of aliquots of Ach PF_6 solution in acetonitrile ($5.81\cdot10^{-3}$ M), to a solution of macrocycle **15** in acetonitrile placed in the UV-quartz cell ($2.5\cdot10^{-4}$ M). Absorbance at three different wavelengths (410, 425 and 525nm) was recorded. Dilution correction was applied. The titration was repeated 2 times.

Guest	Ka (M^{-1})	URL
ACh PF_6	102.69 M^{-1} ± 1.7558 %	http://app.supramolecular.org/bindfit/view/bb2292b2-d417-4490-9218-68796c03c967
	114.61 M^{-1} ± 1.9029 %	http://app.supramolecular.org/bindfit/view/bcf15556-6a7b-411d-a284-39e6cfada62a

Unfortunately, no changes upon addition of ACh PF_6 to macrocycle **15** were observed in the NMR spectra

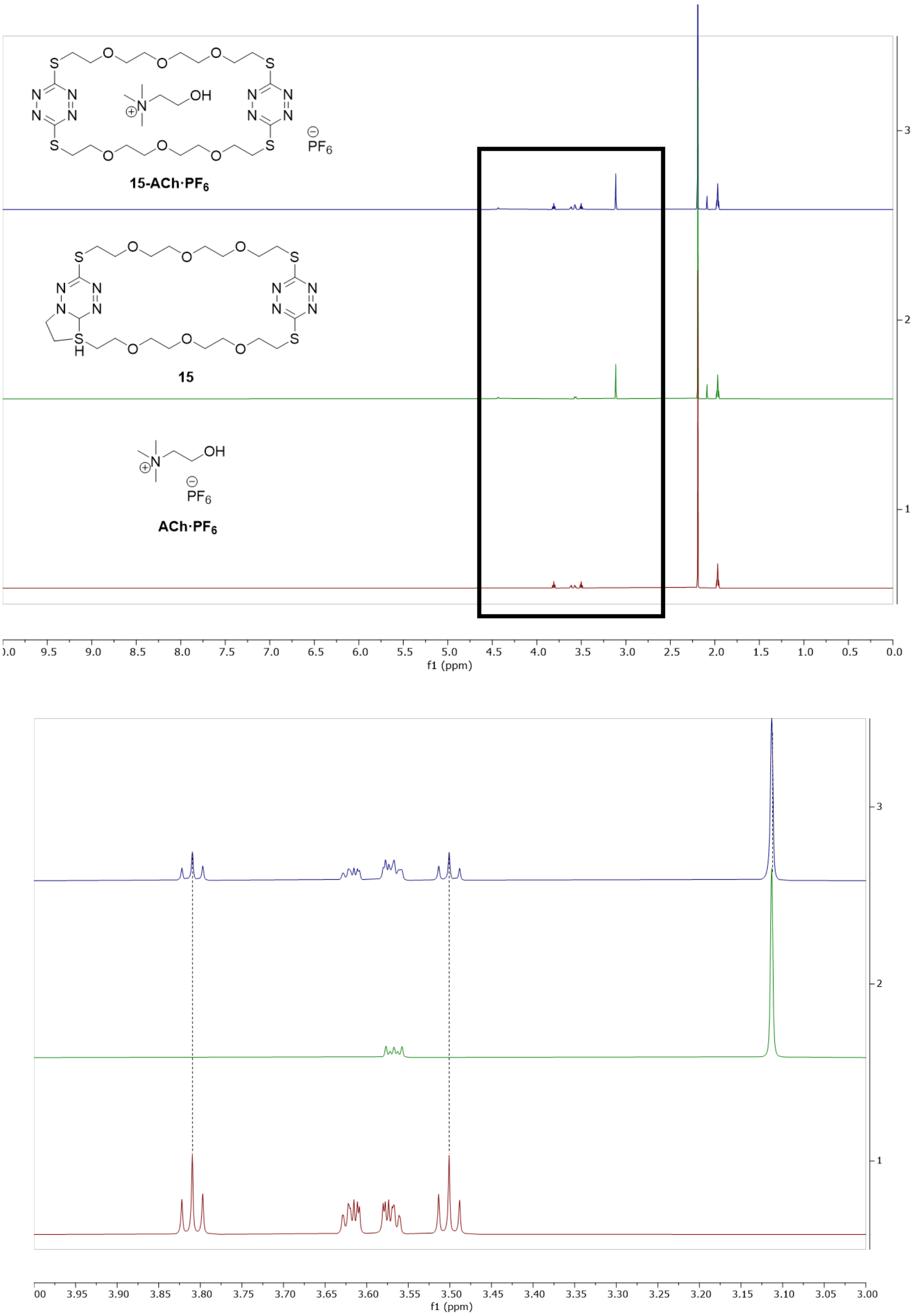

Figure S23.

Fluorescence spectrum of cage 17.

The fluorescence spectroscopic studies were performed on a Cary Eclipse Varian spectrofluorimeter (Mulgrave, Victoria, Australia), according to the following protocol: 1 mL of 1,52 · 10^{-5} M solution of capsule in acetone. The slit width was fixed to 2.5 nm for both excitation and emission experiments using a quartz cell for the measurements.

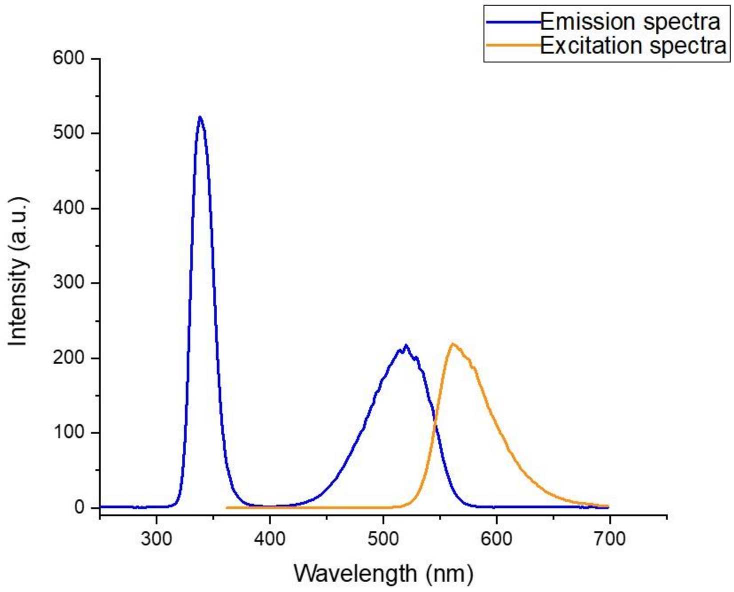

Figure S24.

Competitive redox reactions with thiols.

All the attempts of performing S_NTz with thiophenols and with some aliphatic thiols were unsuccessful due to a competitive reaction. Even when the synthesis of the corresponding tetrazine dimers were carried out with no problem, the exchange reaction did not progress in the expected way. Indeed, while the tetrazine reaction mixtures are characterized by an intense coloration (orange, pink or red, depending on the substrate), in the case of some thiols, a progressive decoloration was observed, which in DMSO was relatively quick (a few minutes). We hypothesized that decoloration was caused by a reduction of the

tetrazine by the thiol, yielding the corresponding dihydro-tetrazine (colorless or pale yellow) and the disulfide.

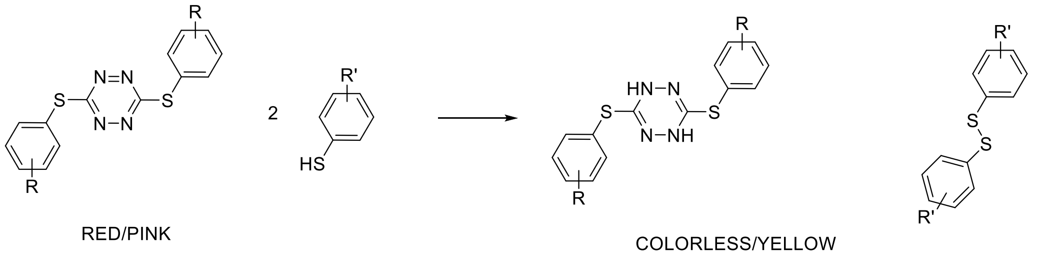

Figure S25.

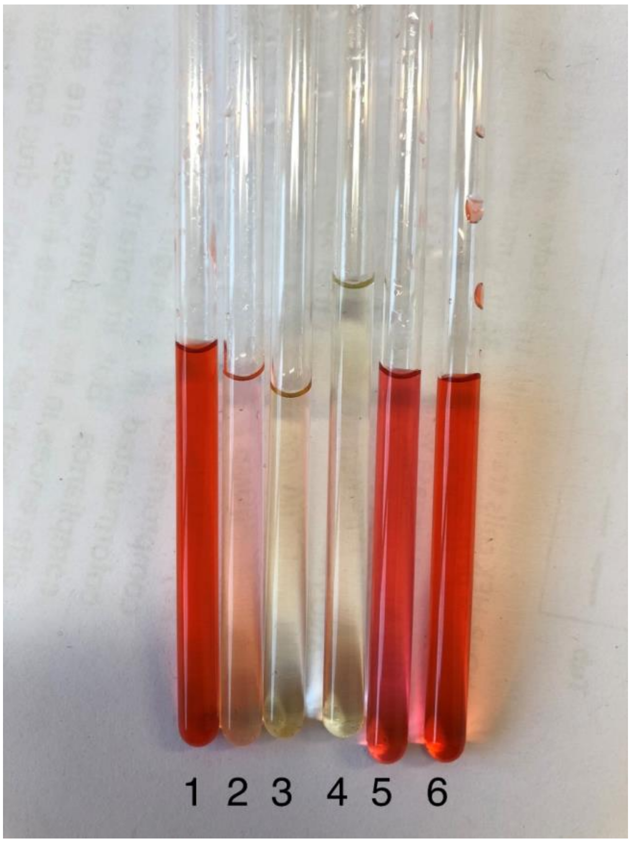

Figure S26. Comparison among NMR tubes containing phenol or alkyl thiol reactions (tubes 1, 5 and 6) with tubes containing thiophenols reactions (tubes 2, 3, 4)

In order to test that hypothesis, we performed an attempt of "exchange" reaction between p-methylthiophenol and its tetrazine dimer, with the purpose of simplify the NMR spectra and being able to follow the reaction in an easier way. As we anticipated, the peaks of the starting material progressively decreased and a new set of peaks grew up, corresponding

to the disulfide and the reduced tetrazine, which was also confirmed by ^{13}C NMR and by mass spectrometry.

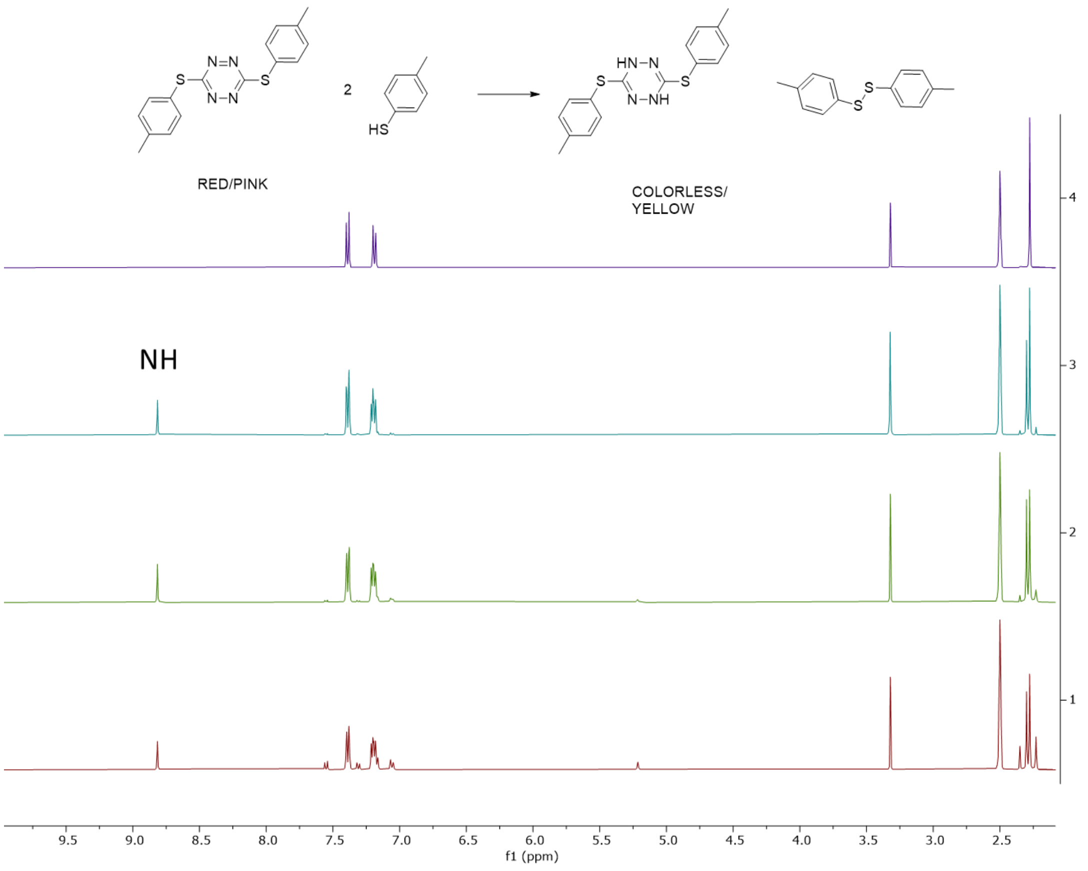

Figure S27.

Red NMR*: 5 minutes after addition of 4-methylthiophenol to a solution of the corresponding dimeric tetrazine in deuterated DMSO.*

Green NMR*: 10 minutes after addition*

Blue NMR*: 1h after addition*

Purple NMR*: ^{1}H NMR of commercial p-tolyl disulphide*

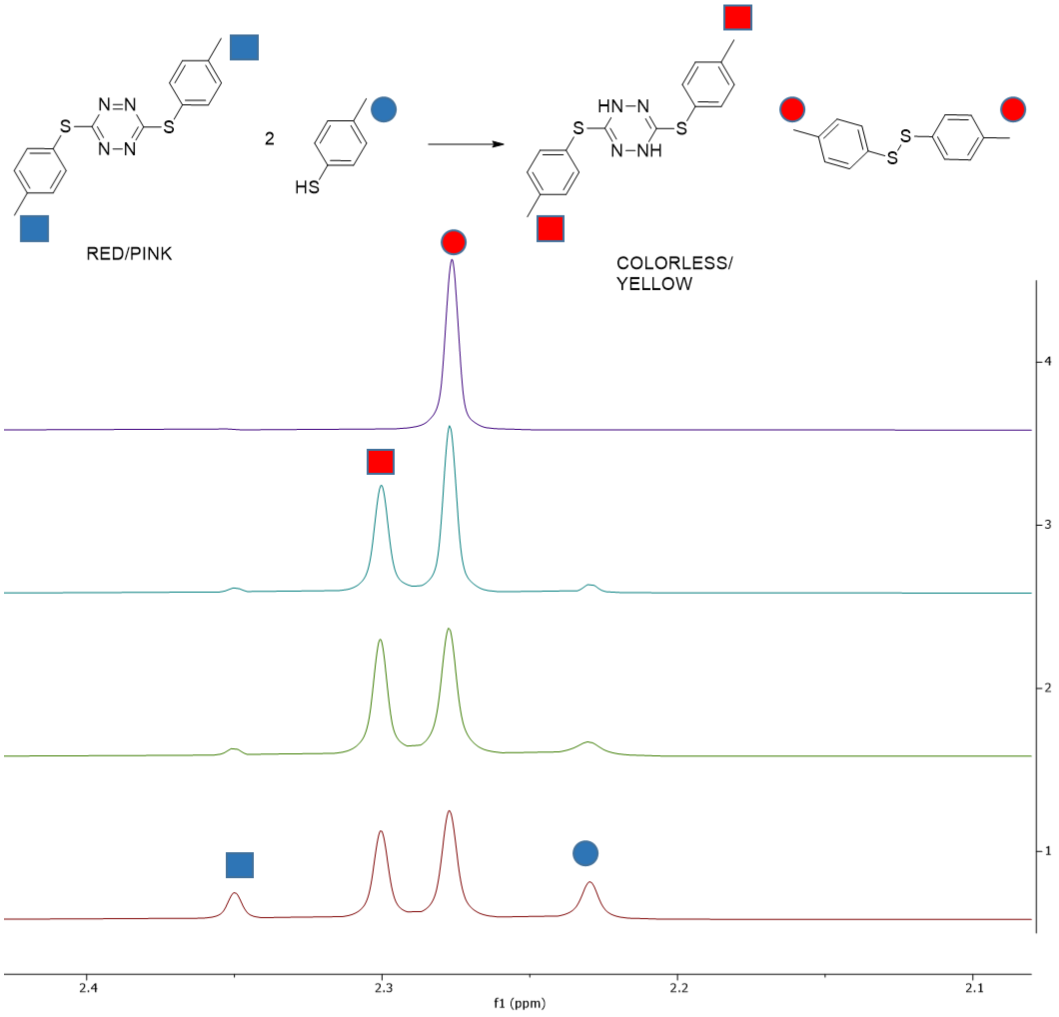

Figure S28.

***Red NMR**: 5 minutes after addition of 4-methylthiophenol to a solution of the corresponding dimeric tetrazine in deuterated DMSO.*

***Green NMR**: 10 minutes after addition*

***Blue NMR**: 1h after addition*

***Purple NMR**: ^{1}H NMR of commercial p-tolyl disulphide*

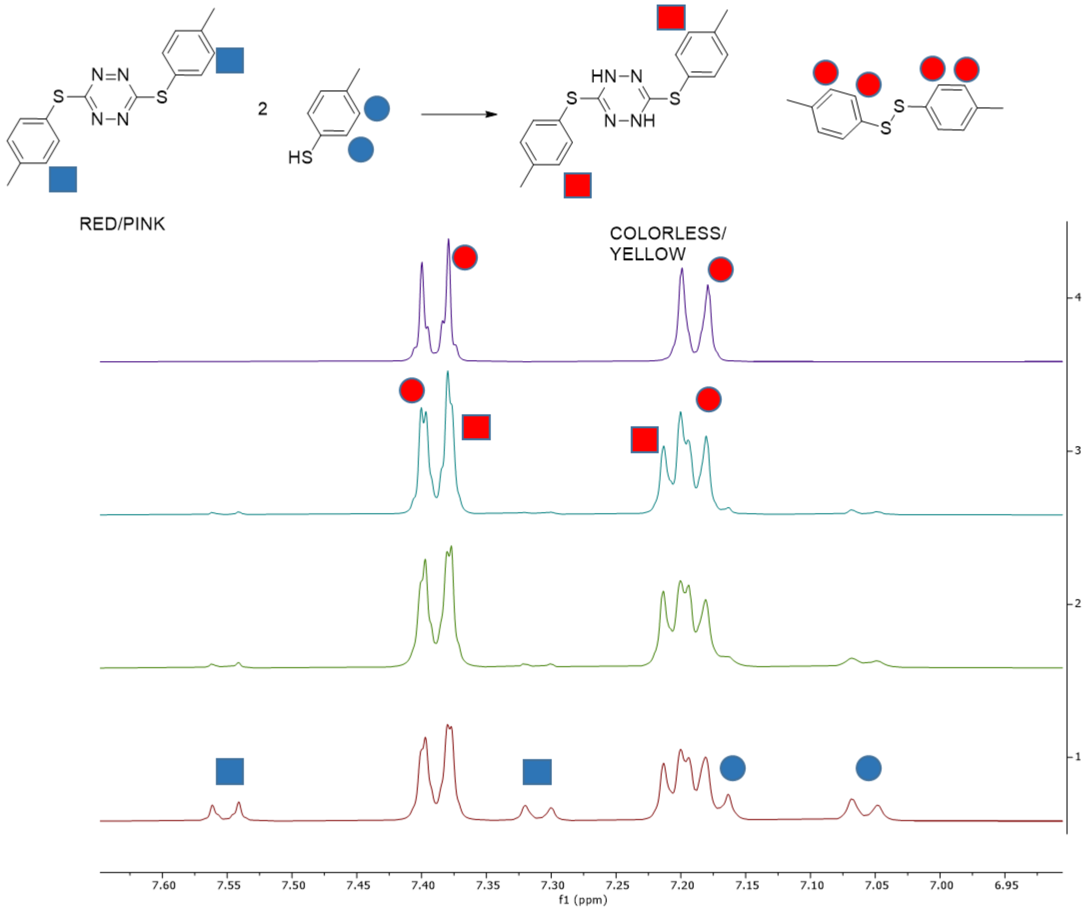

Figure S29.

***Red NMR**: 5 minutes after addition of 4-methylthiophenol to a solution of the corresponding dimeric tetrazine in deuterated DMSO.*

***Green NMR**: 10 minutes after addition*

***Blue NMR**: 1h after addition*

***Purple NMR**: ^{1}H NMR of commercial p-tolyl disulphide*

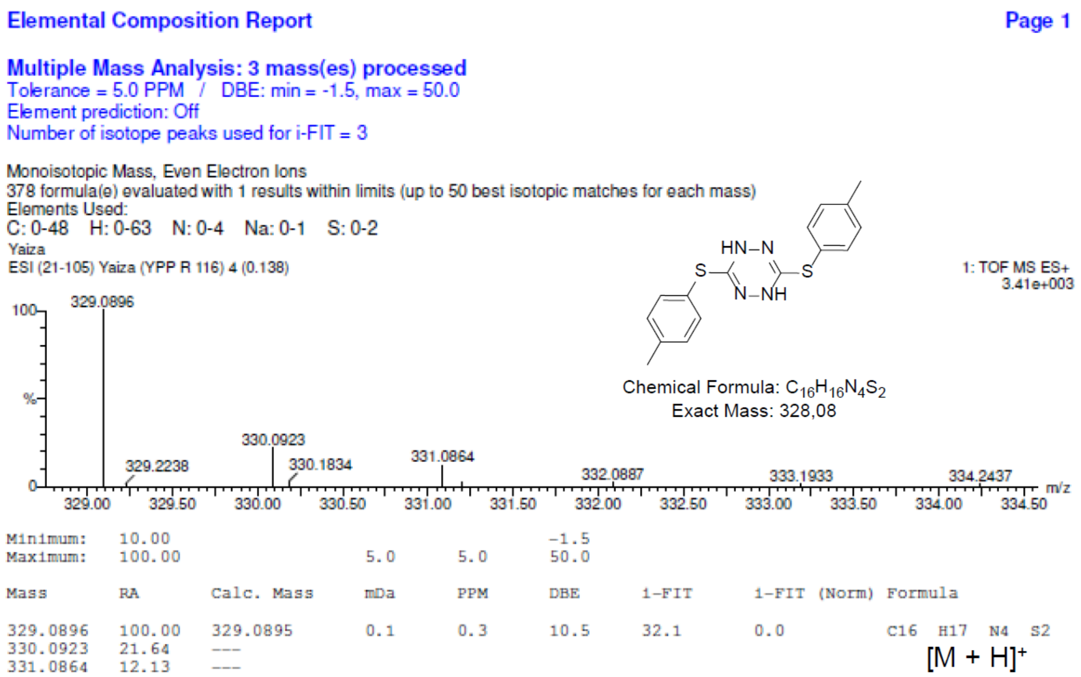

Elemental Composition Report **Page 1**

Multiple Mass Analysis: 3 mass(es) processed
Tolerance = 5.0 PPM / DBE: min = -1.5, max = 50.0
Element prediction: Off
Number of isotope peaks used for i-FIT = 3

Monoisotopic Mass, Even Electron Ions
378 formula(e) evaluated with 1 results within limits (up to 50 best isotopic matches for each mass)
Elements Used:
C: 0-48 H: 0-63 N: 0-4 Na: 0-1 S: 0-2
Yaiza
ESI (21-105) Yaiza (YPP R 116) 4 (0.138)

Minimum:	10.00				-1.5			
Maximum:	100.00		5.0	5.0	50.0			
Mass	RA	Calc. Mass	mDa	PPM	DBE	i-FIT	i-FIT (Norm)	Formula
329.0896	100.00	329.0895	0.1	0.3	10.5	32.1	0.0	C16 H17 N4 S2
330.0923	21.64	---						
331.0864	12.13	---						

[M + H]$^+$

Figure S30. Mass spectrometry (ESI) of the reaction mixture.

Additionally, although S,S-tetrazine derivatives with thiophenol can be synthesized, when reaction was submitted with 4 equivalents of thiophenol and 1 equivalent of **Cl-Tz-Cl**, the corresponding disulfide was cleanly obtained. Intriguingly, no peak of tetrazine is observed in the ^{13}C NMR of the reaction mixture. More experiments will be necessary to know the fate of **Cl-Tz-Cl**.

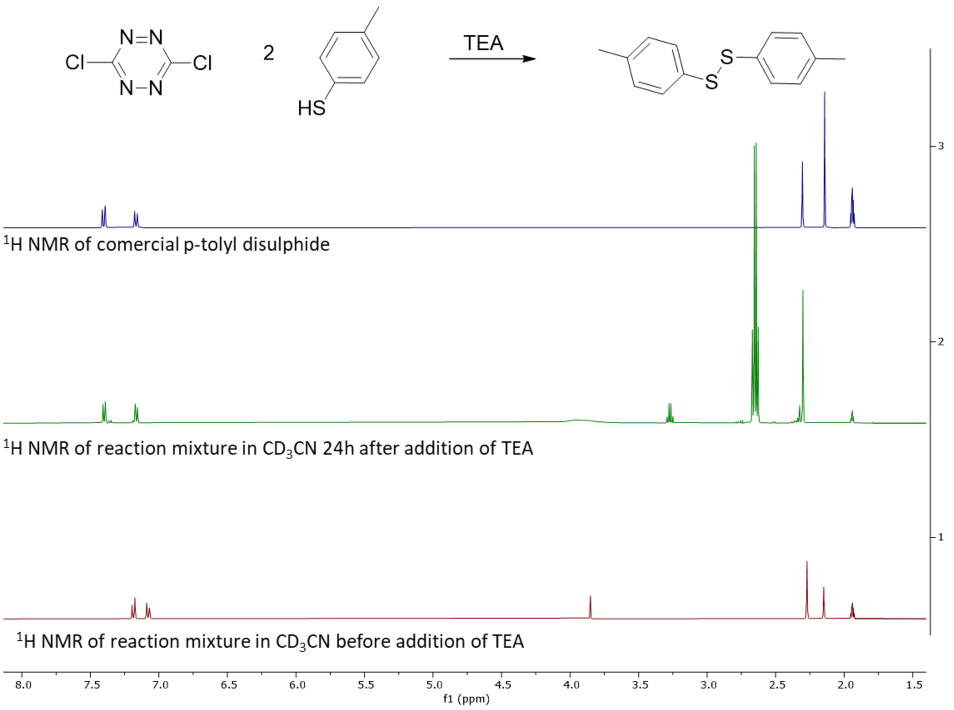

Figure S31.

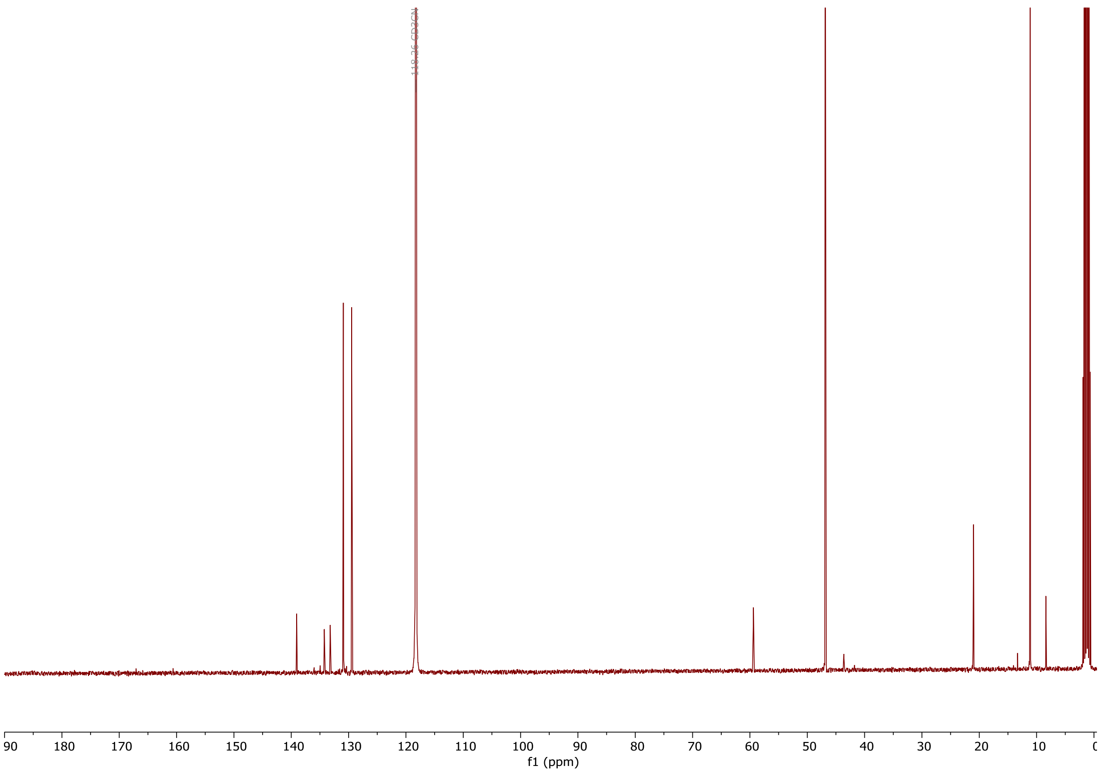

Figure S32. ^{13}C NMR of the reaction mixture in CD_3CN 24 h after addition of TEA

Dissasembly reaction of cage 17

To a solution of cage **17** (2.67 µmol, 2.6 mg) in deuterated acetonitrile (0.7 mL) was added dodecanethiol **7** (40.1 µmol, 8.9 µL) and triethylamine (40.1 µmol, 5.6 µL). After 30 minutes, the H^1 NMR showed no remaining cage and a new singlet grew up corresponding to phloroglucinol.

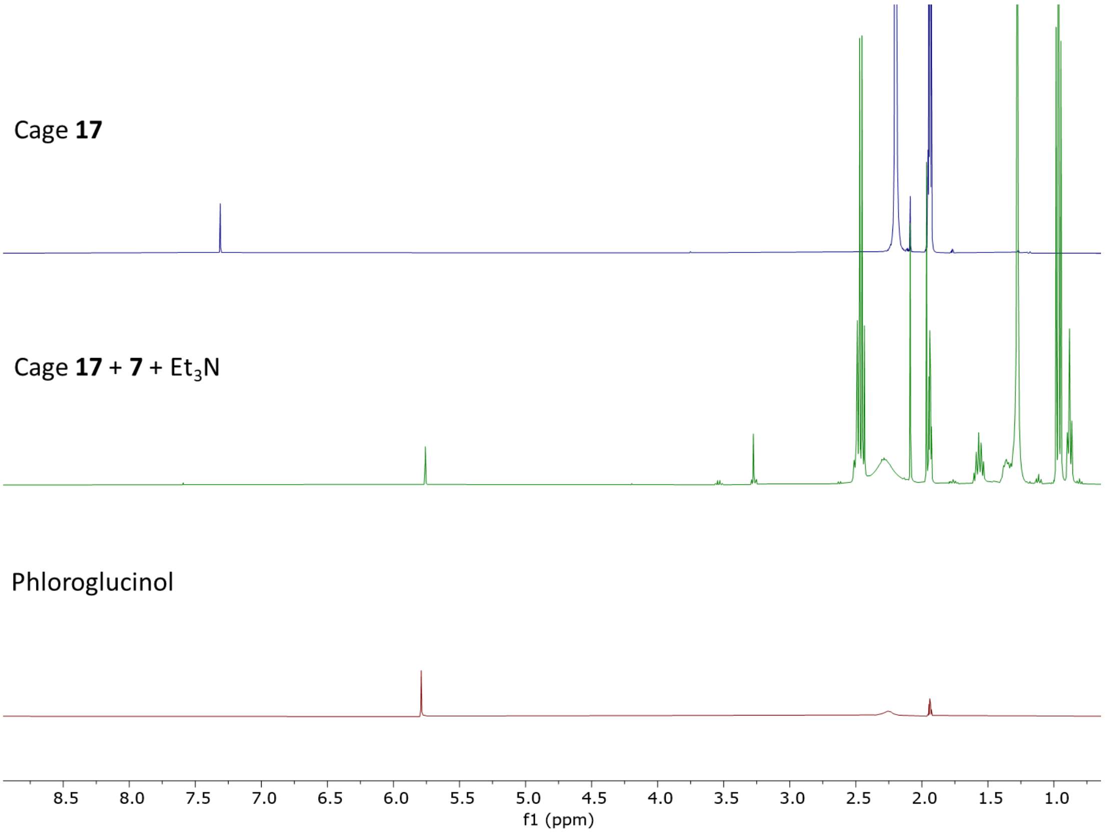

Figure S33.

Crystal Data and X-Ray Molecular Structure of 17

Table S2. Crystallographic and structure refinement details

	17
Formula	$C_{42}H_{24}N_{24}O_{14}$
M (g mol^{-1})	1088.85
Crystal system	Orthorhombic
Space group	$Pna2_1$
a (Å)	27.0686(15)
b (Å)	11.0085(5)
c (Å)	16.2348(8)
V (Å^3)	4837.8(4)
Z	4
ρ_{calc} (g cm^{-3})	1.495
μ (mm^{-1})	0.118
T (K)	293
λ (Å)	0.71073
Reflect. collcd.	40261
Reflect. obs. [$I > 2\sigma(I)$]	11057 (5696)
Data Restraints/ Parameters	1/726
R_1^a [$I > 2\sigma(I)$]	0.1680 (0.0804)
wR_2^b [$I > 2\sigma(I)$]	0.1687 (0.1395)
S^c	1.102

^{a} $R_1 = \sum(|F_o| - |F_c|)/\sum|F_o|$.
^{b} $wR_2 = [\sum w(F_o^2 - F_c^2)^2/\sum w(F_o^2)^2]^{1/2}$.
^{c} $S = [\sum w(|F_o| - |F_c|)^2/(N_o - N_p)]^{1/2}$.

Data collection for single crystals of cage **17** was performed on an Agilent Supernova X-ray μ-focus diffractometer at 293 K using Mo radiation (λ = 0.71073 Å). Data were indexed, integrated and scaled with the Rigaku CrysAlisPro software.[8] The crystal structure was solved by intrinsic phasing with SHELXT software[9] and refined with the full-matrix least squares techniques of F^2 by using SHELXL program[10] within the Olex2 software.[11] All non-hydrogen atoms were refined anisotropically. The hydrogen atoms of the organic ligands and the two acetone solvent molecules were set on geometrical positions and refined with a riding model. Some nitrogen atoms of the tetrazine molecules have anisotropic displacement factors larger than usual, reflecting the free rotation capacity of these groups. Modelling this disorder will increase the number of parameters, without a big improvement in the R factors.

The final dataset has a poor $I/I > 2\sigma(I)$ ratio due to the poor diffraction power of the sample at high angles. This is reflected not only in the R and wR_2 values when the total number of reflections are taken into account but also in the lack of reliability of the Flack factor although the number of Friedel pairs collected are almost complete.

The cif-file was deposited in the Cambridge Structural Database under identifier CCDC 2067785.

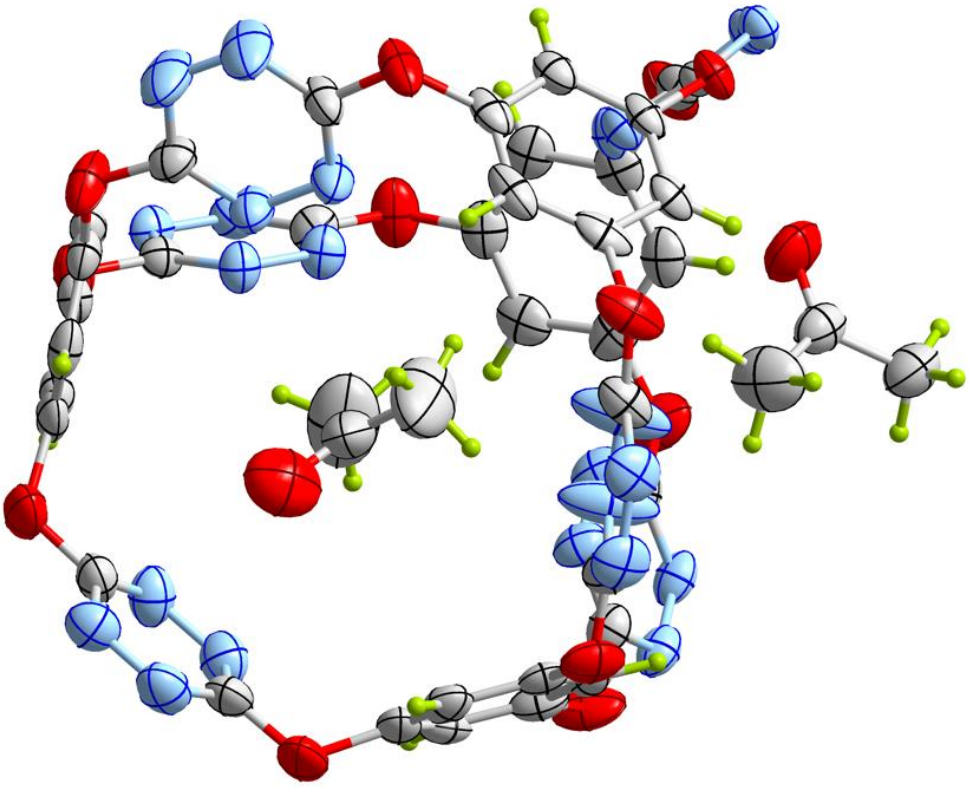

Figure S34.

Each tetrahedral cage is formed by four phloroglucinol groups located in the vertices and six rod-like tetrazine groups on the edges. They form an almost perfect tetrahedron able to accommodate a sphere of 7 Å of diameter inside.

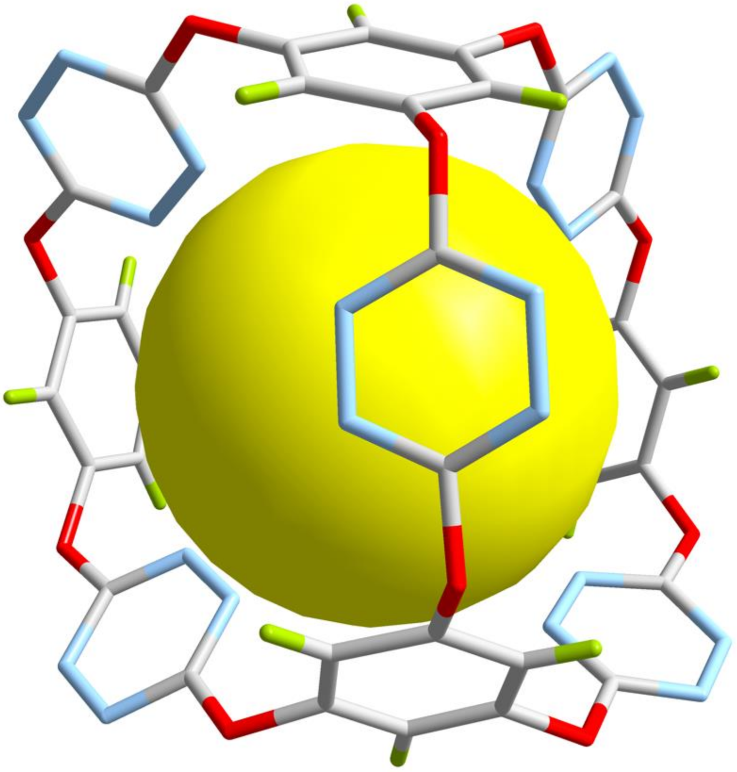

Figure S35.

The trigonal symmetry is broken by one of the acetone molecules located inside the cage, anchored by weak C=O/π interactions with a tetrazine ring. The tetrazine rings of the edges are coplanar with the plane formed by the corresponding edge and the center of the tetrahedron, except for that participating in the C=O/π interaction with the acetone molecule inside the cage. The high anisotropic displacement parameters for the nitrogen atoms of the tetrazine groups indicate that most likely they can rotate freely on the O···O

axis. The second acetone molecule is located in the voids between cages, but also anchored by C=O/π interactions with a tetrazine.

The cages are closely packed in the crystallographic *bc* plane forming corrugated layers with a square lattice of cages located in the same plane and the cage occupying the center of the squares is slightly displaced from lattice. These layers of cages are stacked along the *a* axis, separated enough to accommodate the acetone solvent molecule in the voids.

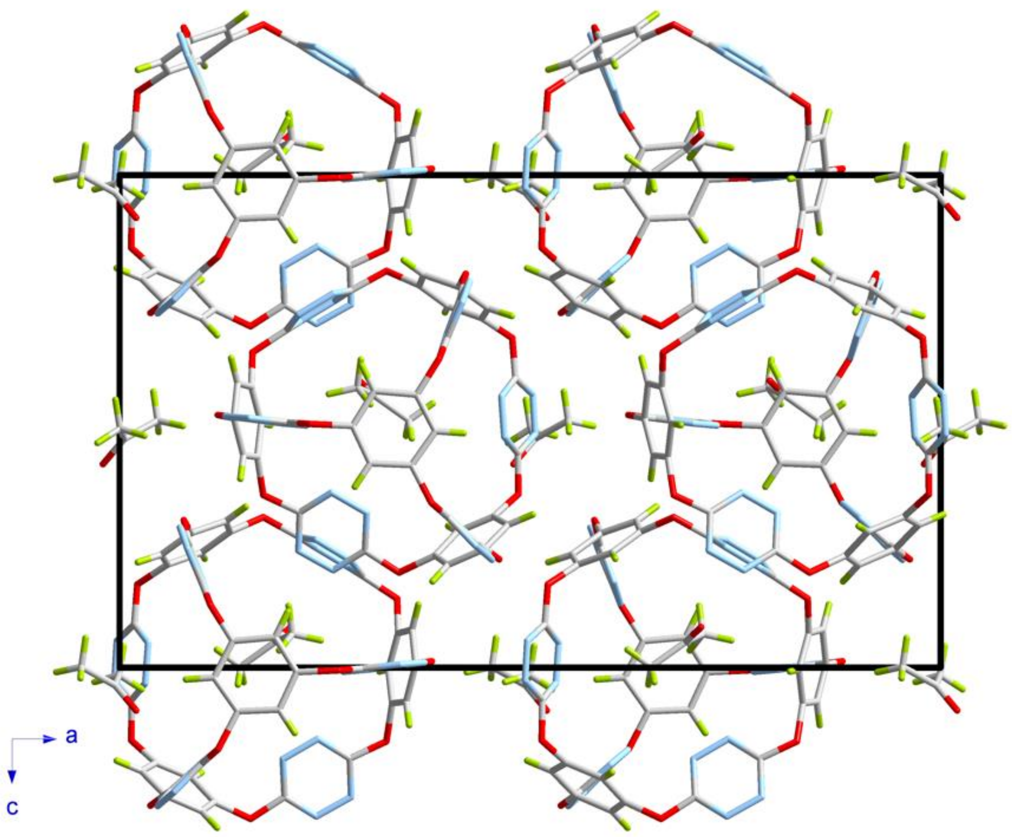

Figure S36.

NMR spectra

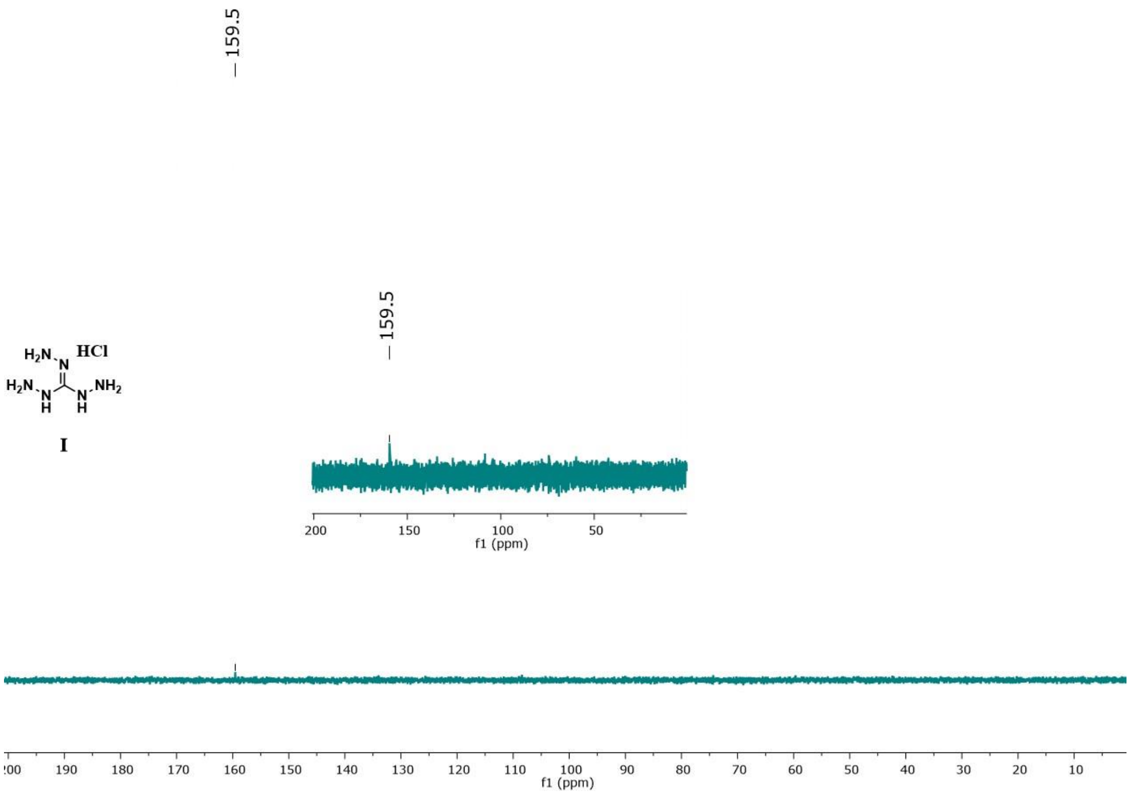

Figure S37. *^{13}C NMR spectrum (126 MHz, D_2O, 298 K) of **I**.*

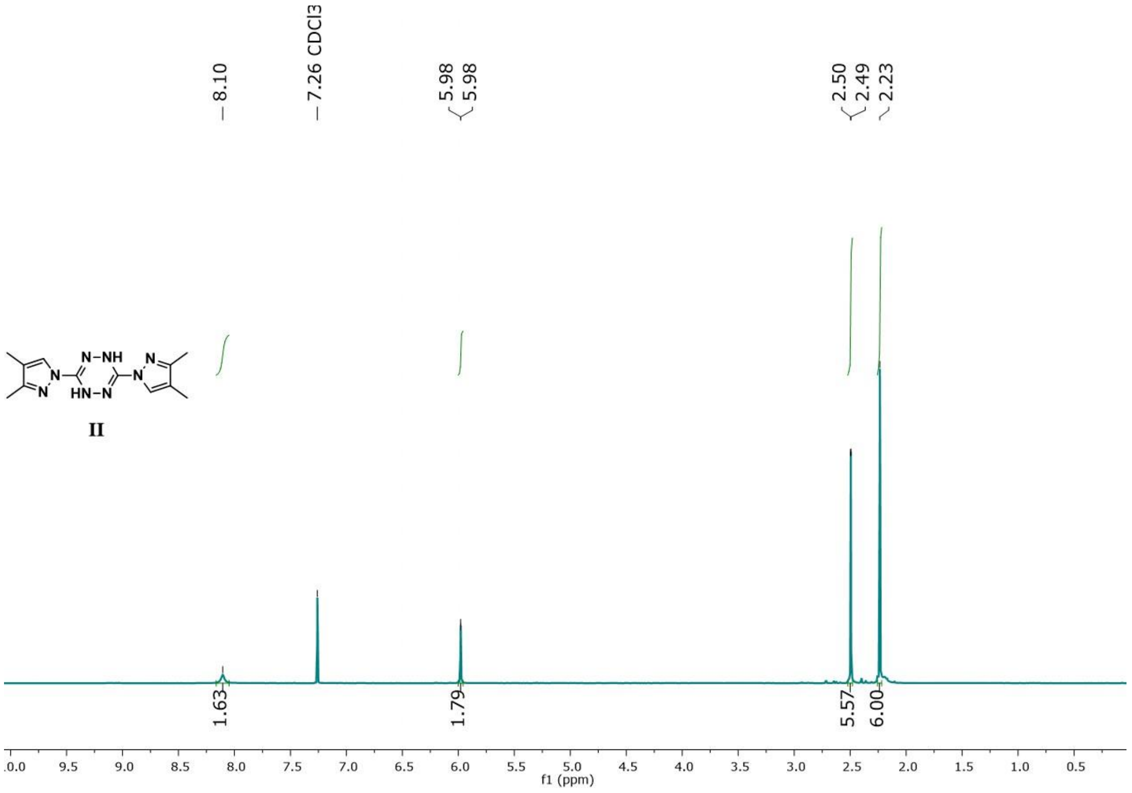

Figure S38. *^{1}H NMR spectrum (500 MHz, $CDCl_3$, 298 K) of **II**.*

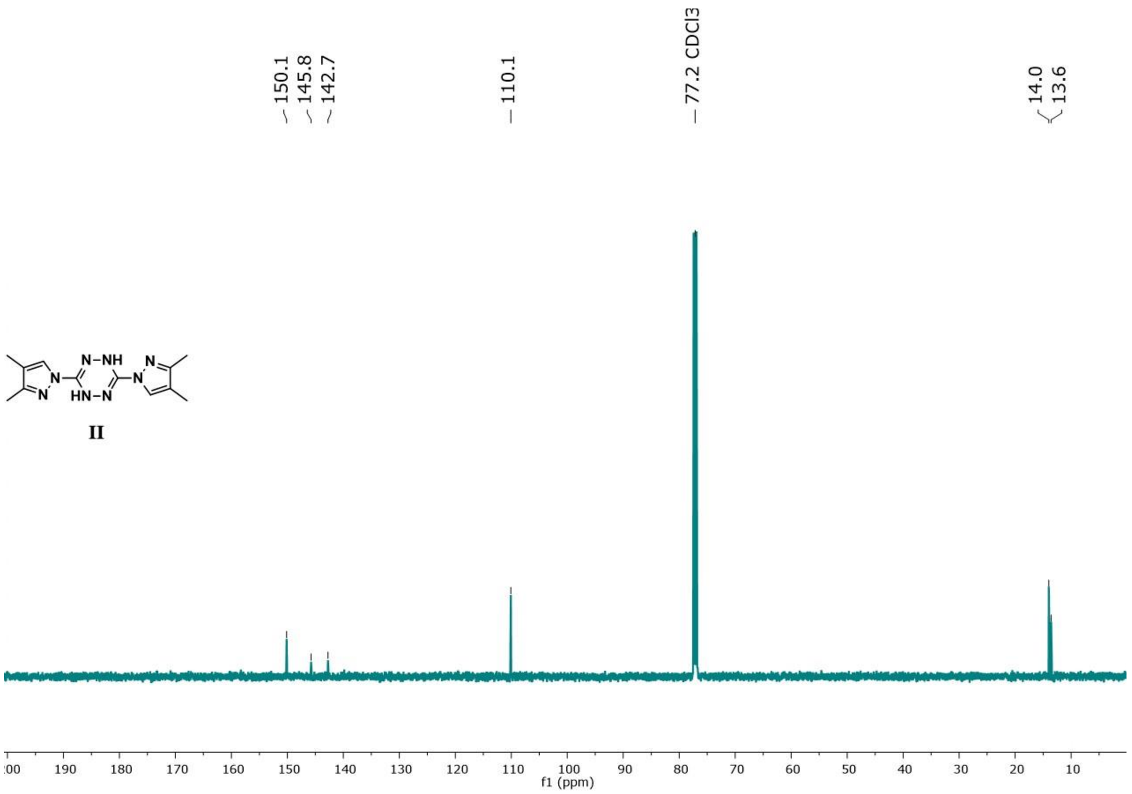

Figure S39. *^{13}C NMR spectrum (126 MHz, $CDCl_3$, 298 K) of* ***II***.

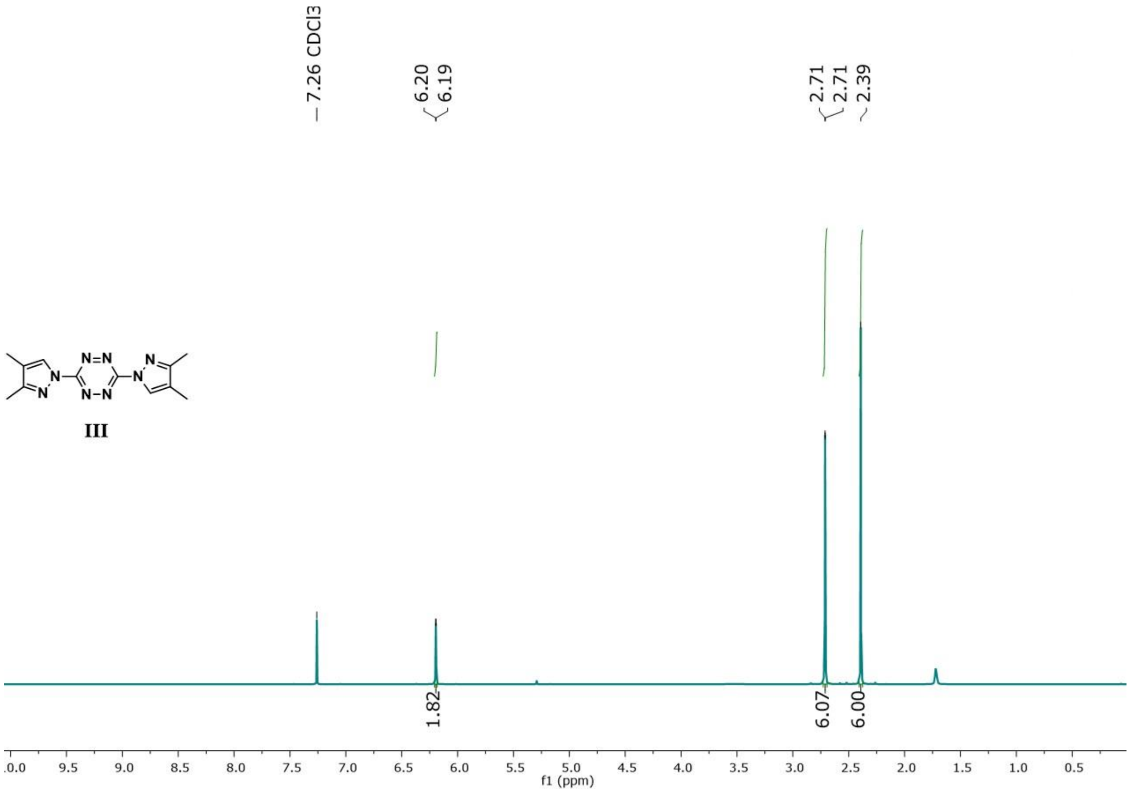

Figure S40. *^{1}H NMR spectrum (500 MHz, $CDCl_3$, 298 K) of* ***III***.

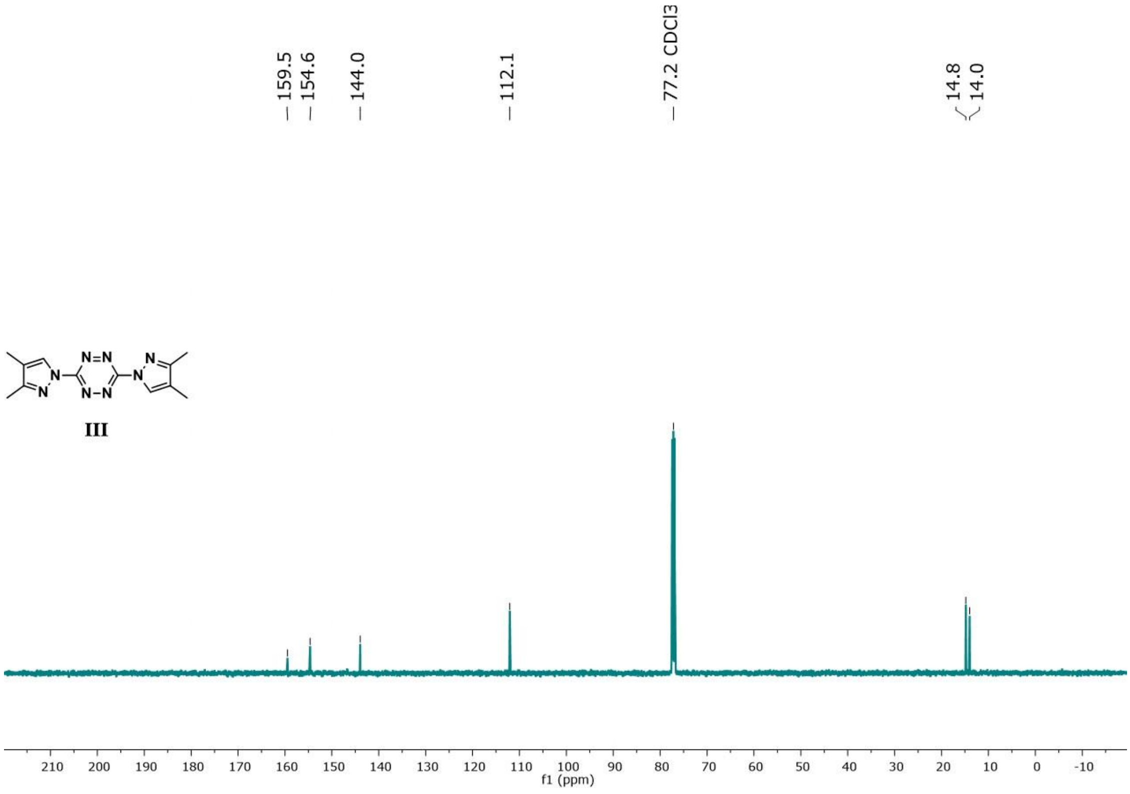

Figure S41. *^{13}C NMR spectrum (126 MHz, CDCl$_3$, 298 K) of* ***III.***

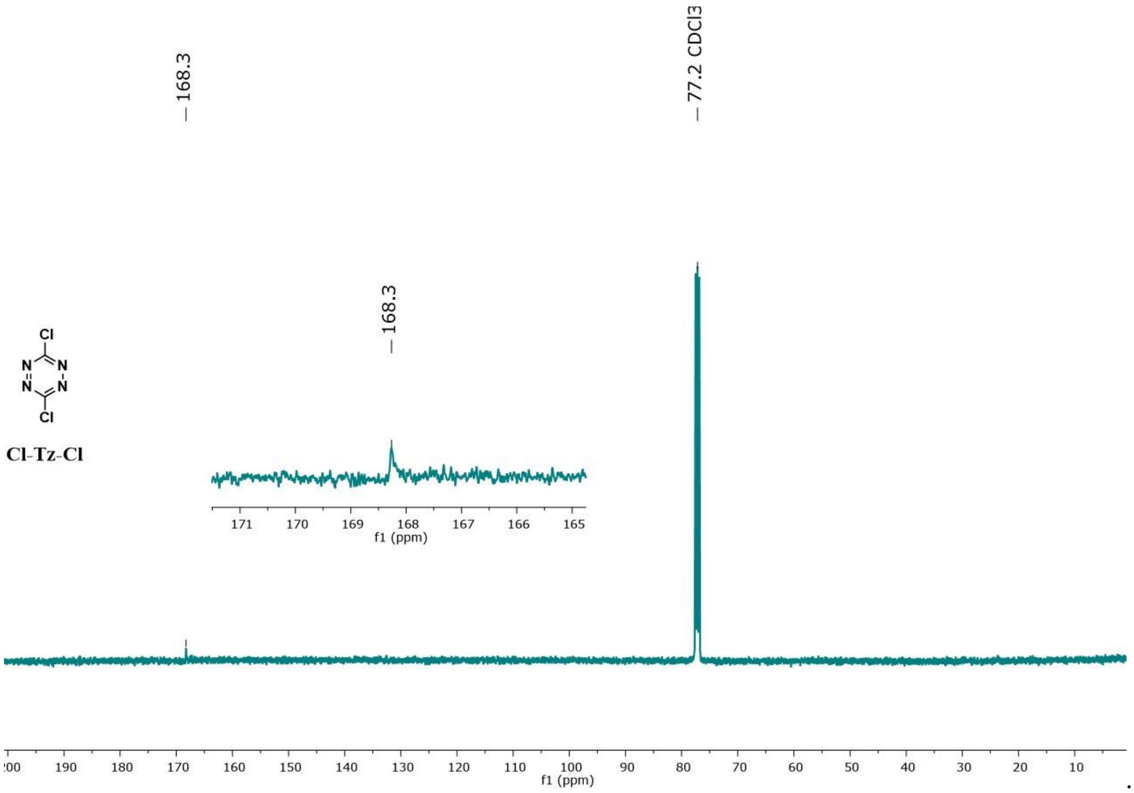

Figure S42. *^{13}C NMR spectrum (126 MHz, CDCl$_3$, 298 K) of* ***Cl-Tz-Cl.***

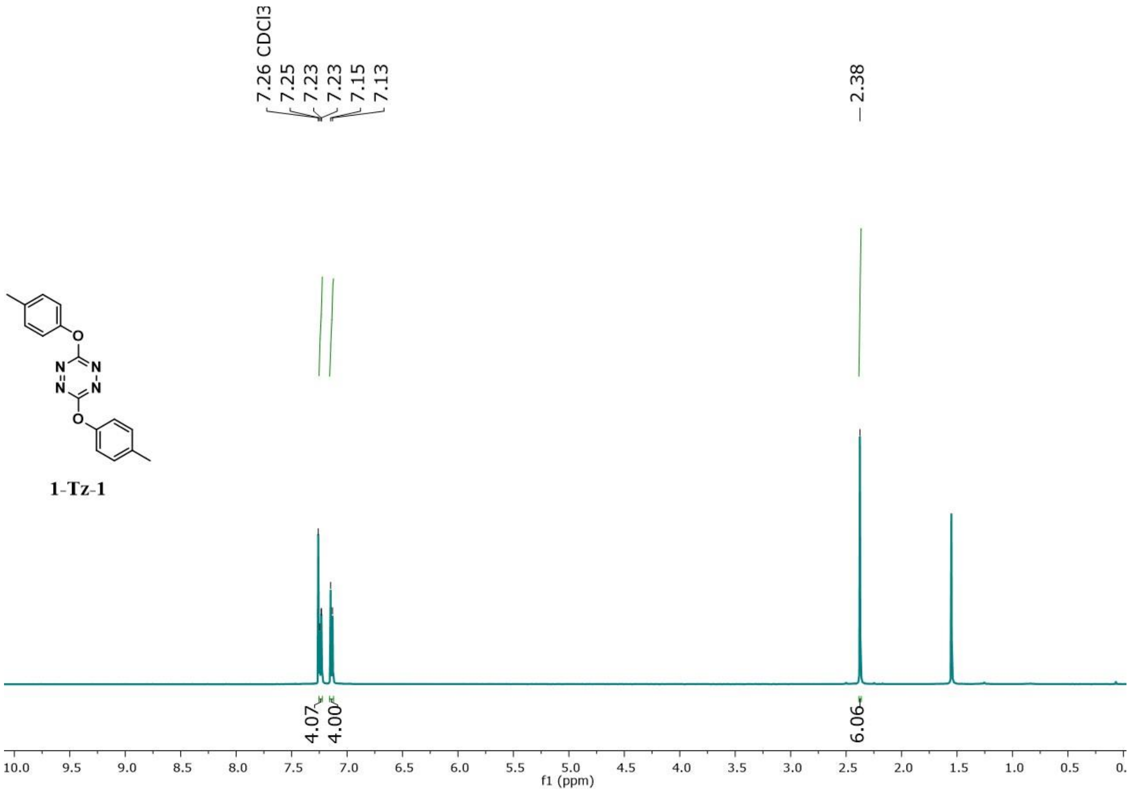

Figure S43. *^{1}H NMR spectrum (500 MHz, CDCl$_3$, 298 K) of* ***1-Tz-1****.*

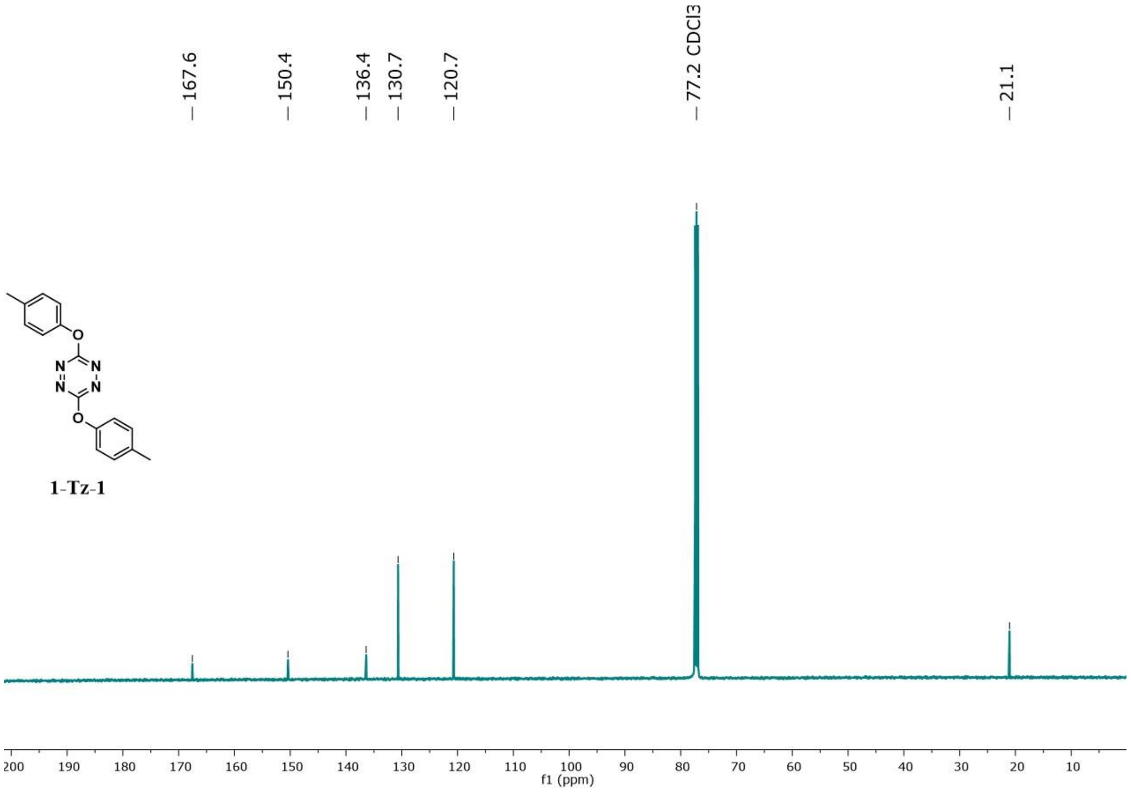

Figure S44. *^{13}C NMR spectrum (126 MHz, CDCl$_3$, 298 K) of* ***1-Tz-1****.*

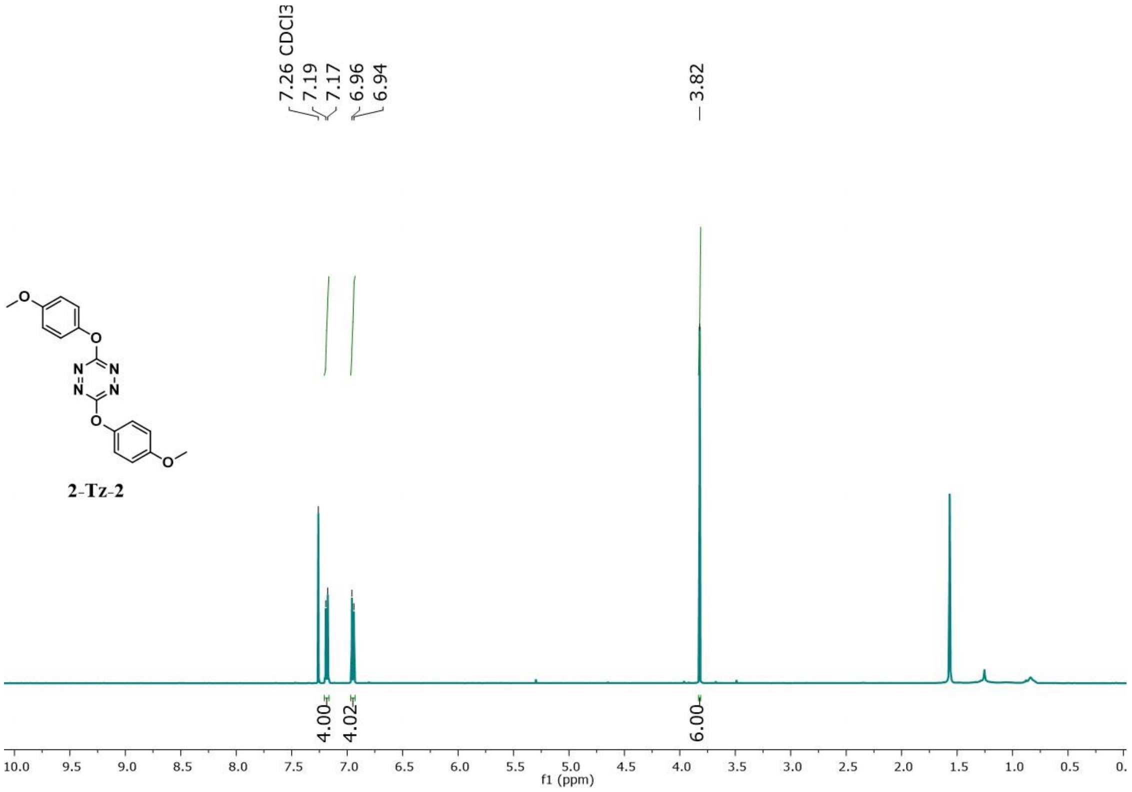

Figure S45. *^{1}H NMR spectrum (500 MHz, $CDCl_3$, 298 K) of* ***2-Tz-2****.*

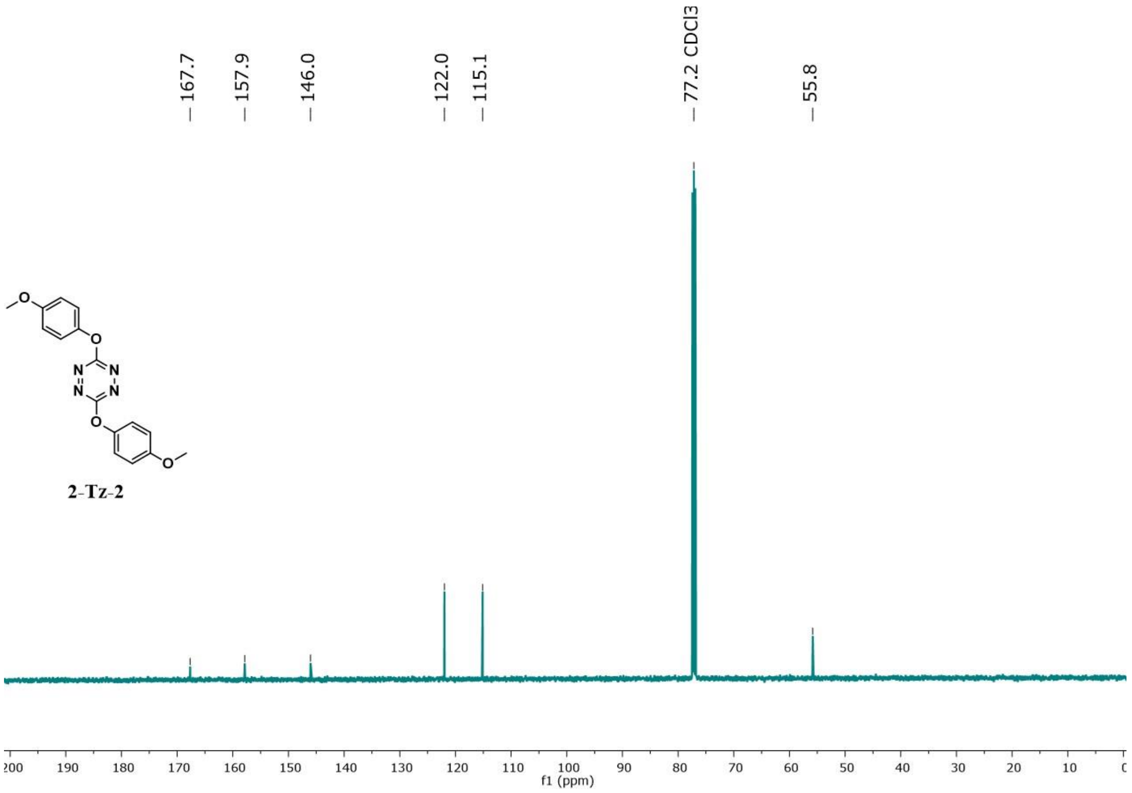

Figure S46. *^{13}C NMR spectrum (126 MHz, $CDCl_3$, 298 K) of* ***2-Tz-2****.*

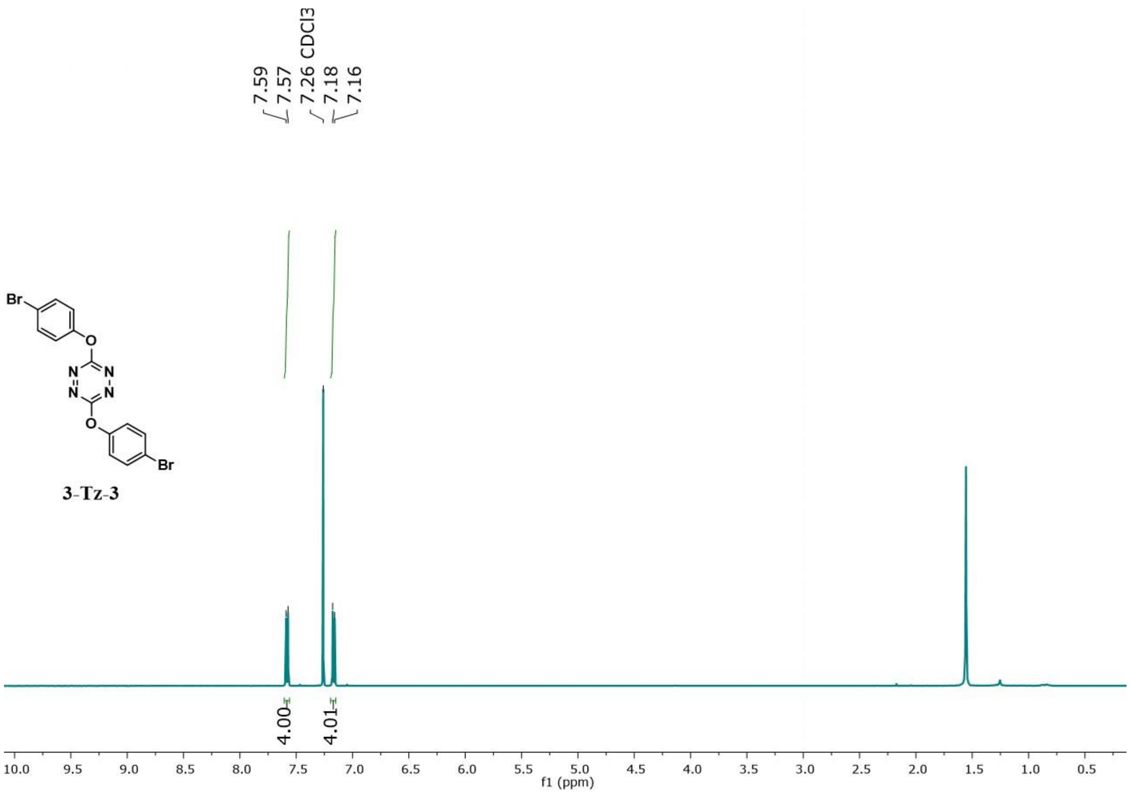

Figure S47. *^{1}H NMR spectrum (500 MHz, $CDCl_3$, 298 K) of* ***3-Tz-3****.*

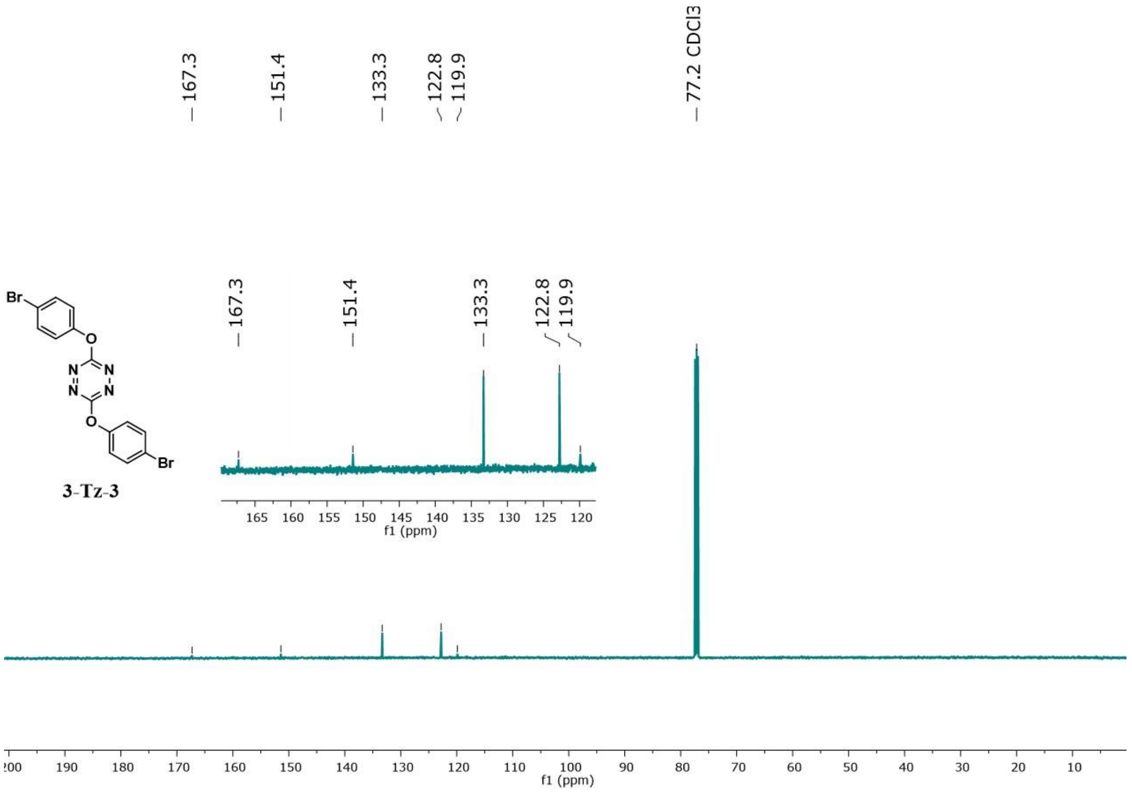

Figure S48. *^{13}C NMR spectrum (126 MHz, $CDCl_3$, 298 K) of* ***3-Tz-3****.*

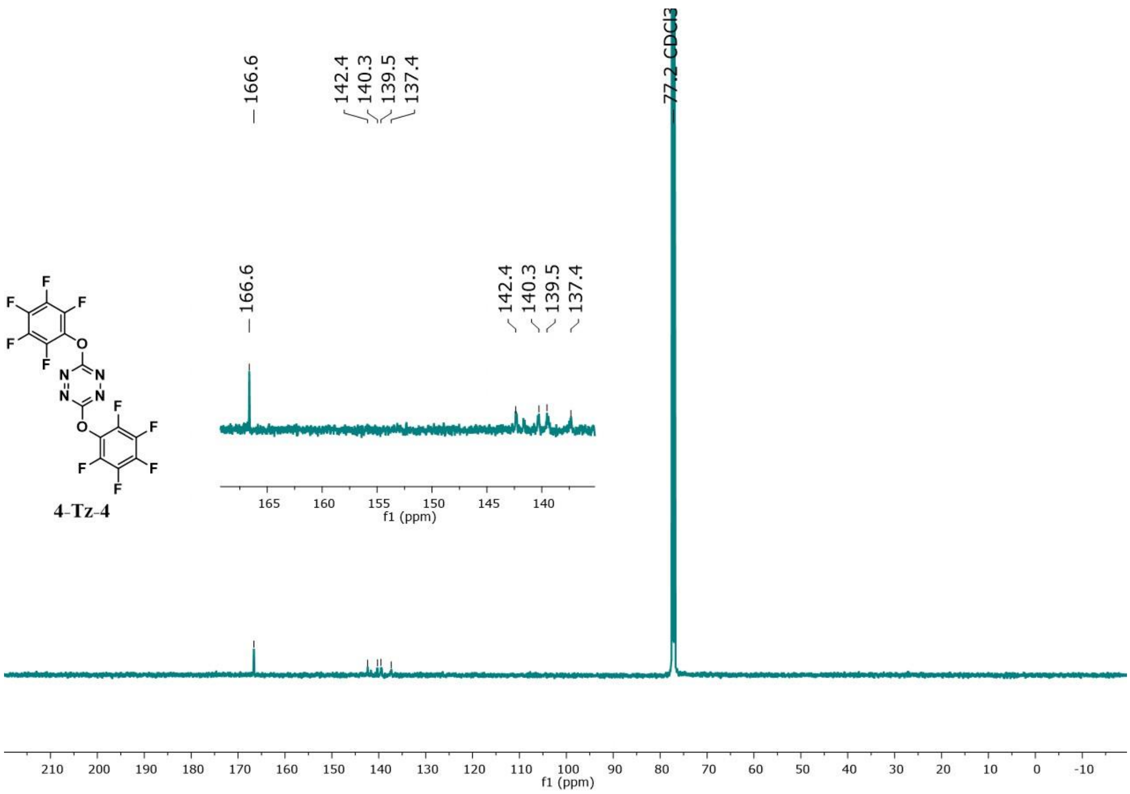

Figure S49. *^{13}C NMR spectrum (126 MHz, $CDCl_3$, 298 K) of* ***4-Tz-4****.*

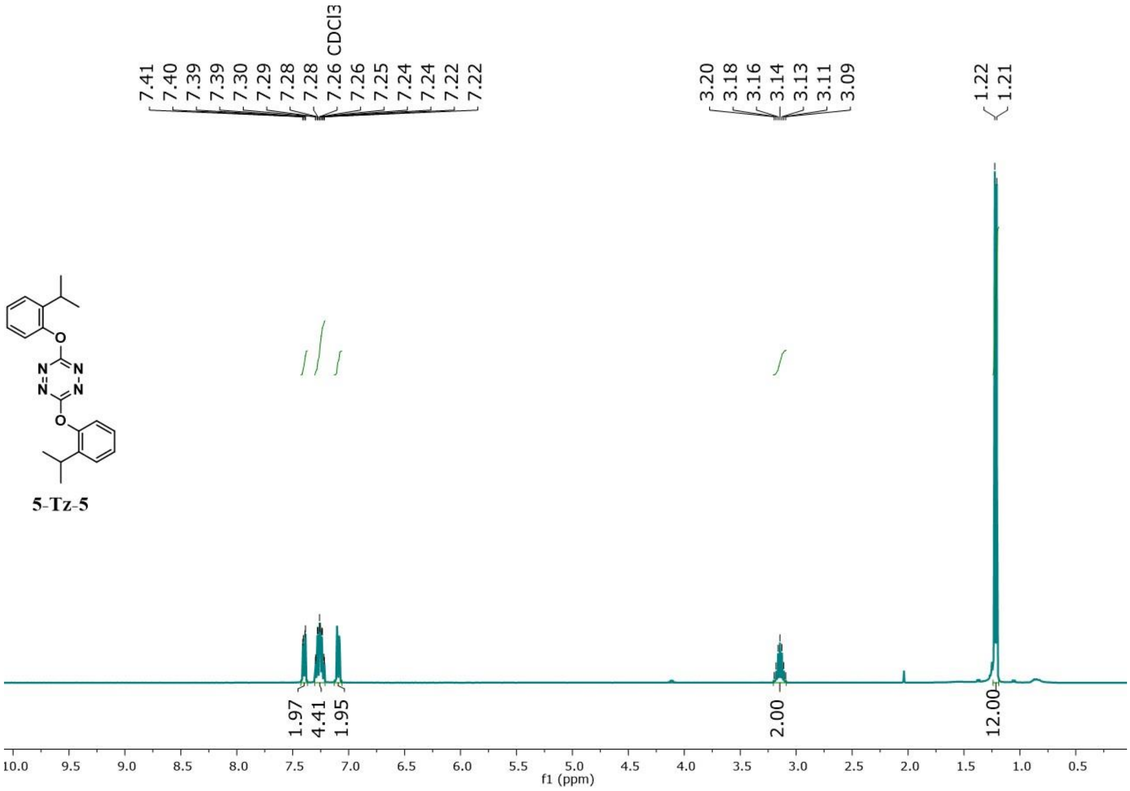

Figure S50. *^{1}H NMR spectrum (400 MHz, $CDCl_3$, 298 K) of* ***5-Tz-5****.*

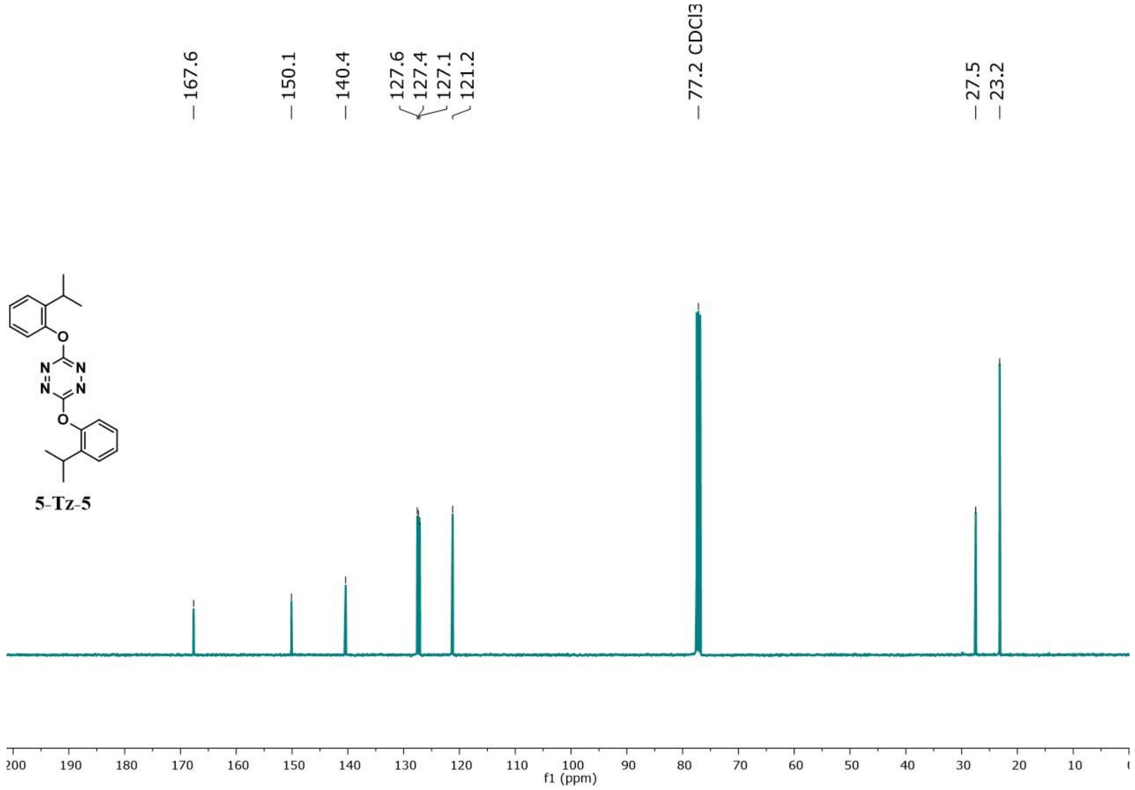

Figure S51. *^{13}C NMR spectrum (101 MHz, $CDCl_3$, 298 K) of* ***5-Tz-5****.*

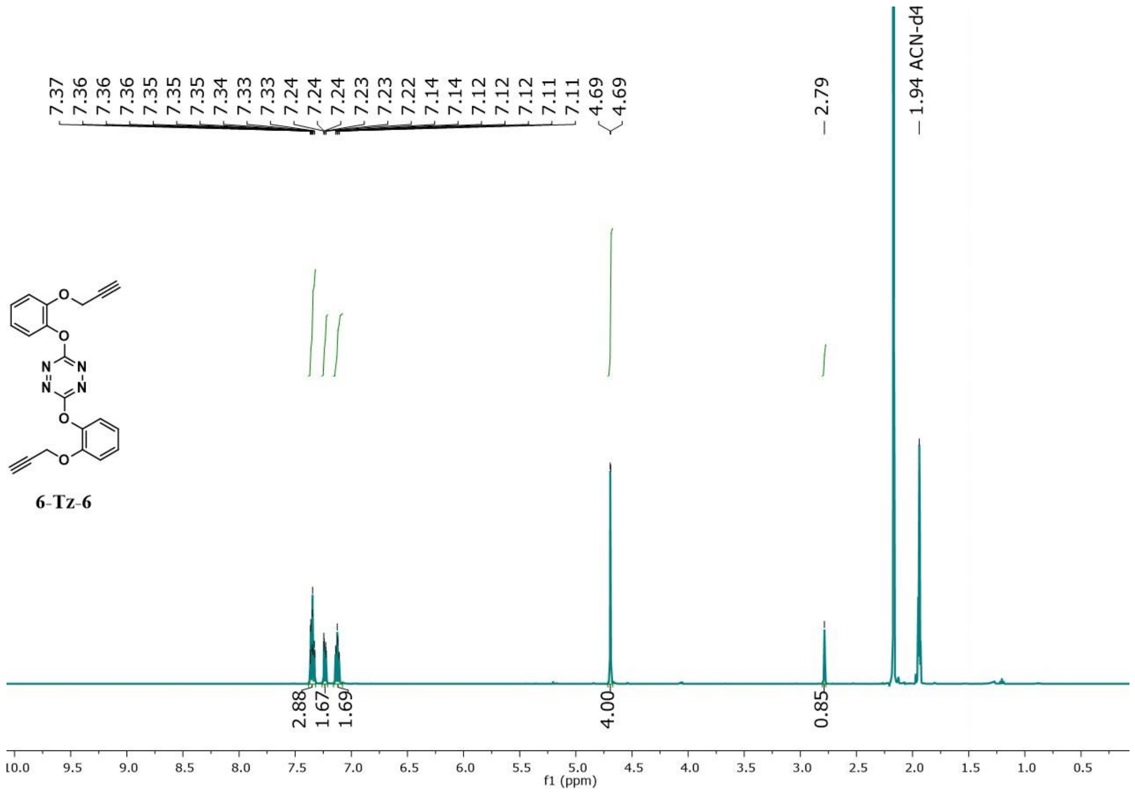

Figure S52. *^{1}H NMR spectrum (500 MHz, CD_3CN, 298 K) of* ***6-Tz-6****.*

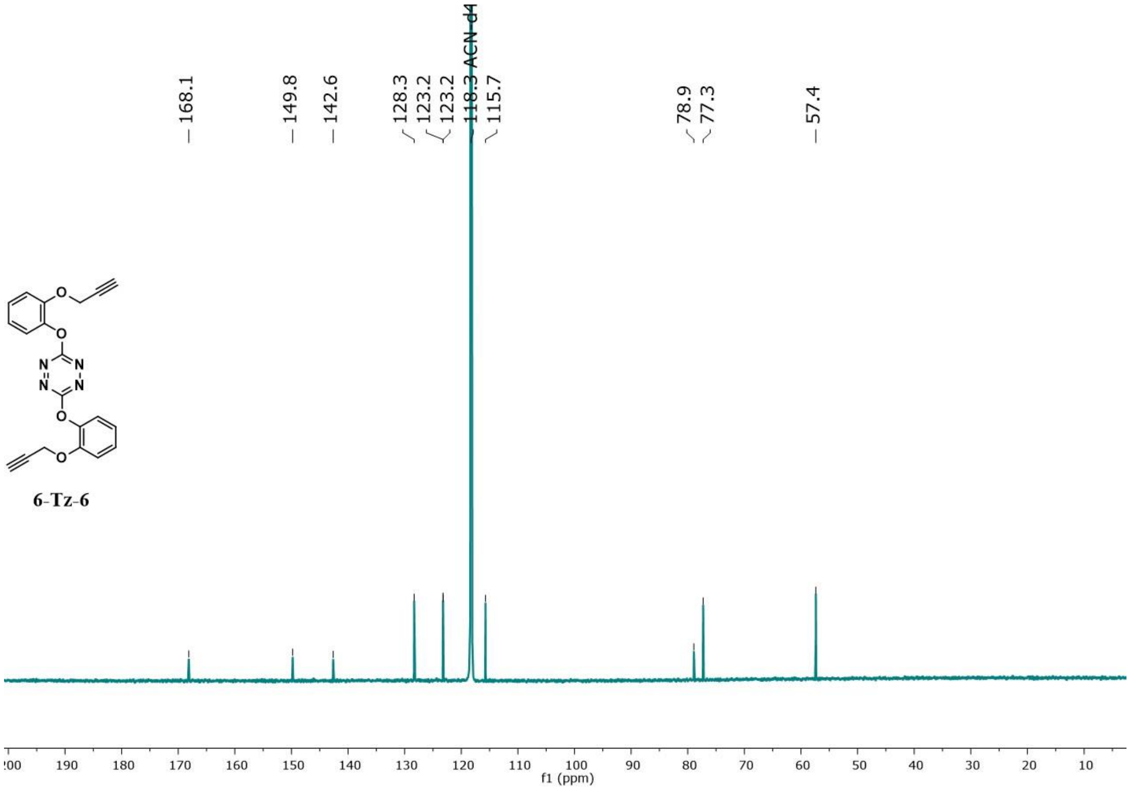

Figure S53. *^{13}C NMR spectrum (126 MHz, CD_3CN, 298 K) of* ***6-Tz-6****.*

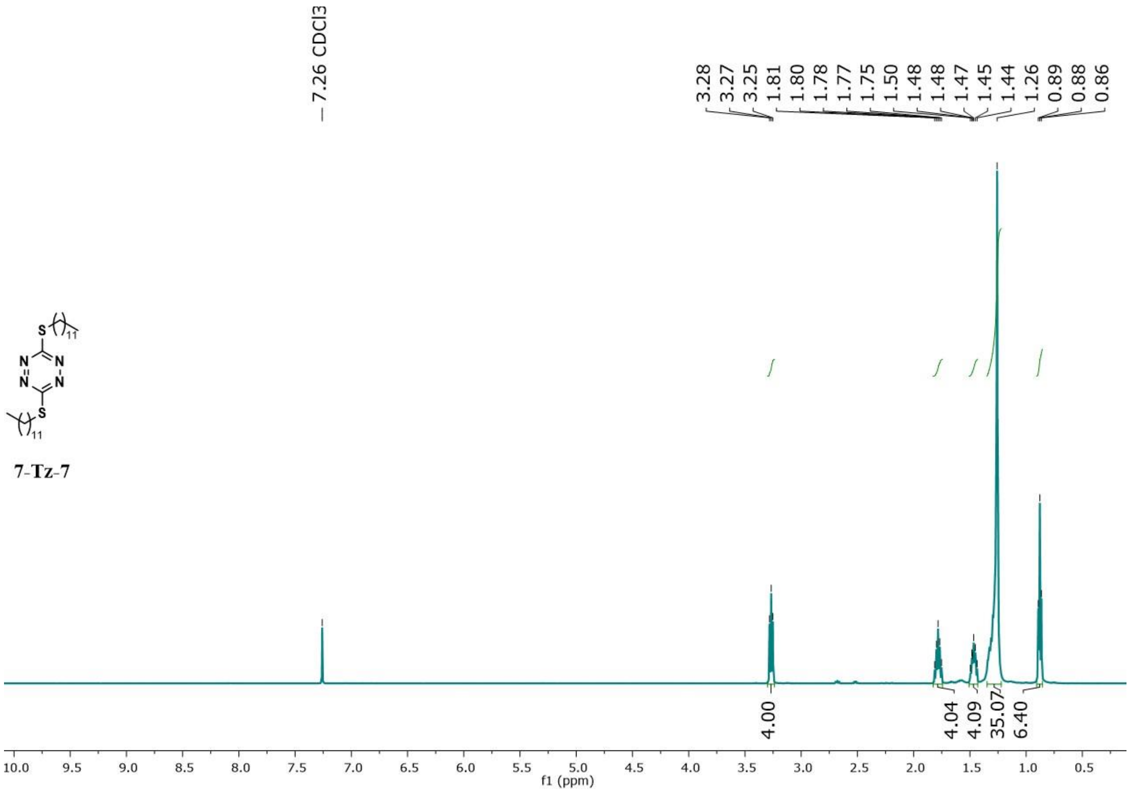

Figure S54. *^{1}H NMR spectrum (500 MHz, $CDCl_3$, 298 K) of* ***7-Tz-7****.*

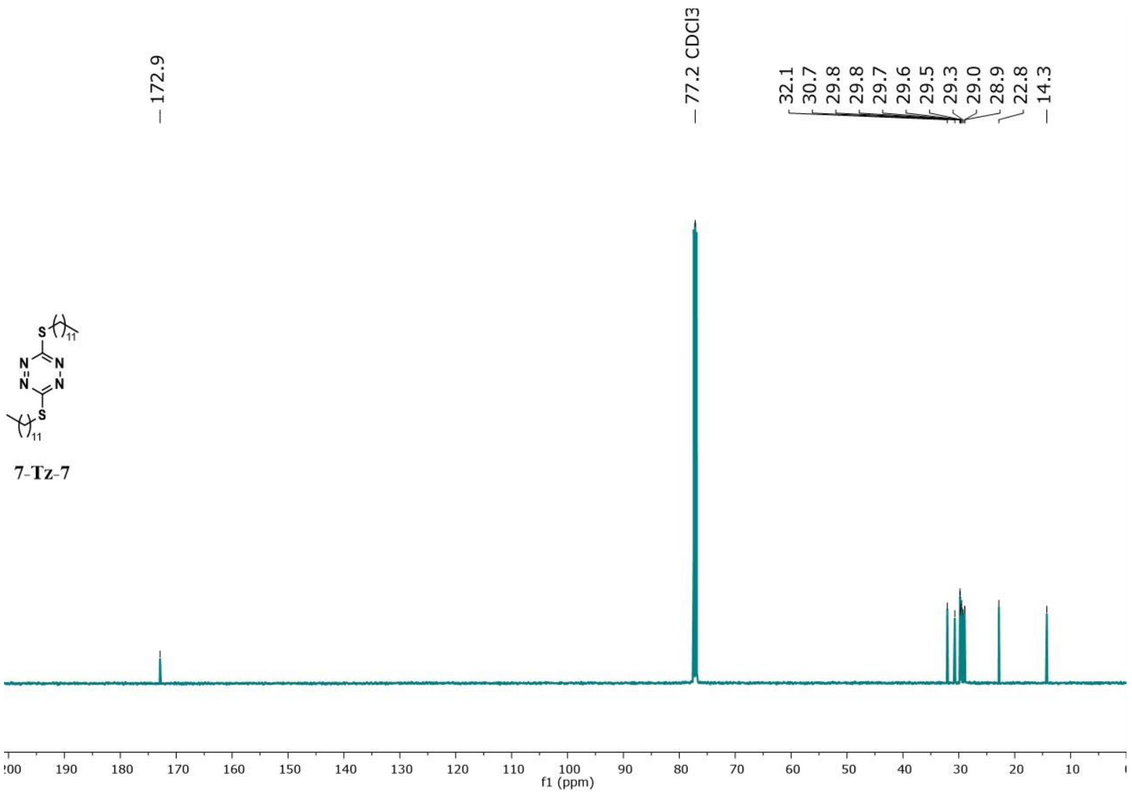

Figure S55. *^{13}C NMR spectrum (126 MHz, CDCl₃, 298 K) of* ***7-Tz-7****.*

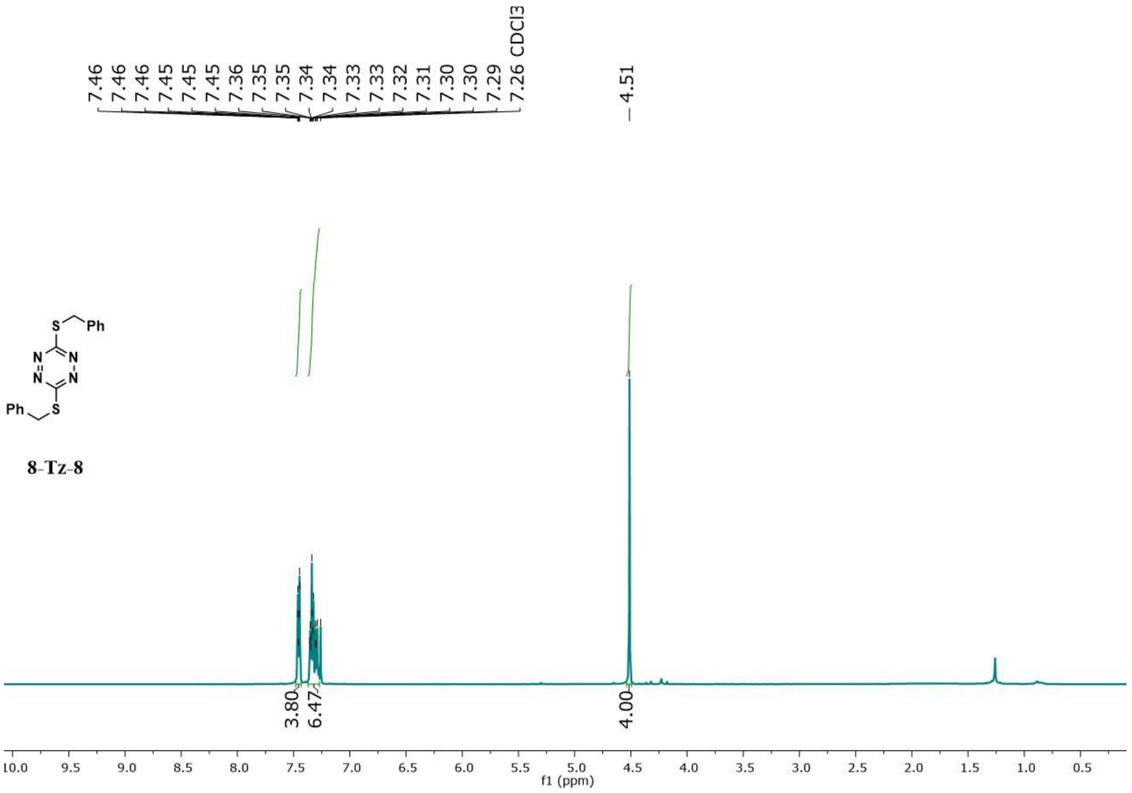

Figure S56. *^{1}H NMR spectrum (500 MHz, CDCl₃, 298 K) of* ***8-Tz-8****.*

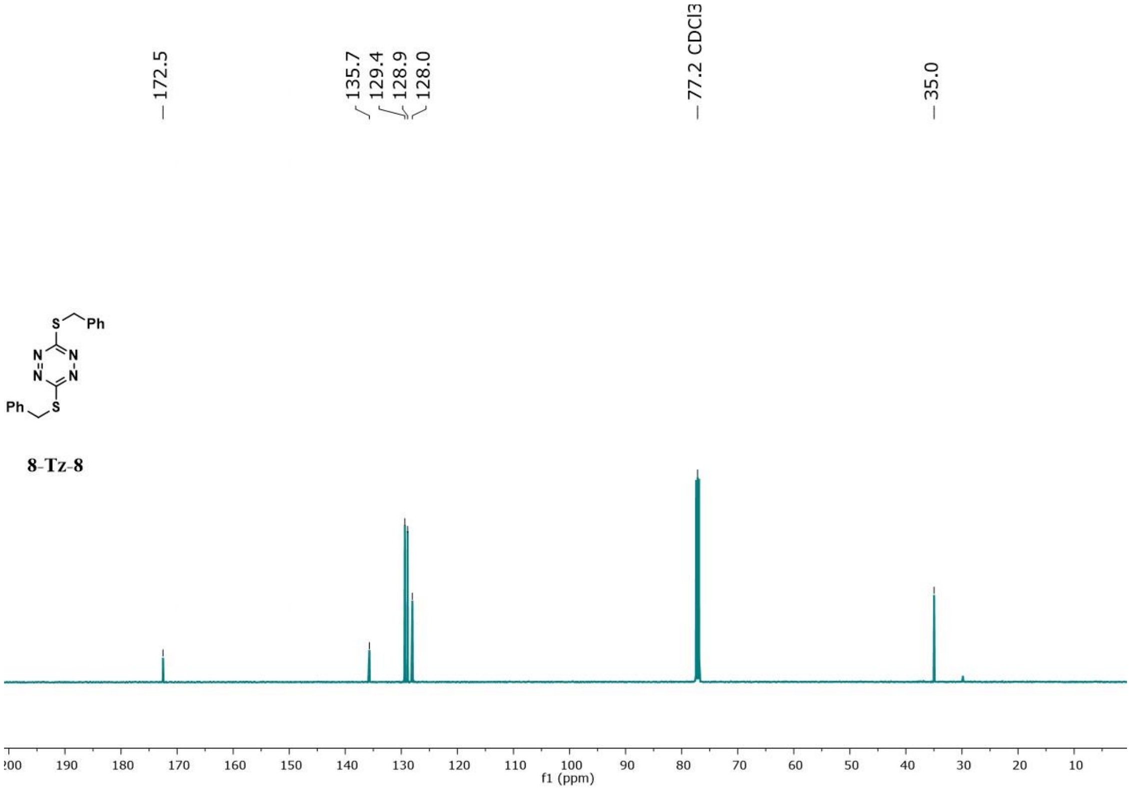

Figure S57. *^{13}C NMR spectrum (126 MHz, CDCl$_3$, 298 K) of* ***8-Tz-8****.*

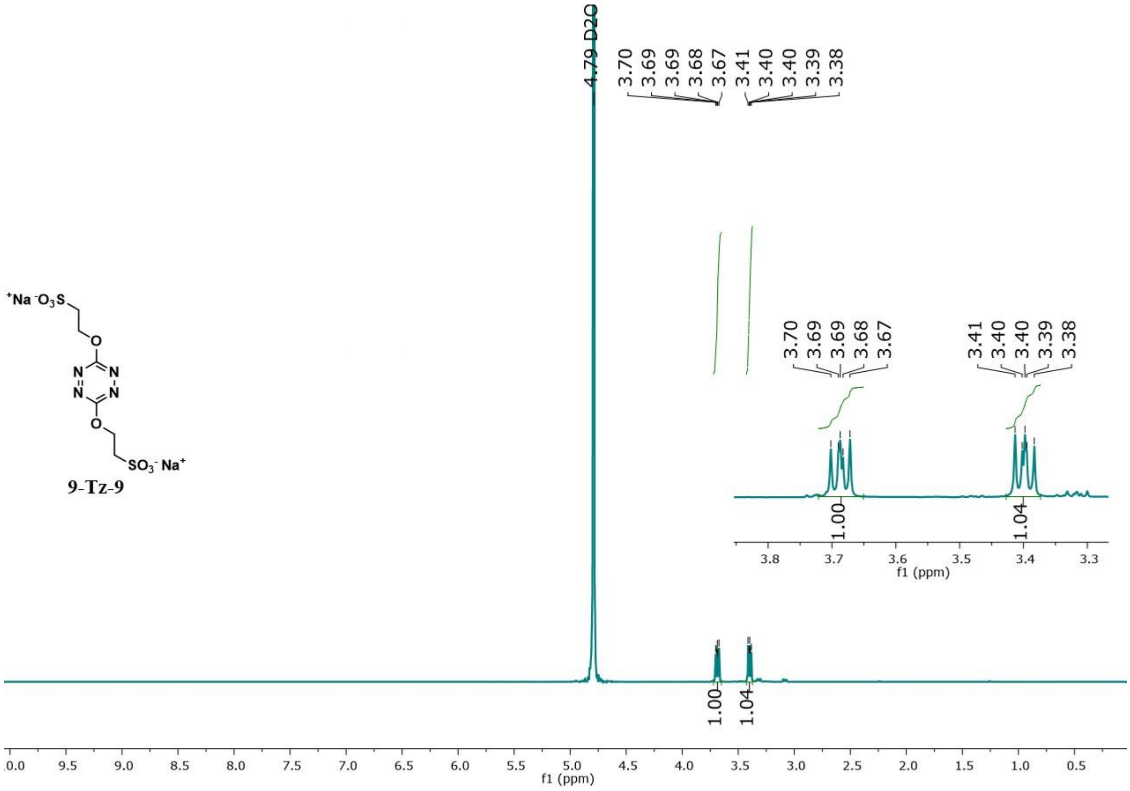

Figure S58. *^{1}H NMR spectrum (500 MHz, D$_2$O, 298 K) of* ***9-Tz-9****.*

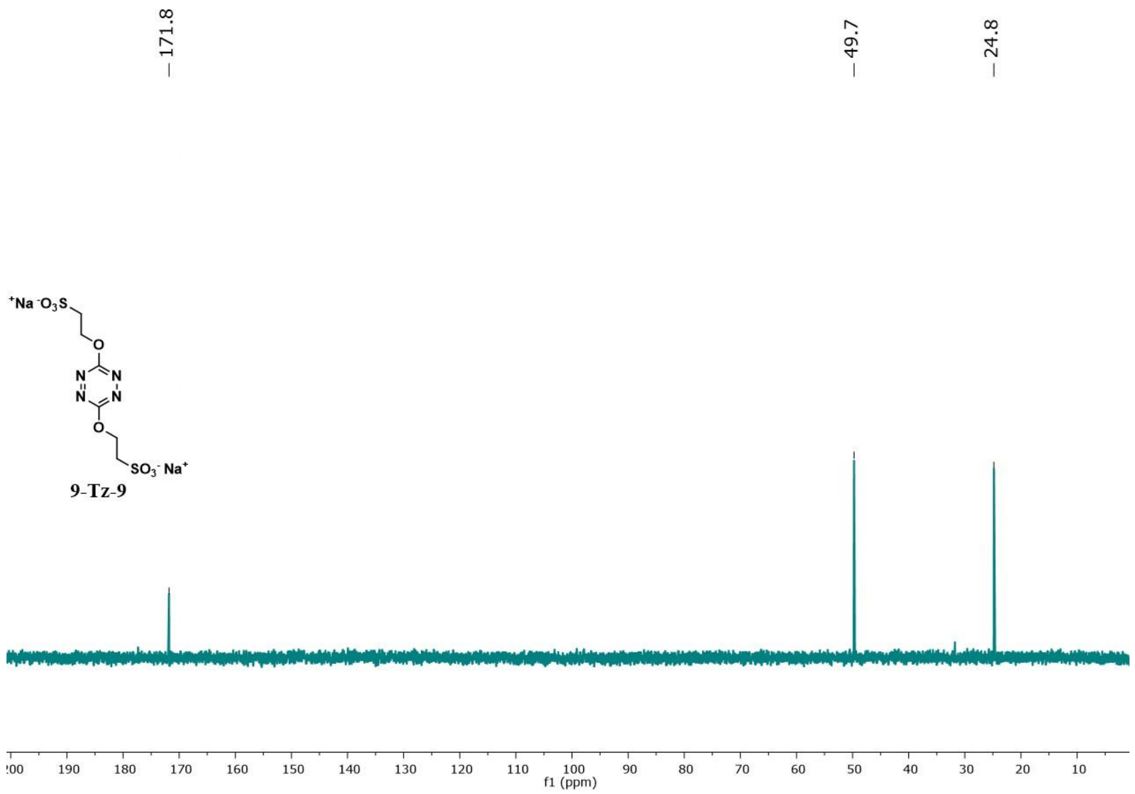

Figure S59. *^{13}C NMR spectrum (126 MHz, D_2O, 298 K) of **9-Tz-9**.*

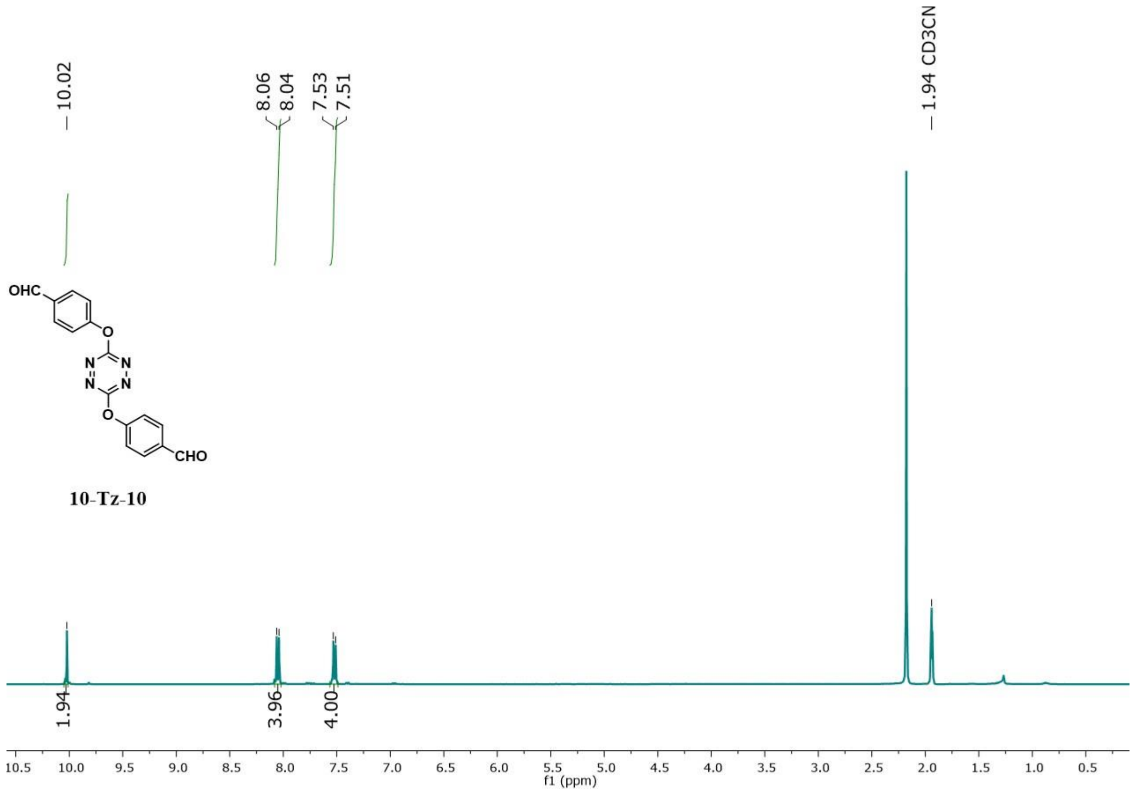

Figure S60. *^{1}H NMR spectrum (400 MHz, CD_3CN, 298 K) of **10-Tz-10**.*

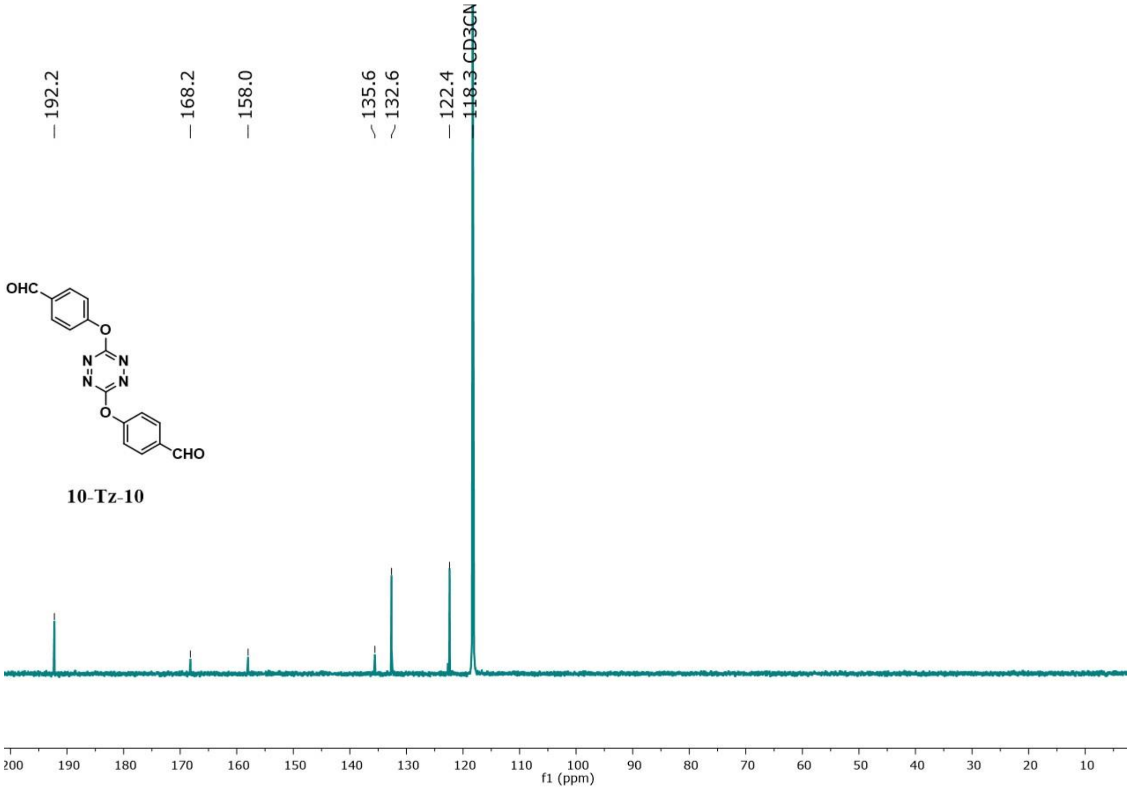

Figure S61. *^{13}C NMR spectrum (101 MHz, CD_3CN, 298 K) of* ***10-Tz-10****.*

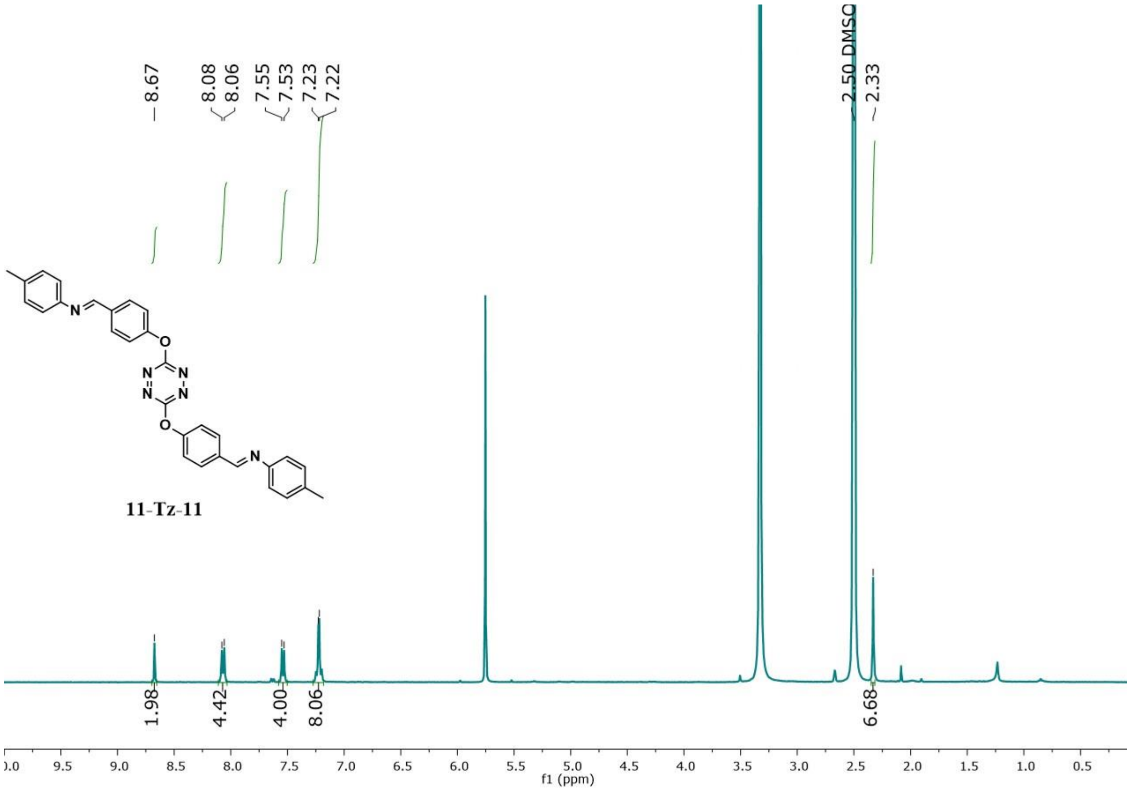

Figure S62. *^{1}H NMR spectrum (500 MHz, DMSO-d^6, 298 K) of* ***11-Tz-11****.*

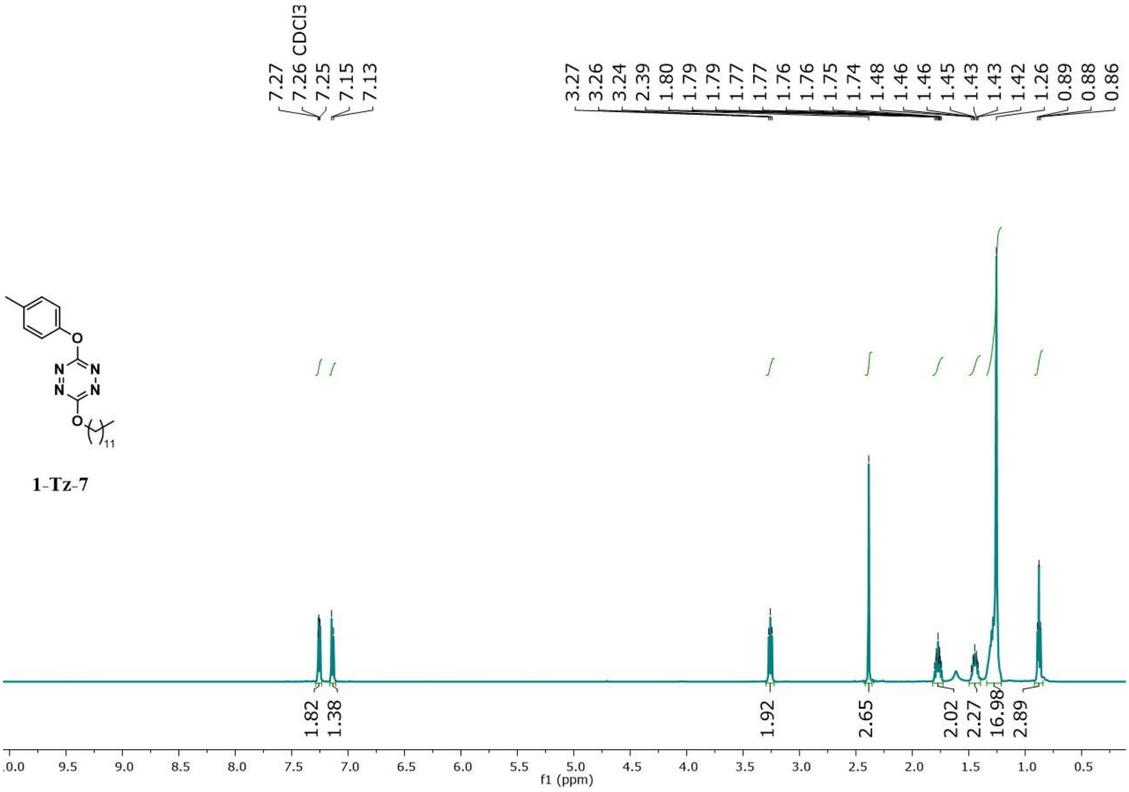

Figure S63. *^{1}H NMR spectrum (500 MHz, $CDCl_3$, 298 K) of **1-Tz-7**.*

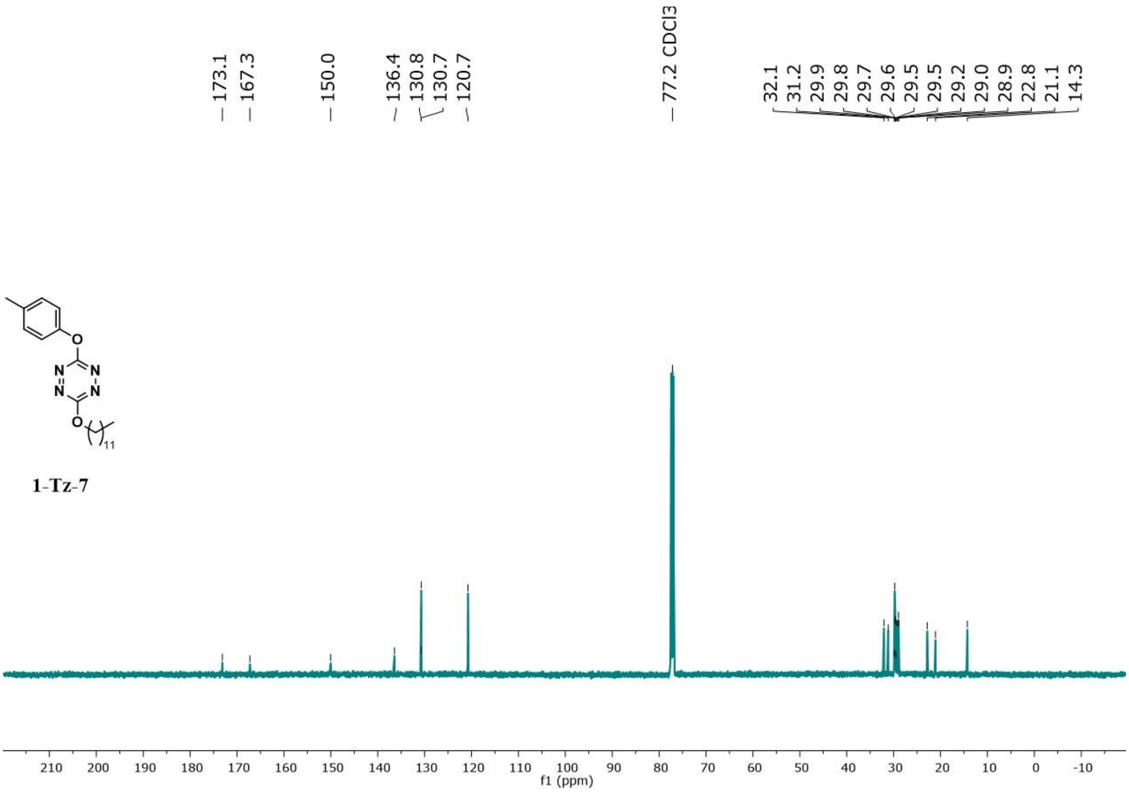

Figure S64. *^{13}C NMR spectrum (126 MHz, $CDCl_3$, 298 K) of **1-Tz-7**.*

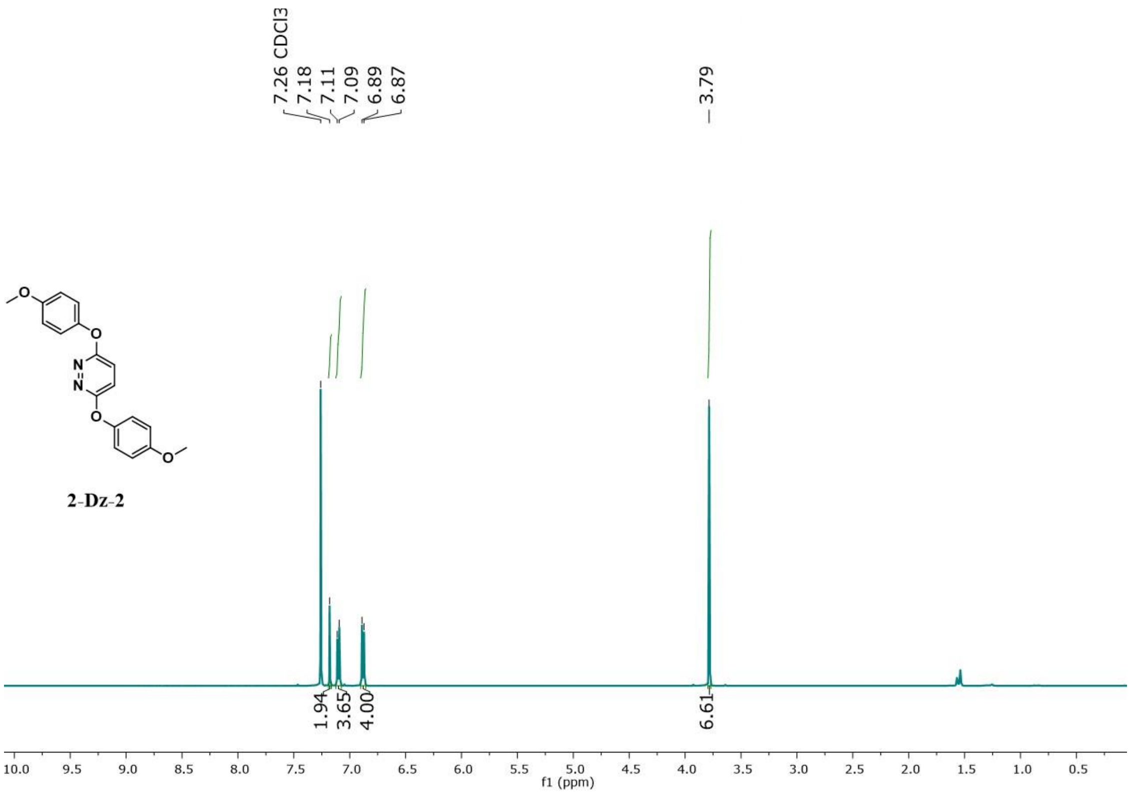

Figure S65. *^{1}H NMR spectrum (500 MHz, $CDCl_3$, 298 K) of **2-Dz-2**.*

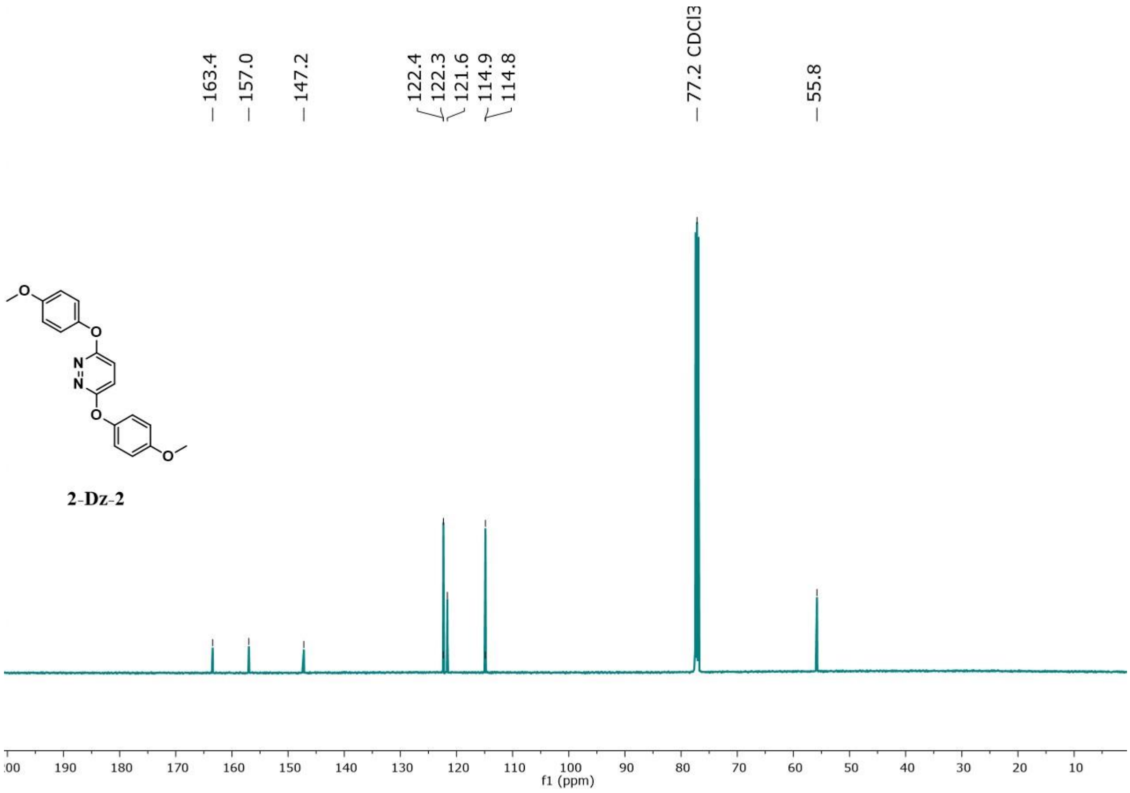

Figure S66. *^{13}C NMR spectrum (126 MHz, $CDCl_3$, 298 K) of **2-Dz-2**.*

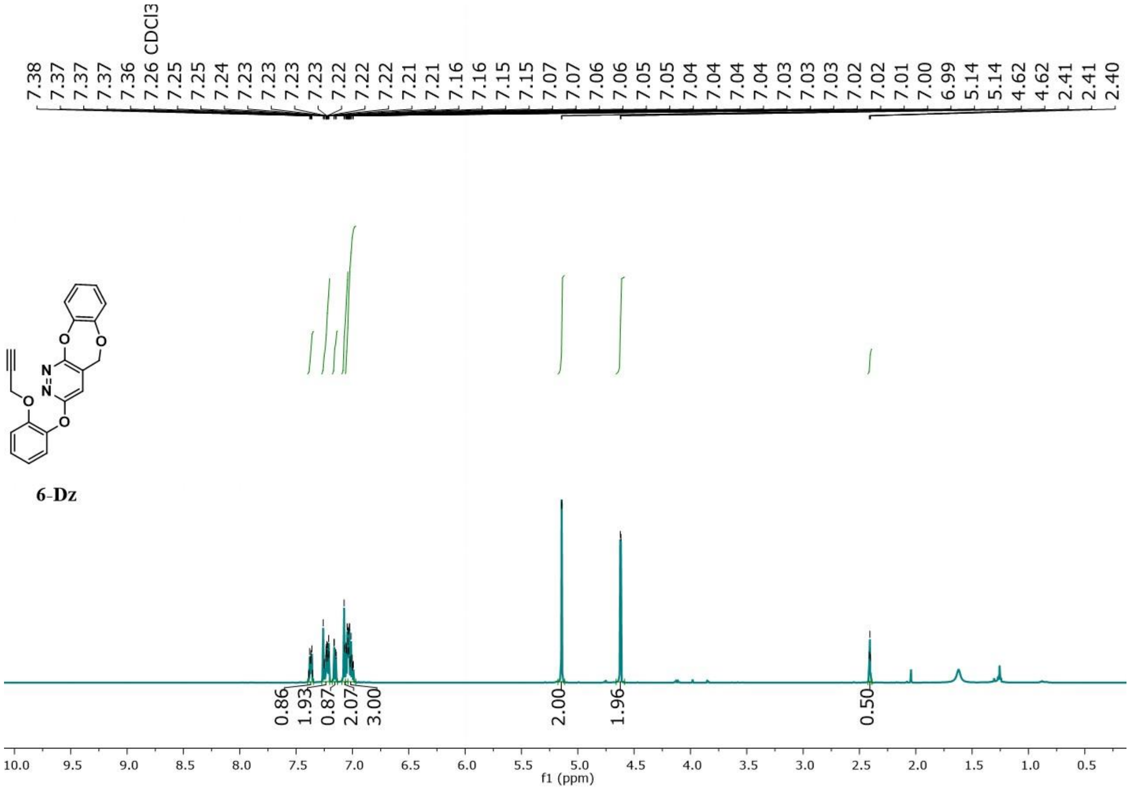

Figure S67. *^{1}H NMR spectrum (500 MHz, $CDCl_3$, 298 K) of* ***6-Dz****.*

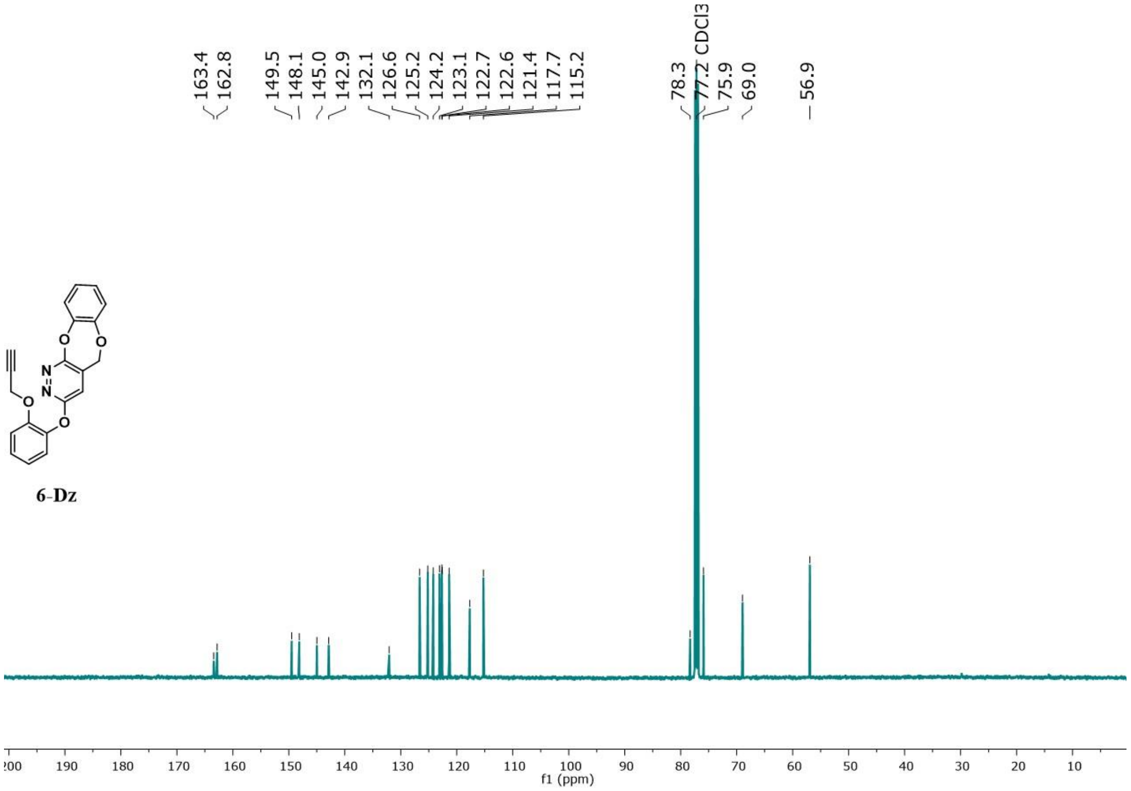

Figure S68. *^{13}C NMR spectrum (126 MHz, $CDCl_3$, 298 K) of* ***6-Dz****.*

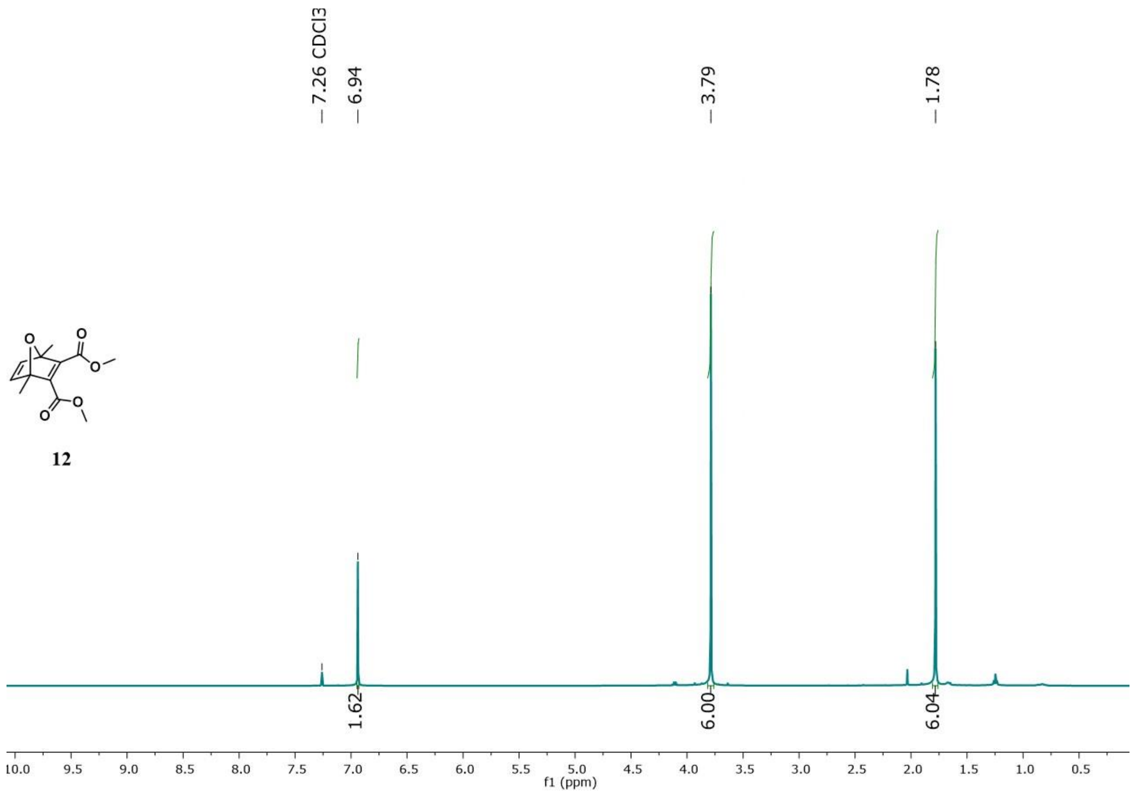

Figure S69. *^{1}H NMR spectrum (500 MHz, $CDCl_3$, 298 K) of* ***12.***

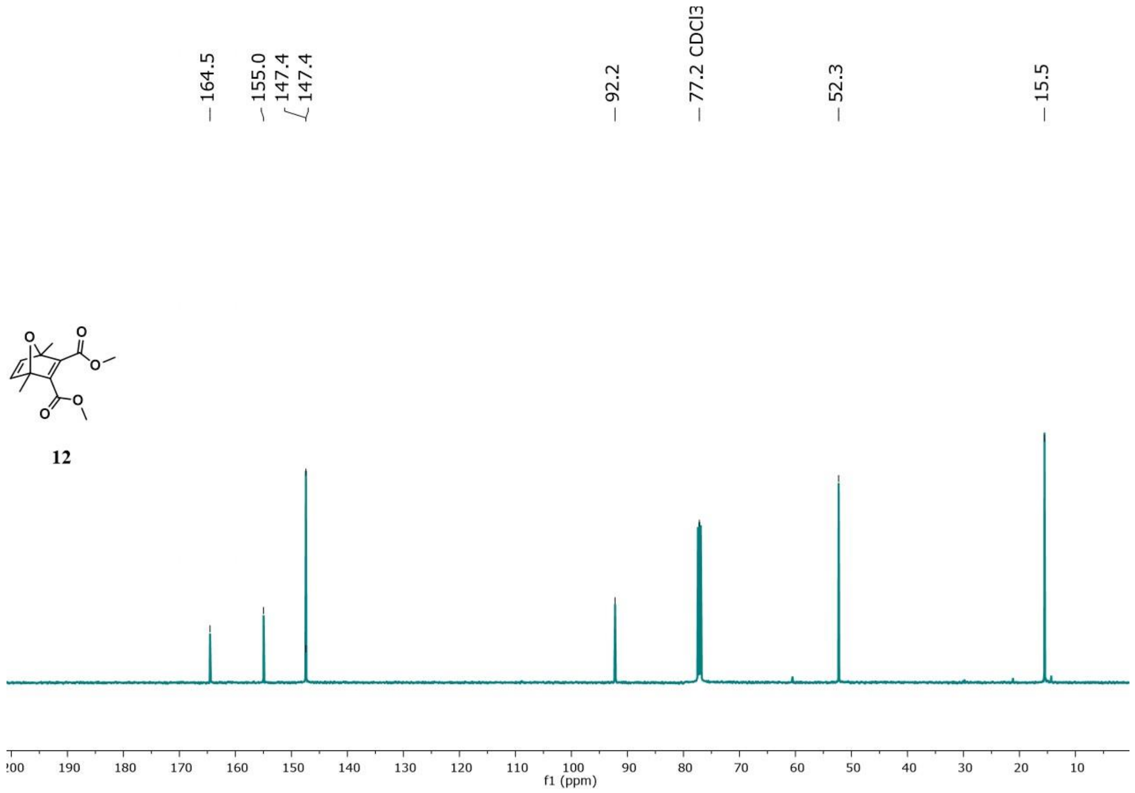

Figure S70. *^{13}C NMR spectrum (126 MHz, $CDCl_3$, 298 K) of* ***12****.*

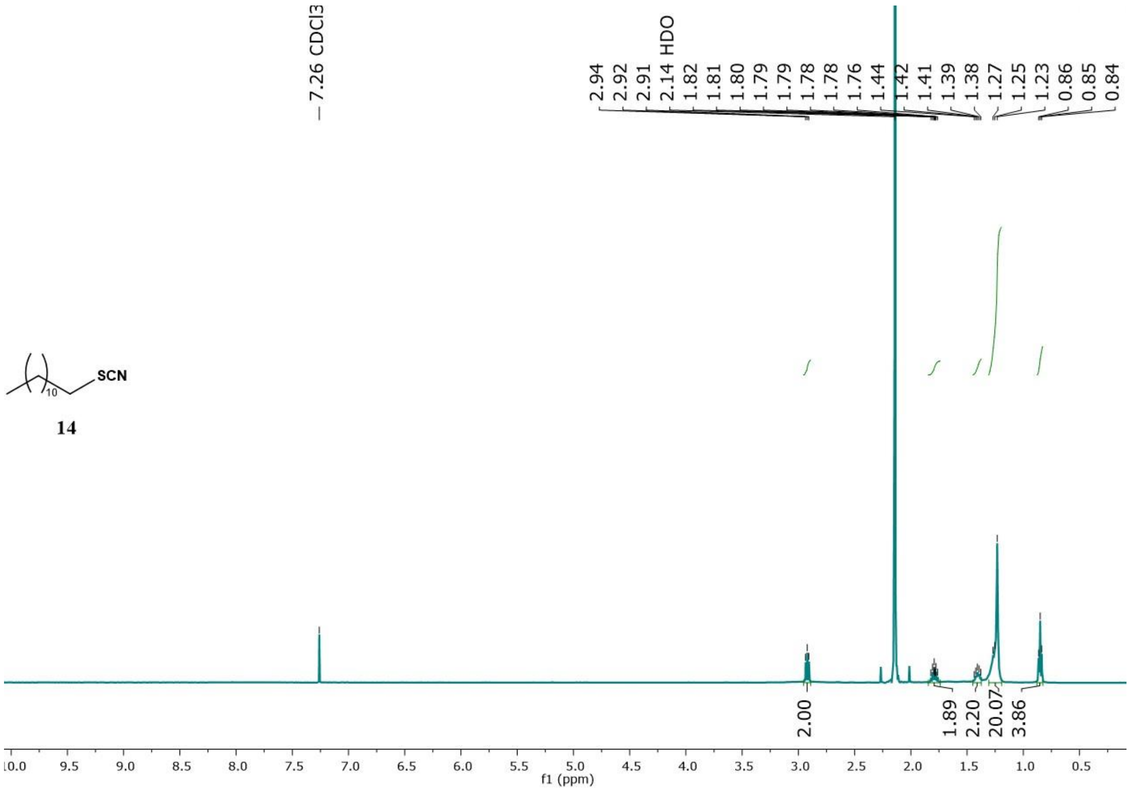

14

Figure S71. *^{1}H NMR spectrum (500 MHz, $CDCl_3$, 298 K) of **14**.*

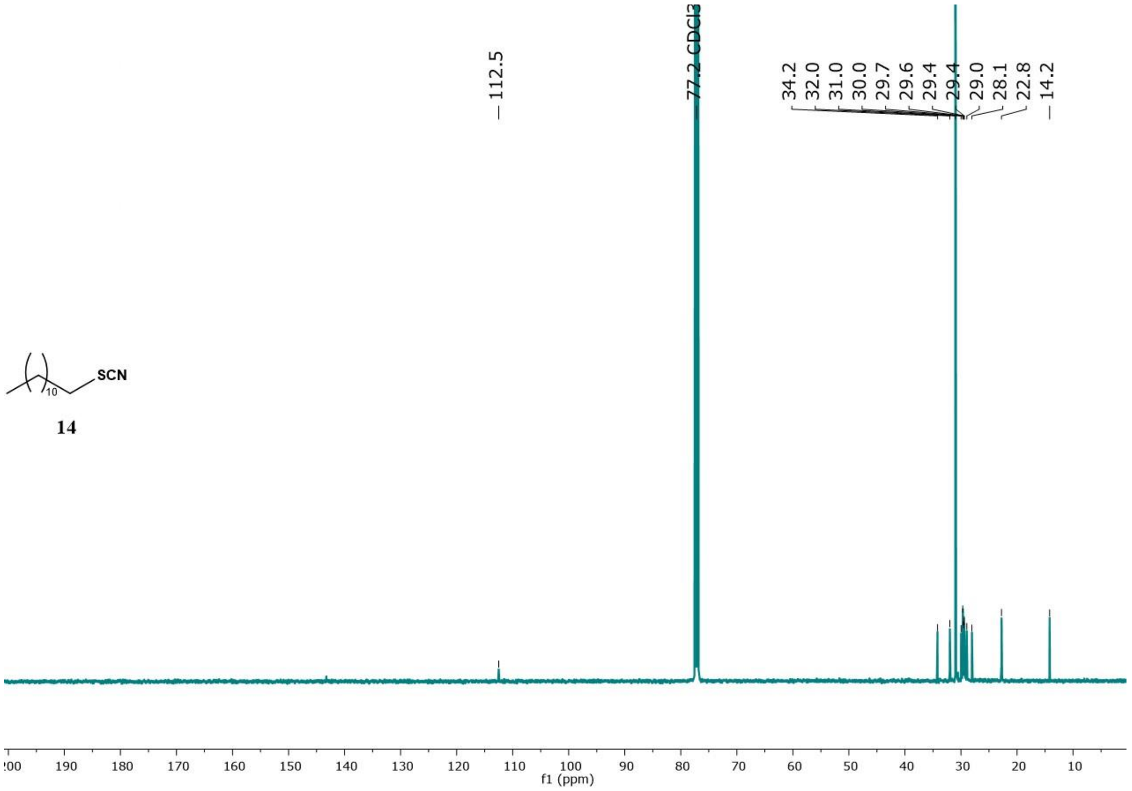

14

Figure S72. *^{13}C NMR spectrum (126 MHz, $CDCl_3$, 298 K) of **14**.*

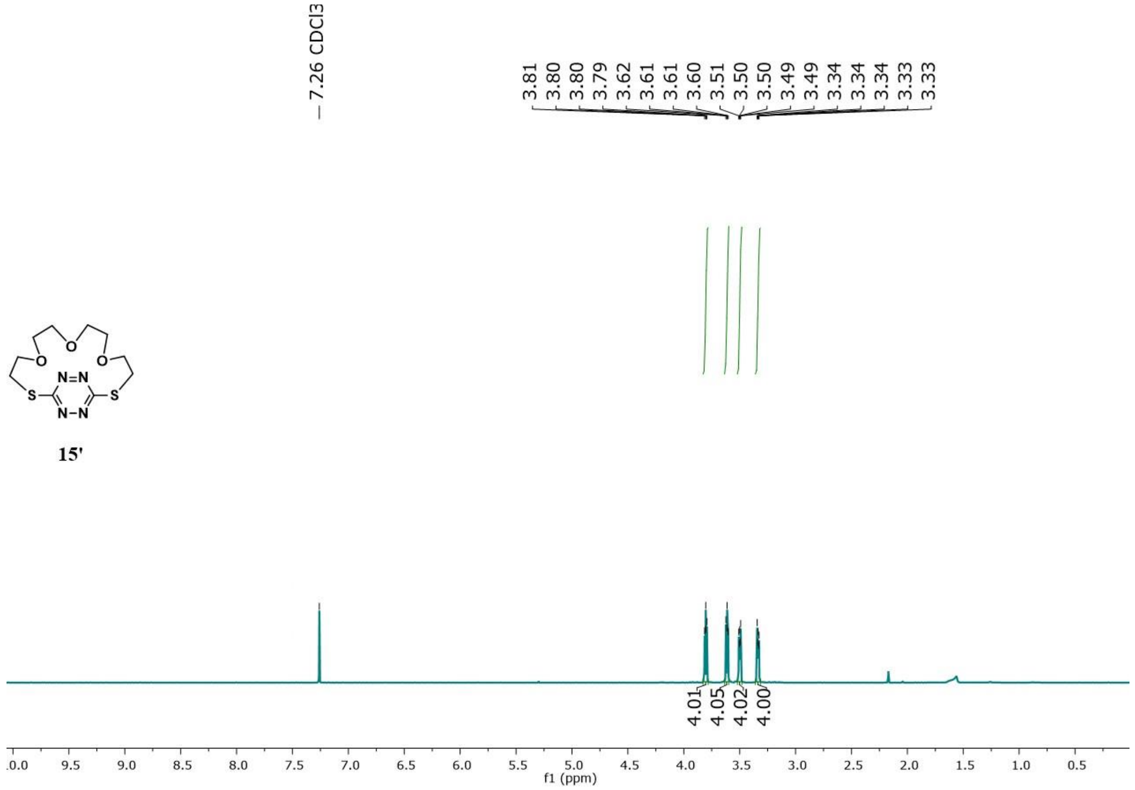

Figure S73. *^{1}H NMR spectrum (500 MHz, $CDCl_3$, 298 K) of* ***15'****.*

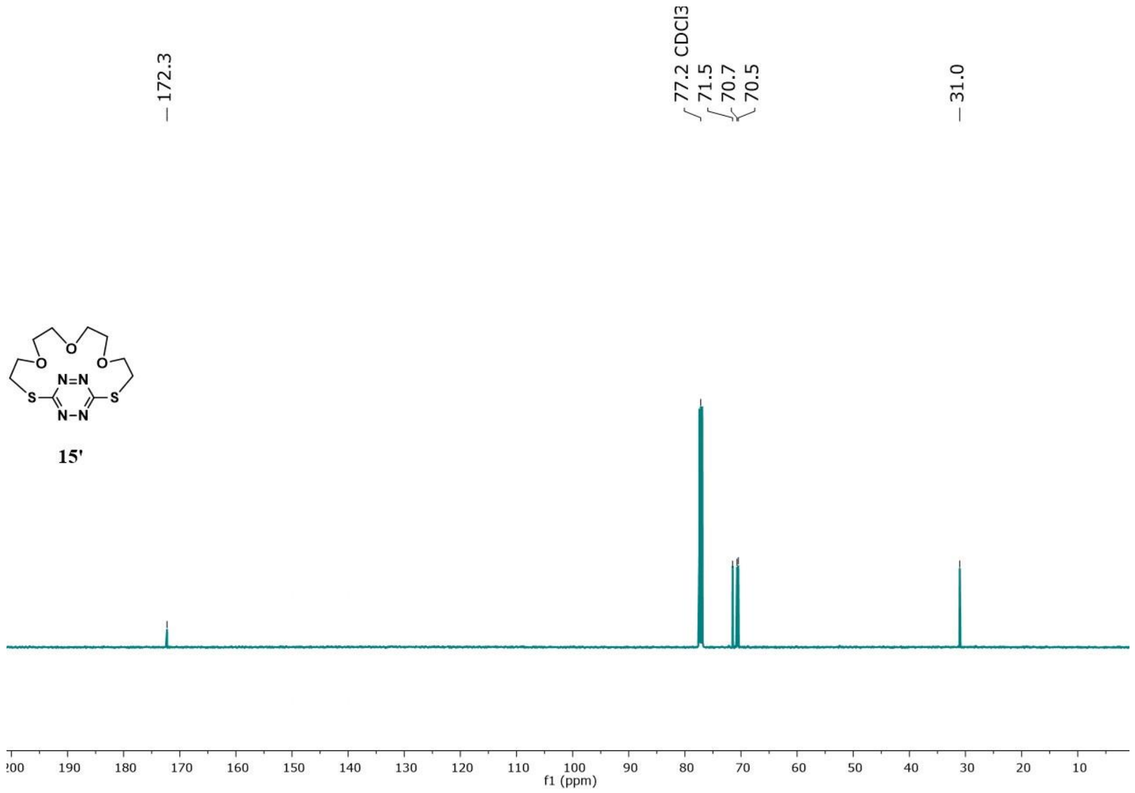

Figure S74. *^{13}C NMR spectrum (126 MHz, $CDCl_3$, 298 K) of* ***15'****.*

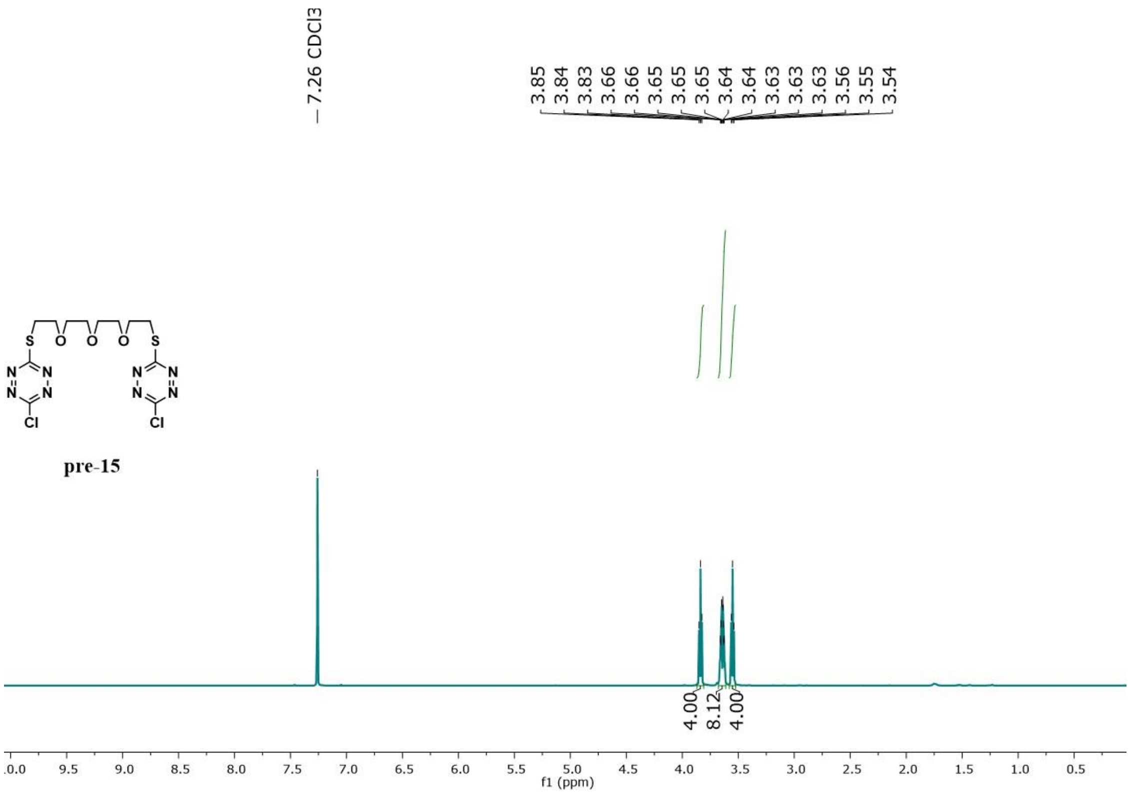

Figure S75. *^{1}H NMR spectrum (500 MHz, $CDCl_3$, 298 K) of* ***pre-15****.*

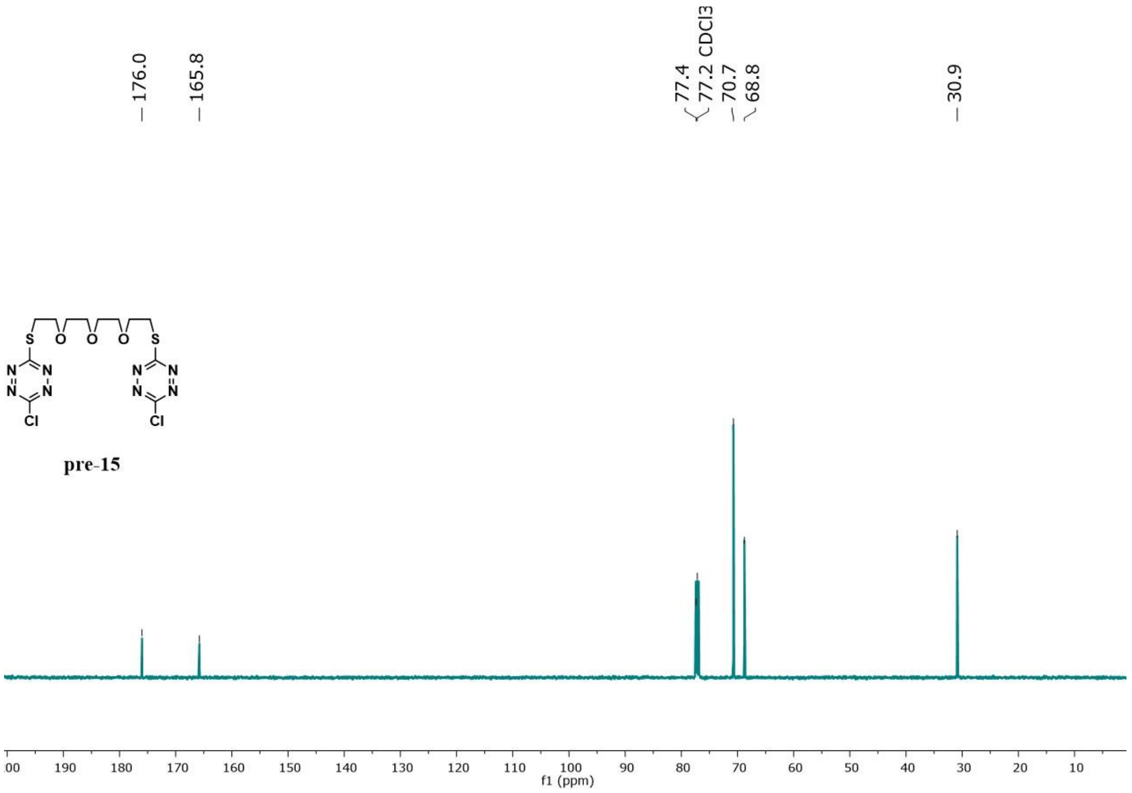

Figure S76. *^{13}C NMR spectrum (126 MHz, $CDCl_3$, 298 K) of* ***pre-15****.*

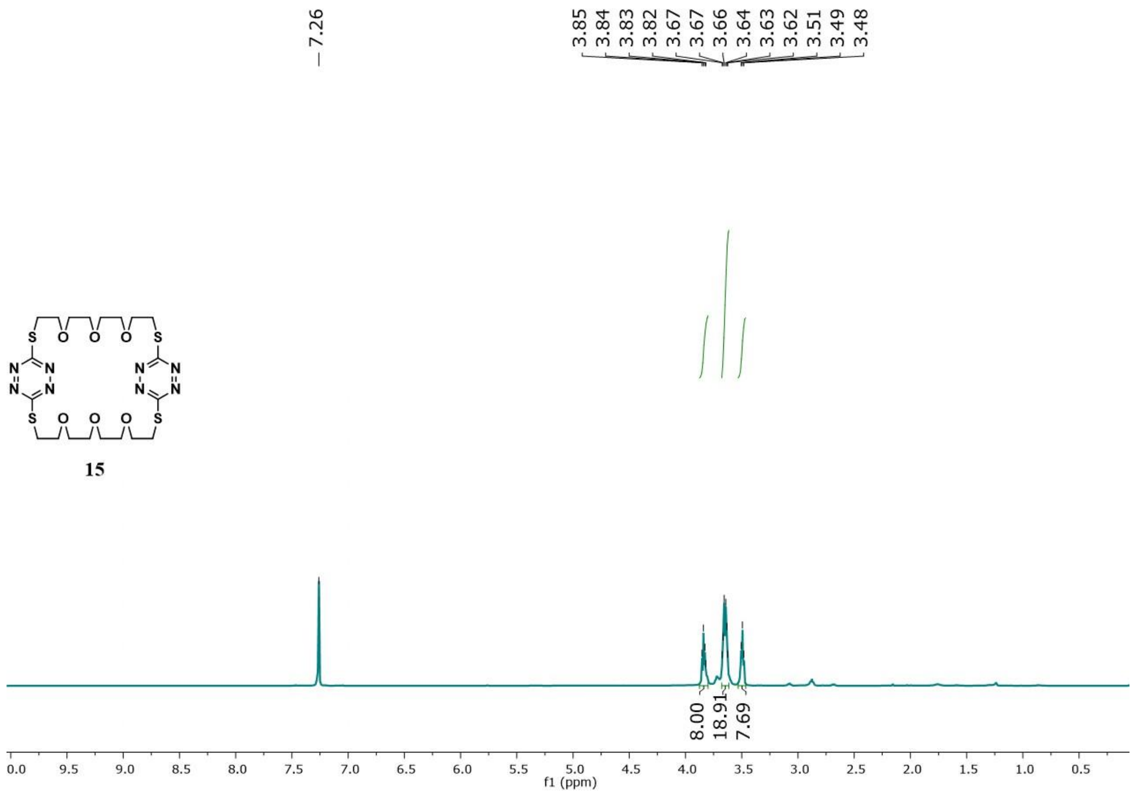

Figure S77. *^{1}H NMR spectrum (500 MHz, $CDCl_3$, 298 K) of* ***15****.*

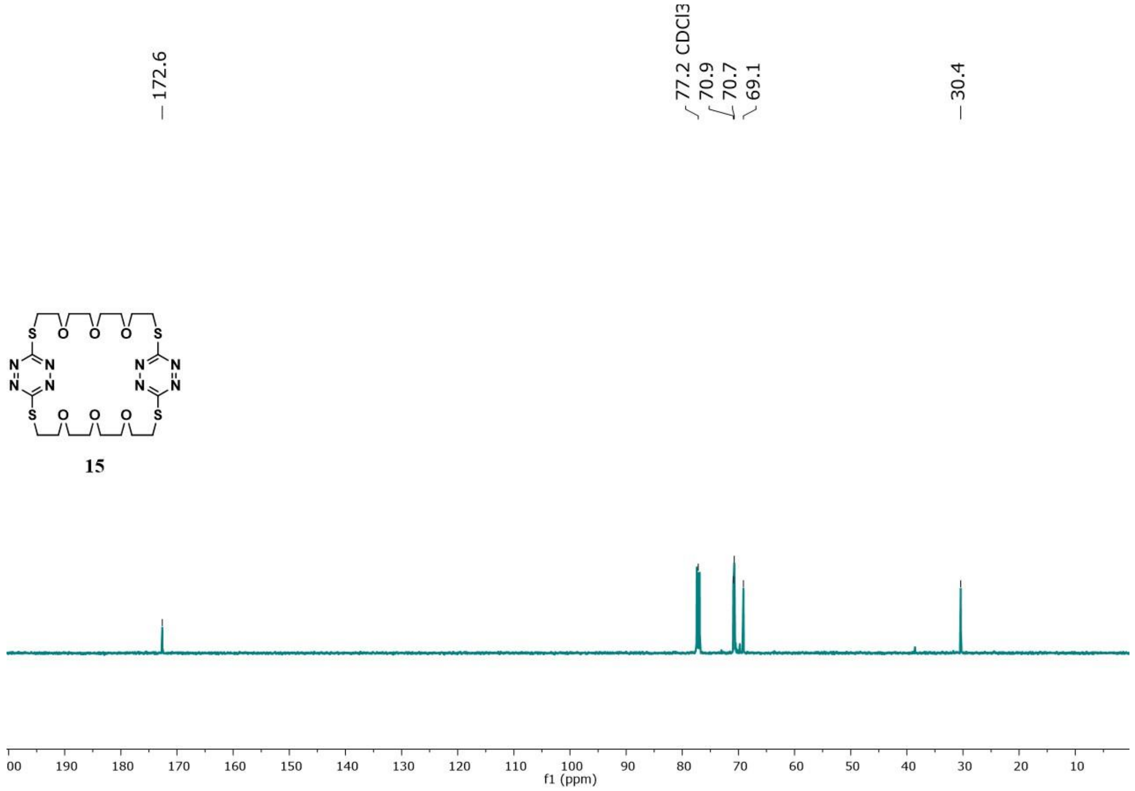

Figure S78. *^{13}C NMR spectrum (126 MHz, $CDCl_3$, 298 K) of* ***15****.*

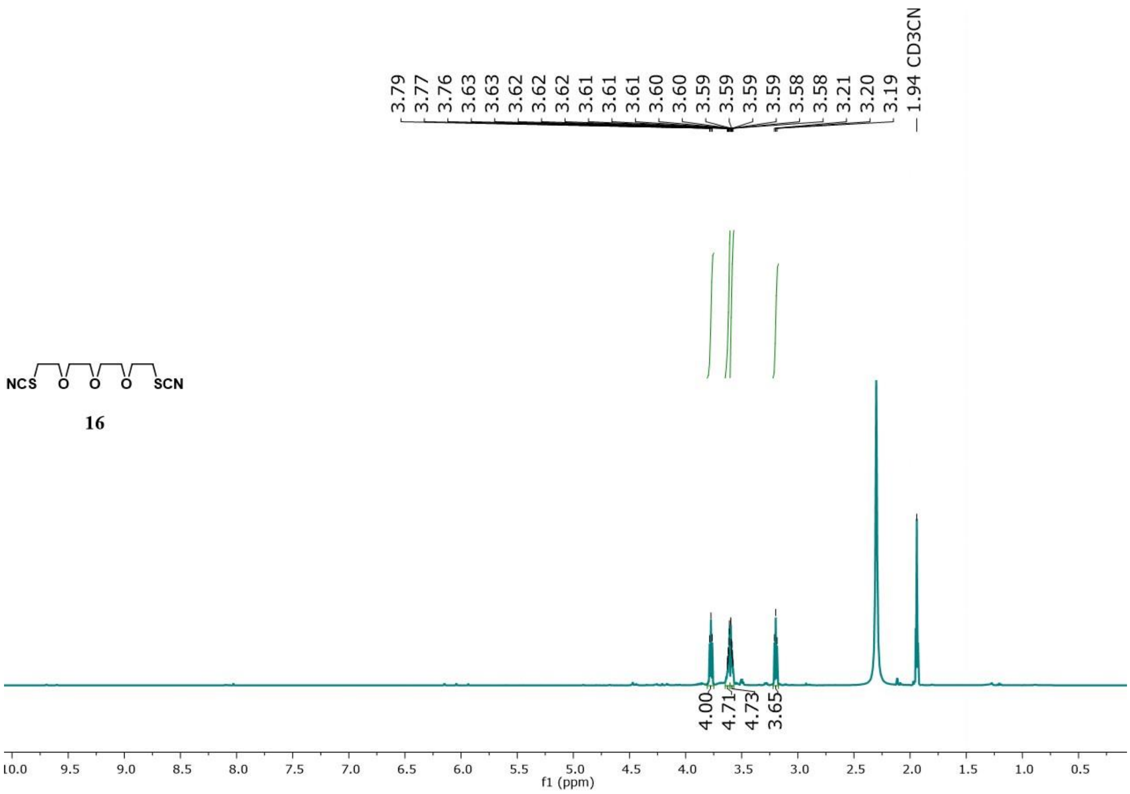

Figure S79. *¹H NMR spectrum (500 MHz, CDCl$_3$, 298 K) of* ***16****.*

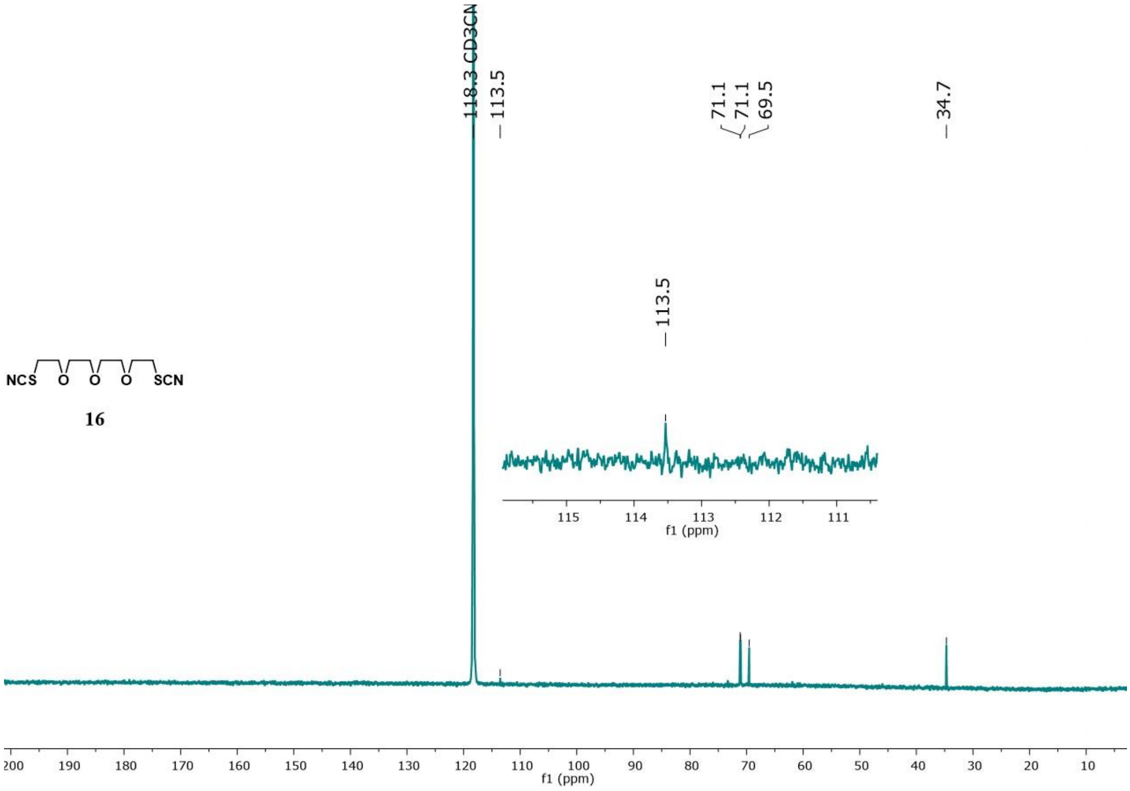

Figure S80. *¹³C NMR spectrum (126 MHz, CDCl$_3$, 298 K) of* ***16****.*

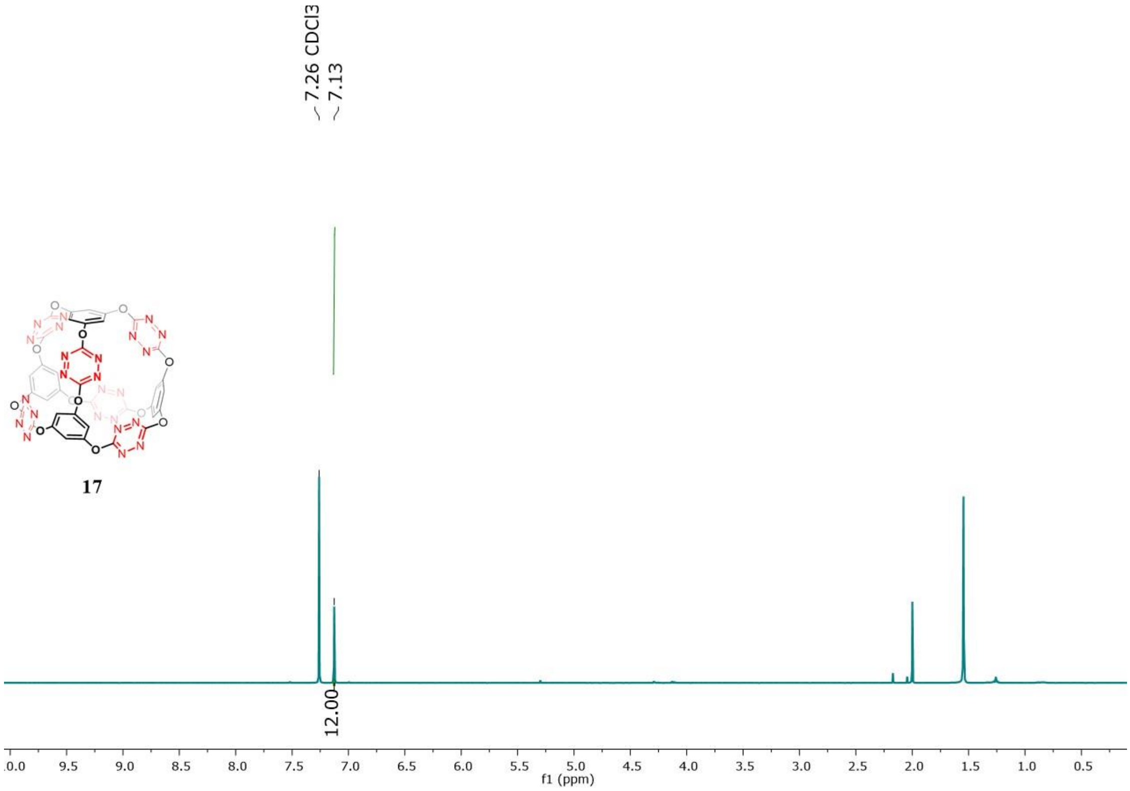

Figure S81. *^{1}H NMR spectrum (400 MHz, CDCl$_3$, 298 K) of* ***17***.

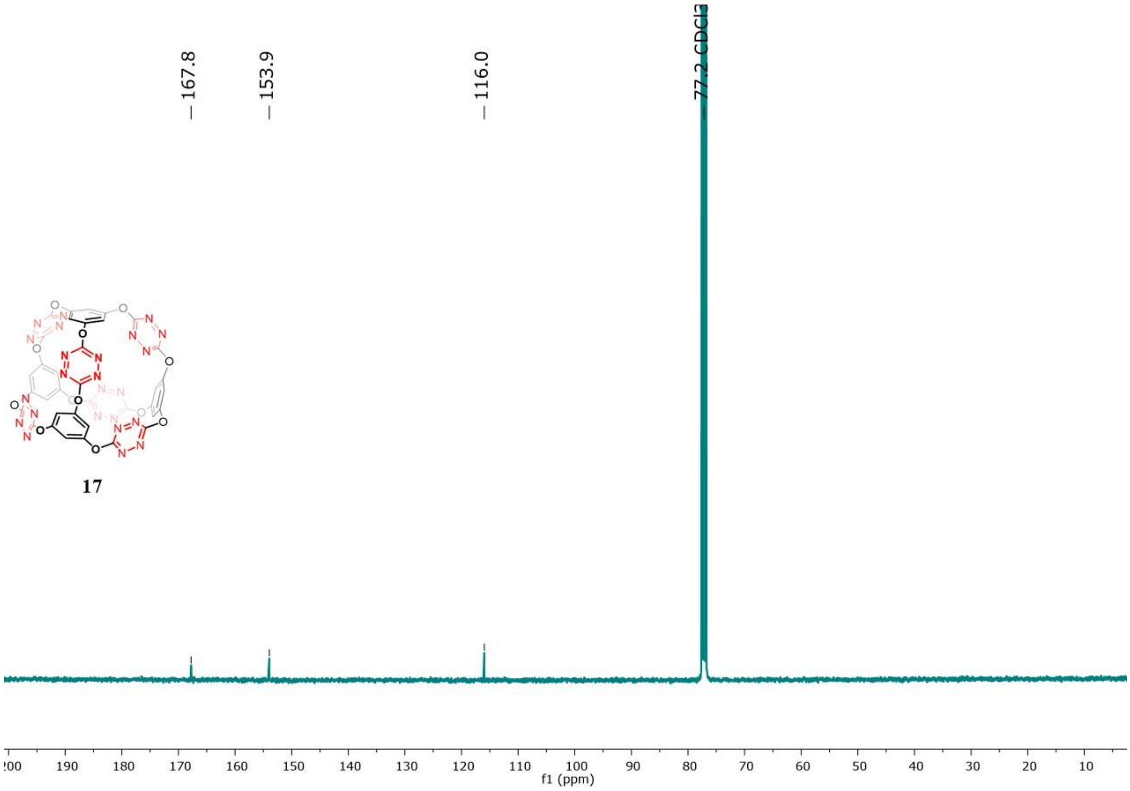

Figure S82. *^{13}C NMR spectrum (101 MHz, CDCl$_3$, 298 K) of* ***17***.

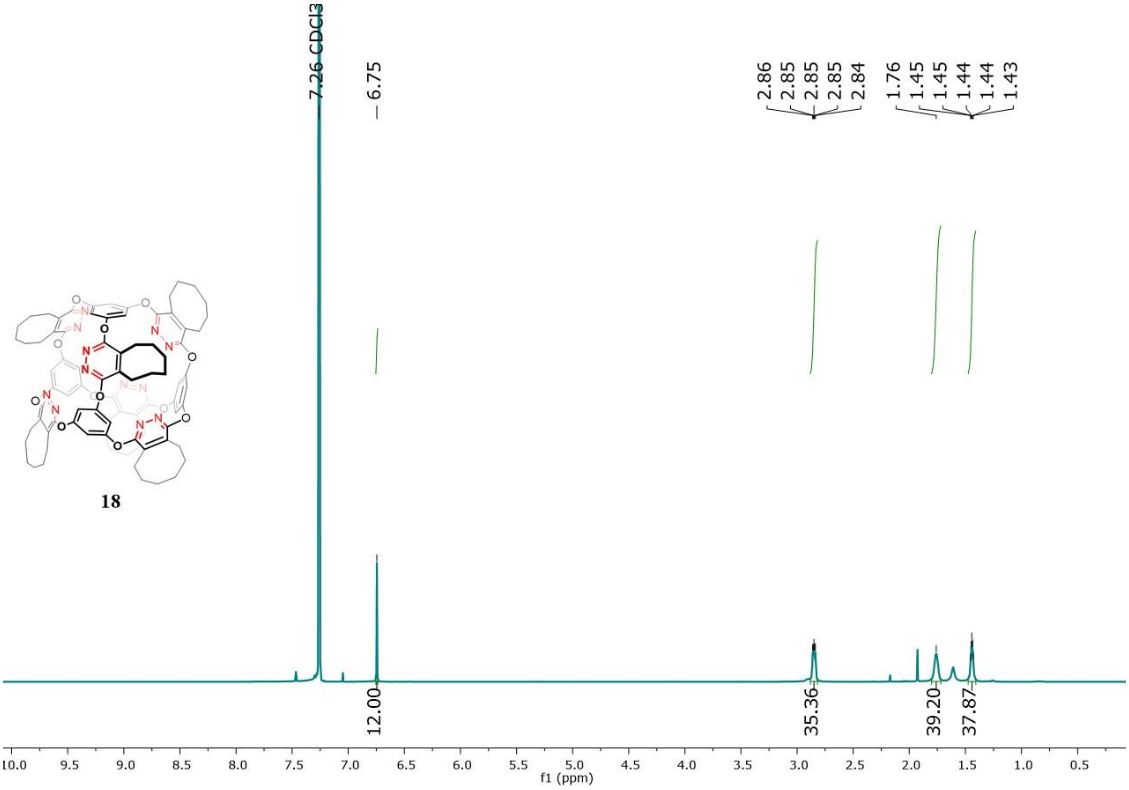

Figure S83. *^{1}H NMR spectrum (500 MHz, CDCl$_3$, 298 K) of* ***18****.*

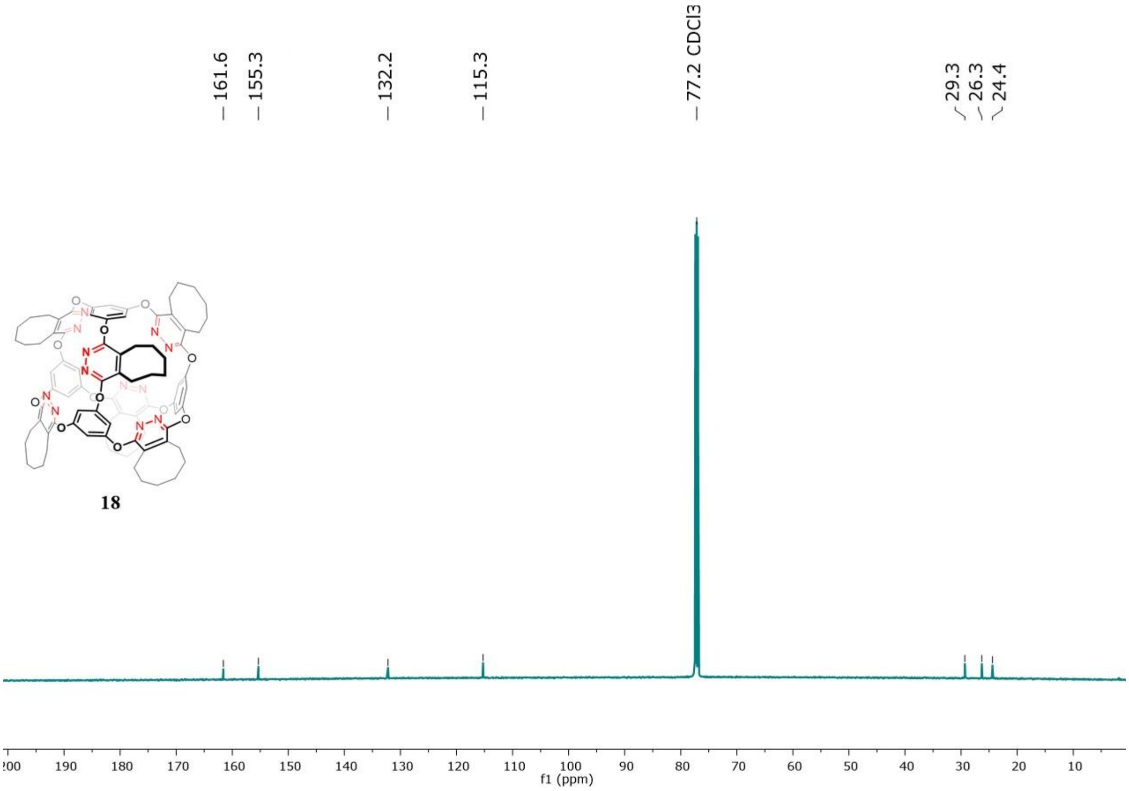

Figure S84. *^{13}C NMR spectrum (126 MHz, CDCl$_3$, 298 K) of* ***18****.*

References.

[1] M. D. Coburn, G. A. Buntain, B. W. Harris, M. A. Hiskey, K.-Y. Lee, D. G. Ott, *J. Heterocycl. Chem.* **1991**, *28*, 2049.

[2] D. E. Chavez, M. A. Hiskey, *J. Heterocycl. Chem.* **1998**, *35*, 1329.

[3] M. D. Helm, A. Plant, J. P. A. Harrity, *Org. Biomol. Chem.* **2006**, *4*, 4278.

[4] Y.-H. Gong, F. Miomandre, R. Meallet-Renault, S. Badre, L. Galmiche, J. Tang, P. Audebert, G. Clavier, *Eur. J. Org. Chem.* **2009**, *2009*, 6121.

[5] R. E. Bagge, T. C. Mauldin, D. J. Boday, B. M. Kobilka, D. A. Loy, *Chem. Mater*. **2017**, *29*, 7953.

[6] D. J. Keddie, J. B. Grande, F. Gonzaga, M. A. Brook, T. R. Dargaville, *Org. Lett*. **2011**, *13*, 6006-6009.

[7] D. A. Roberts, B. S. Pilgrim, G. Sirvinskaite, T. K. Ronson, J. R. Nitschke, *J. Am. Chem. Soc*. **2018**, *140,* 9616.

[8] Rigaku Oxford Diffraction, CrysAlisPro Software system, version 1.171.40.53, Rigaku Corporation, Wroclaw, Poland, 2019.

[9] G. M. Sheldrick, *Acta Crystallogr. Sect. A,* **2015**, *A71*, 3-8.

[10] G. M. Sheldrick, *Acta Crystallogr. Sect. C,* **2015**, *C71*, 3-8.

[11] O. V. Dolomanov, L. J. Bourhis, R. J. Gildea, J. A. K. Howard, H. Puschmann, *J. Appl. Cryst.*, **2009**, *42*, 339-341.
